# Neutrophil Extracellular Traps protein composition is specific for patients with Lupus nephritis and includes methyl-oxidized αenolase (methionine sulfoxide 93)

**DOI:** 10.1038/s41598-019-44379-w

**Published:** 2019-05-28

**Authors:** Maurizio Bruschi, Andrea Petretto, Laura Santucci, Augusto Vaglio, Federico Pratesi, Paola Migliorini, Roberta Bertelli, Chiara Lavarello, Martina Bartolucci, Giovanni Candiano, Marco Prunotto, Gian Marco Ghiggeri

**Affiliations:** 1 Laboratory of Molecular Nephrology, IRCCS Istituto Giannina Gaslini, Genoa, Italy; 2 Core Facilities-Proteomics Laboratory, IRCCS Istituto Giannina Gaslini, Genoa, Italy; 3 Department of Biomedical Experimental and Clinical Sciences “Mario Serio”, University of Firenze, and Meyer Children’s Hospital, Firenze, Italy; 4 Department of Clinical and Experimental Medicine, Clinical Immunology Unit, University of Pisa, Pisa, Italy; 5 School of Pharmaceutical Sciences, University of Geneva, Geneva, Switzerland; 6 Division of Nephrology, Dialysis, and Transplantation, Scientific Institute for Research and Health Care (IRCCS), IRCCS Istituto Giannina Gaslini, Genoa, Italy

**Keywords:** Autoimmunity, Kidney diseases

## Abstract

NETs constitute a network of DNA and proteins released by neutrophils in response to infectious and immunologic triggers. NET proteins are recognized as autoantigens in ANCA vasculitis; limited knowledge is available in other autoimmune pathologies. The composition of NETs produced *ex vivo* by resting and Phorbol-myristate acetate (PMA) stimulated neutrophils was analyzed by high-throughput Fusion Orbitrap technology in 16 patients with Systemic Lupus Erythematosus/Lupus nephritis (9 SLE/7 LN) and in 11 controls. Seven-hundred proteins were characterized and specific fingerprints discriminated LN from SLE. We focused on methyl-oxidized αenolase (methionine sulfoxide 93) that was markedly increased in NETs from LN and was localized in NET filaments in tight connection and outlying DNA. The isotype of anti-αenolase antibodies was IgG2 in LN and IgG4 in other autoimmune glomerulonephritis (Membranous Nephropathy, MN); serum anti-αenolase IgG2 were higher in LN than in SLE and absent in MN. The same IgG2 antibodies recognized 5 epitopes of the protein one containing methionine sulphoxide 93. In conclusion, specific NET protein fingerprints characterize different subsets of SLE; methyl-oxidized αenolase is over-expressed in LN. Circulating anti-αenolase IgG2 recognize the oxidized epitope and are high in serum of LN patients. Post-translational modified NET proteins contribute to autoimmunity in patients with LN.

## Introduction

The release of Neutrophil Extracellular Traps (or NETs) is a defense strategy to limit exogenous infections^1–3^. Formation of a physical network by nuclear chromatin, entrapping pathogens, is the fundamental step of that process^1,4,5^. Generation of superoxide oxygen^6^ and activation of several kinases^7–9^ are the basic steps inducing NETosis. Such process can be reproduced *in vitro* via activation of NADPH-oxidase by Phorbol-myristate acetate (PMA, a phorbol ester similar to diacylglycerol)^10–12^ that triggers a downstream cascade involving several molecules (*i.e*., c-Raf, MEK, Akt, ERK)^7–9^. Induction of neutrophil-elastase is a second key event in NET formation: neutrophil-elastase dissembles F-actin, shuttles to the nucleus where de-condenses chromatin and digests membranes enabling extracellular DNA release^11,12^. NETs can also be induced in sterile conditions: by cytokines, immune-complexes and autoantibodies; this represents a second type of NETosis^13^.

Previous studies demonstrated that both DNA and post-translational modified proteins constitute NETs^14–16^ and that both become target auto-antigens^17–19^ contributing to break tolerance in clinically relevant auto-inflammatory and autoimmune conditions^20^. Small vessel vasculitis^21,22^ and anti-phospholipid syndrome are main examples^23,24^. In vasculitis, myeloperoxidase (MPO) and proteinase-3 (PR3), the two auto-antigens recognized by anti-neutrophil cytoplasm antibodies (ANCA), are, in fact, released from NETs^21^. Systemic Lupus Erythematosus (SLE)^25^ is another condition in which NETs have been shown to be formed^13,26,27^ and NETs implication in SLE pathogenesis hypothesized.

Though NETosis is recognized as a key protective and pathophysiological mechanism, no studies have utilized high troughput proteomics to identify and characterize structural features of the soluble protein components of the NETs complex. The present study investigates protein composition of NETs and their post-translational modifications in patients affected by clinical relevant SLE. Patients were subdivided according to the presence of renal complications (Lupus nephritis, LN), the most severe long term evolution of SLE.

## Results

### NETs composition in different clinical settings

#### Controls, SLE and LN

**Figure 1:**
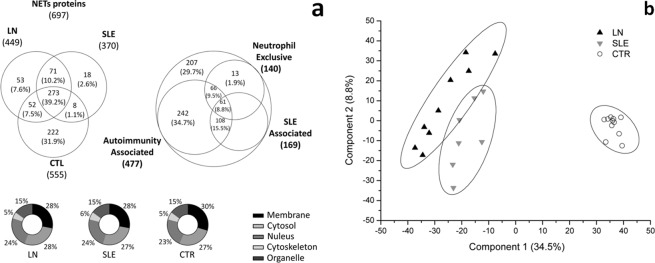
(**a**) Venn diagram and Pie-chart of Gene Ontology classificationfor cellular components of NETs isolated from ‘*ex vivo*’ PMA stimulated neutrophils: Venn diagram of identified NET proteins; the whole protein NET complexes had overlap between proteins recognized as specific components of normal neutrophil and/or with proteins already associated with autoimmunity and specifically with SLE. Of note, 66 of autoimmunity associated and 108 of autoimmunity/SLE associated proteins were exclusively expressed in neutrophils. Numbers, represent the distinct proteins in the respective overlapping and not-overlapping areas. Pie-chart of sub-cellular localization showing high identity in cellular component classification among different groups. (**b**) Multidimensional scaling (MDS) analysis of NETs. Two-dimensional scatter plot of MDS analysis of Control (circle), LN (Up-triangle) and SLE (Down-triangle) NET proteins.Ellipses (corresponding to cluster area at 95% of CI) show three clusters corresponding to control, LN and SLE samples, with partial overlapping between SLE and LN.

**Figure 2:**
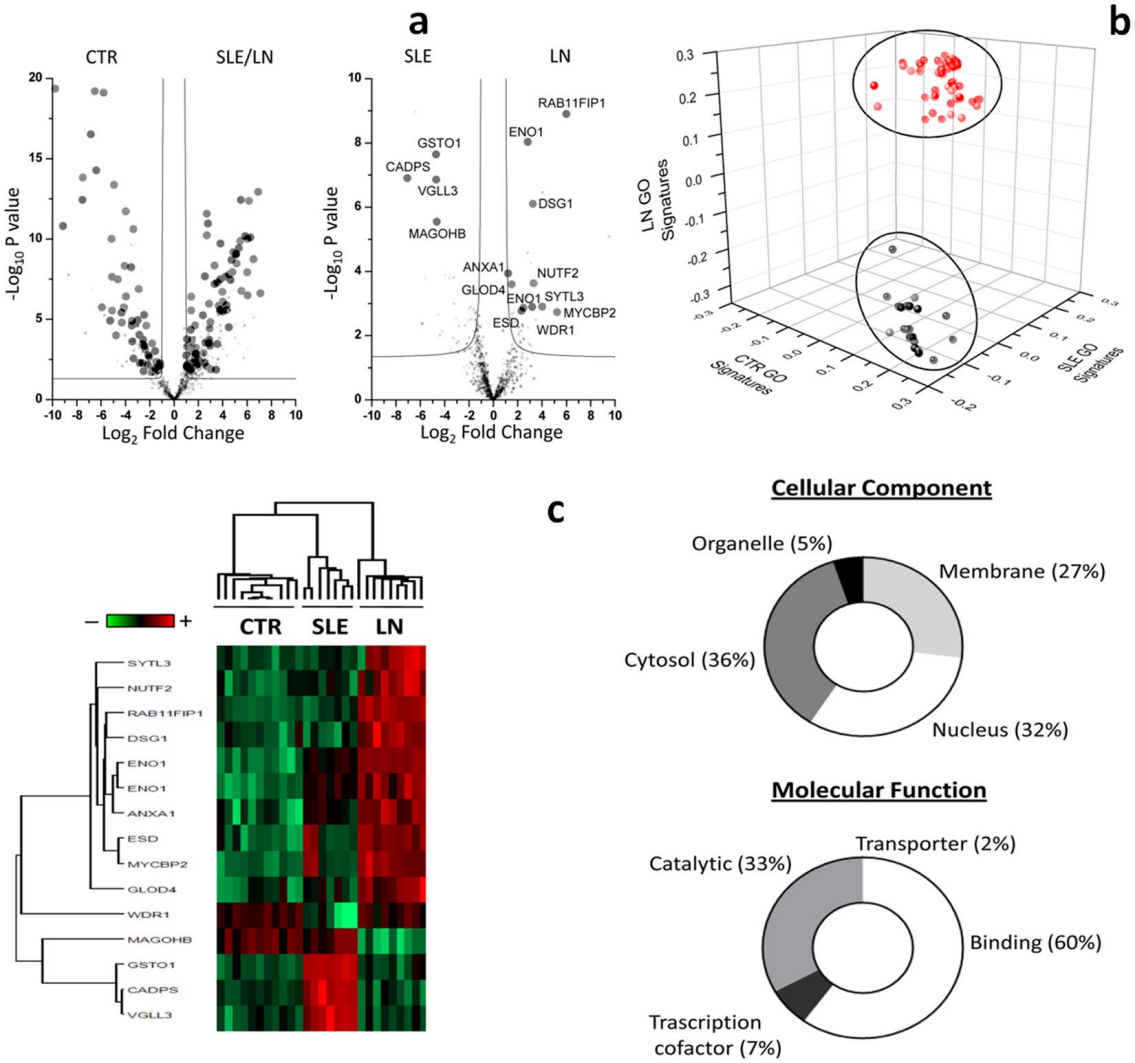
(**a**) Volcano plot based on fold change (Log_2_) and P value (−Log_10_) of all NET proteins identified by the comparison of control and SLE (with/without nephritis) supernatants and **b**) between SLE and LN neutrophils. Darkgrey circles represent the proteins highlighted by the combine use of univariate statistical analysis, PLS-DA and SVM: on the right, proteins more expressed in NETs from LN (n11), on the left proteins more expressed in NETs from SLE (n4). (**b**) Three-dimensional scatter plot of gene ontology signatures. The three-dimensional scatter plot of gene ontology (GO) analysis shows two distinct cluster of GO signature. Ellipses show the area at 95% of CI. In Red the GO signatures of SLE and LN and in black those of CTR. (**c**) Heat map and GO annotation Pie-chart of highlight proteins. Heat map of proteome profile of NETs proteins highlighted by the comparison of LN and SLE NETs. In heat map each row represents a protein and each column corresponds to one sample. Normalized Z-score of proteins abundance are depicted by a pseudocolor scale with red indicating positive expression, white equal expression, and blue negative expression compared to each proteins values whereas the tree dendrogram displays the results of a unsupervised hierarchical clustering analysis placing similar proteome profile values near each other. Visual inspection of the dendrogram and heat map shows a clear difference in protein expression of NETs deriving from different patients groups (control, SLE and LN). Pie-chart of Gene Ontology classification for cellular components and molecular function. The plot shows that most of proteins are associated to cytosol or nucleus of neutrophils; for molecular function, most proteins are involved in the binding or catalytic activity.

A total of 697 proteins were identified in PMA stimulated neutrophils (Supplement Table S1). Among these, 404 (57,9%) proteins overlapped among the 3 different groups of interest and only 53 (7.6%), 18 (2.6%) and 222 (31.9%) were exclusive for LN, SLE and Control netrophils respectively (Fig. 1a). The majority of the 697 NET components corresponded to proteins associated with autoimmune disease (477), some of them had been previously described in relation with SLE (169); 140 were specific of neutrophils (UniProt, Open Target and Atlas database). Sub-cellular localization was similar: 28–30% of proteins were localized in membranes, 32–34% in cytoplasm/cytoskeleton, 15% in organelle and 23–24% in nucleus (Fig. 1a). MDS analysis allowed to distinguish 3 clusters relative to the 3 different clinical conditions with marginal overlap of areas at 95% of confidence interval (CI) between SLE and LN (Fig. 1b). Volcano plot reports proteins with at least a two-fold increment and P-values ≤ 0.05 (after correction for multiple interactions) as limit for significance (Fig. 2a). The first Volcano considered SLE and LN together as compared with control neutrophils: a total of 137 proteins were differently expressed by the two cohorts (Supplement Table S2). Among these, 56 and 81 proteins were enriched respectively in Control and SLE/LN groups. The proteome profile that includes the differences above is highlighted by the heat map after Z-score shown in Supplement Fig. S1a. These 137 proteins were classified on the basis of their cellular components (CC) and molecular function (MF) according the available GO signatures: 35% of proteins were annotated as cytosol/cytoskeleton, 31% as nuclear, 28% as organelle and 6% as membrane proteins. Based on their molecular function, 40% proteins were classified as binding proteins, 39% as protein with catalytic activity, 12% as structural molecules, 4% as proteins with antioxidant activity, 1% as signal transducers and 2% as transporters and receptors (Supplement Fig. S1b). Signatures for Controls, SLE or LN derived from all identified proteins and their relative abundance are showed in Fig. 2b. In this diagram reports the significant GO signatures of Control (x-axis), LN (y-axis) and SLE (z-axis). Non-hierarchical cluster analysis with K-means of this plot shows two distinct clusters characterized by the GO enriched in Control (black circles) or in SLE and LN (red circles). The ellipse shows the cluster area at 95% of confidence interval (CI).

#### SLE vs LN

The same statistical approach was adopted to characterise the differences of NETs composition in supernatants of SLE or LN patient-derived neutrophils (Supplement Table S2): 15 proteins were found to discriminate between the two conditions and are reported in details in Table 1 and in Fig. 2a. Among these, 4 and 11 proteins were over-expressed in SLE and LN supernatants respectively. The differences above are shown by the heat map after Z-score in Fig. 2c. The 15 proteins above were classified on the basis of CC and MF according the available GO reference signatures: 36% of proteins were annotated as cytosol, 32% as nuclear, 27% as membrane and 5% as organelle components. Based on MF, 50% proteins were classified as binding proteins, 33% as catalytic activity, 7% as transcription factor and 2% as transporter (Fig. 2c).

**Table 1:** List of the fifteen proteins what maximize the discrimination between SLE and LN NETs samples.

Protein IDs	Protein name	Gene name	Fold Change SLE/LN vs CTR	−Log_10_ P-value SLE/LN vs CTR	Fold Change LN vs SLE	−Log_10_ P-value LN vs SLE
H7C571	Transcription cofactor vestigial-like protein 3	VGLL3	2.199		−4.698	6.863
F5H6P7	Protein mago nashi homolog	MAGOHB	−2.589	2.279	−4.649	5.547
F6TLX2	Glyoxalase domain-containing protein 4	GLOD4	1.386		1.496	3.599
H7C3U4	E3 ubiquitin-protein ligase MYCBP2	MYCBP2	6.549		5.216	2.736
O75083	WD repeat-containing protein 1	WDR1	−1.464		3.179	2.897
P04083	Annexin A1	ANXA1	1.402	5.546	1.2	3.938
P06733	Alpha-enolase	ENO1	5.149	8.477	2.476	2.873
P06733-2	Alpha-enolase; MBP-1	ENO1	5.074	9.022	2.829	8.035
P10768	S-formylglutathione hydrolase	ESD	2.824		2.307	2.775
P61970	Nuclear transport factor 2	NUTF2	2.661		3.303	3.631
P78417	Glutathione S-transferase omega-1	GSTO1	2.586		−4.711	7.646
Q02413	Desmoglein-1	DSG1	1.507		3.239	6.108
Q4VX76-2	Synaptotagmin-like protein 3	SYTL3	2.797		4.011	2.901
Q6WKZ4-3	Rab11 family-interacting protein 1	RAB11FIP1	3.928		5.995	8.906
Q9ULU8	Calcium-dependent secretion activator 1	CADPS	2.449		−7.085	6.906

#### Biostatistical analysis

**Figure 3:**
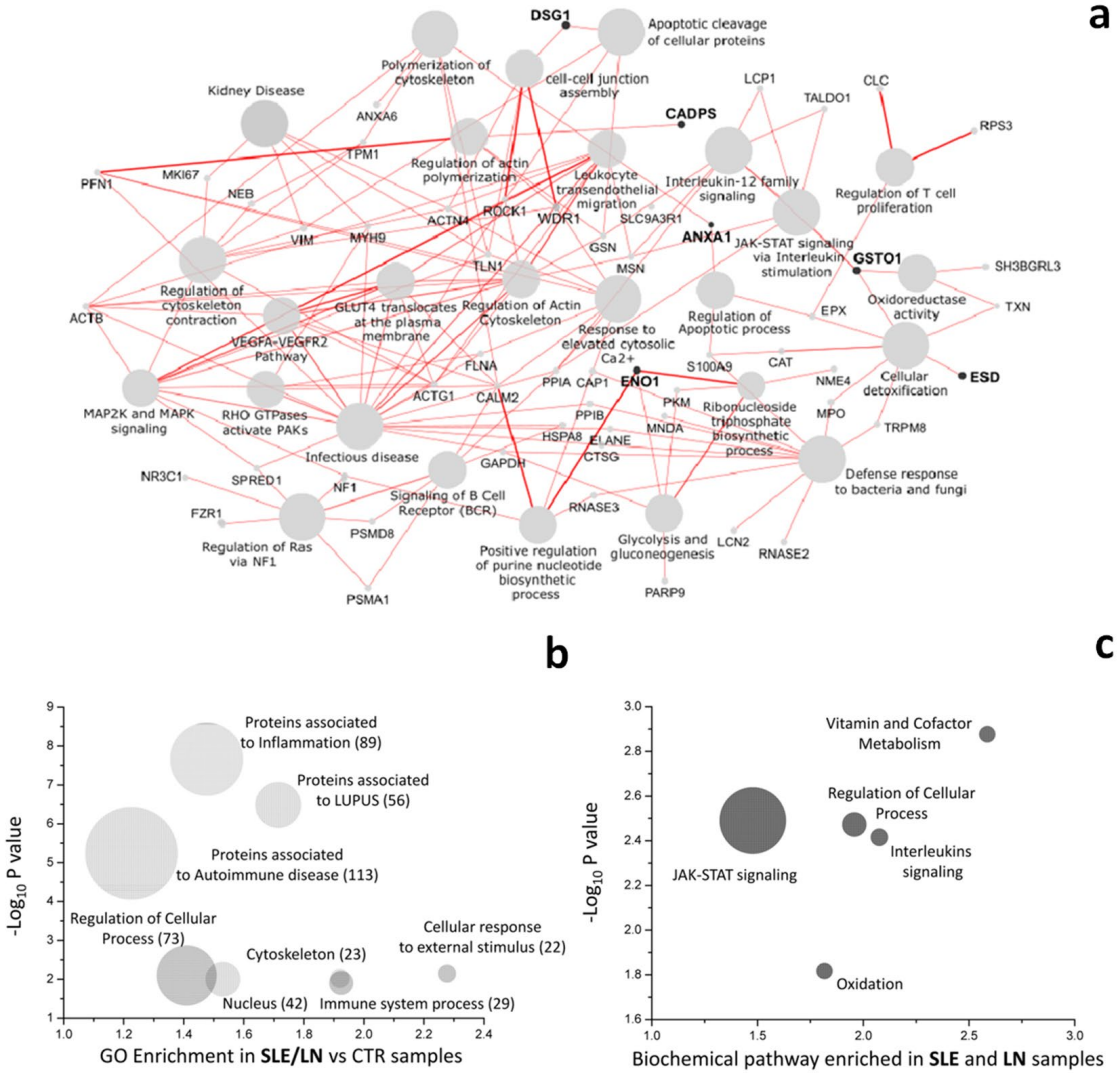
(**a**) Interaction networks of proteins/peptides of SLE and LN NETs. The diagram of proteins interaction networks (PPI) shows the interaction of the SLE and LN proteins/peptides highlighted by the combine use of uni-multivariate statistical analysis and learning machine and the relative biochemical pathways. The circle size of biochemical pathway is proportional to the number of proteins associated and the thickness of edge is proportional to the strength of interaction. Proteins PPI is analysed by Cytoscape software with ClueGo app. (**b**) GO Enrichment of NETs proteins of SLE and LN. Two-dimensional scatter plot analysis of mean enrichment factor and P value (−Log10) (Fisher exact test) of the proteins associated to SLE and LN compared to CTR samples. The size of circle are proportional of the number of associated proteins to each GO signature. (**c**) GO Enrichment of biologically relevant pathways in NETs from SLE and LN. GO analysis highlights biochemical pathways in which proteins expressed in SLE and LN participate. Graph show the mean of Log2 fold change of protein associated to each pathway (x-axis) and their P value (−Log10) by the comparison with CTR. The size of circles is proportional of the number of associated proteins. These pathway are also highlighted by the comparison of SLE and LN (Reactome database).

Interactions of proteins/peptides over expressed by LN and SLE NETs is presented in Fig. 3a–c. Four proteins over-expressed in NETs of LN (ie ENO1, ANXA1, DSG1 and ESD) are hubs for cellular and extra-cellular functions. In particular ENO1 is involved in the energetic metabolism (glycolysis and gluconeogenesis with GAPDH, PKD and PARP9), has a function in the transport of hexoses (traslocation of GLUT4 in membrane CALM2 and ACTG1), in biosynthesis of nucleotides (MNE4, RNASE3, CLAM2 and NF1), in the response to infections (GSTO1, SH3BGRL3, TXN, CAT, ESD, TRPM8, RNASE2, LCN2, GAPDH, CTSG, ELANE, MNDA, MPO, CAP1, SPRED1, ACTB, PFN1, MYH9, ROCK1, ACTG1, CALM2, HSPA8, ELANE, GAPDH) and, finally, it is involved in cytoskeleton and cell motility (ACTG1, ACTB, VIM, MYH9, ACTN4, TLN1, TPM1, ANXA6, ANXA1, CALM2, DSG1, ROCK1, CADPS, GSN and MSN). ANXA1 is connected with regulation of cytoskeleton, in regulation of apoptosis and in interleukin signaling. DSG1 is directly linked with the cleavage of apoptotic proteins and via ROCK1 with MAPK signaling and infectious diseases (WDR1). ESD is directly linked with detoxification processes, and indirectly (MPO,TRPM8) with defence against bacteria and fungi. To describe more in general the interconnections, we drew two ‘bubble diagrams’ of significant top GO signatures highlighted by previous analysis (Fig. 3b,c). Interestingly, many proteins were enriched in biochemical processes associated with inflammation, autoimmunity and lupus diseases and the major biochemical pathways were implicated in regulation of cellular process, interleukins and JAK-STAT signaling, vitamin and metabolic cofactors and oxidation.

### Post-translational modifications of NETs components

**Figure 4:**
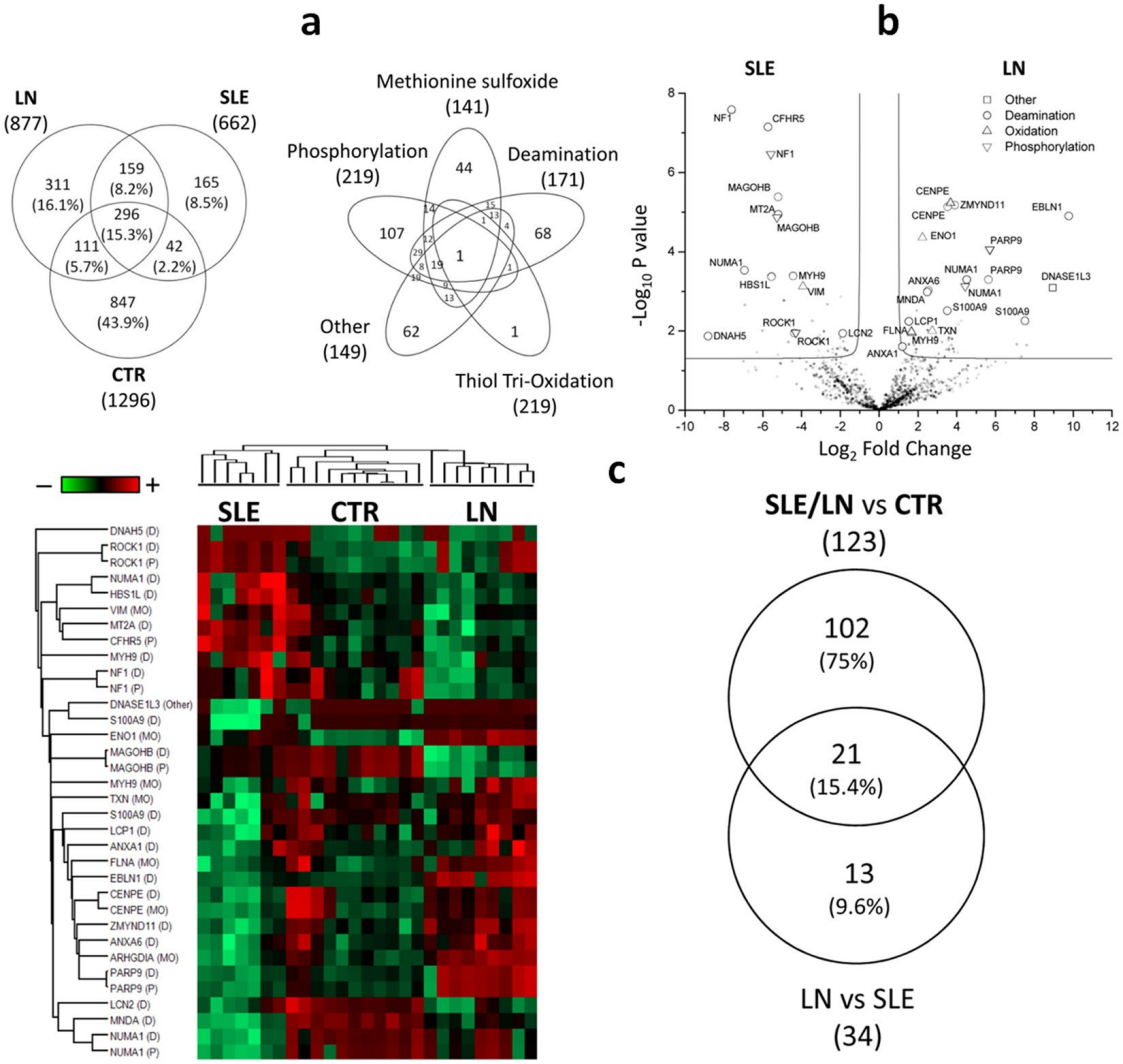
(**a**) Venn diagram of modified peptides and proteins that characterized different NETs. Venn diagram of NETs peptides with at least one post translational modification and type of modification identified by means of mass spectrometry in NETs deriving from LN, SLE and control cells. Numbers, represent the distinct peptide/protein in the respective overlapping and not-overlapping areas. The overlaps among different modifications indicate that each protein is modified following more than one mechanism. (**b**) Volcano plot of of NET peptides. Volcano plot based on fold change (Log_2_) and P value (−Log_10_) of peptides with at least one post-translational modification identified in NETs produced by SLE and LN neutrophils. Peptides above the line and identified as open triangle, circles and square represent those discriminant with statistically significant changes: on the right, peptides more expressed in NETs from LN, on the left peptides more expressed in NETs from SLE. (**c**) Heat map of NET peptides. Heat map of the peptide profile that characterized NETs produced by SLE, LN and control cells. Heat map utilizes univariate analysis, support vector machine, and partial least square discriminant analysis. Each row represents a peptide and each column corresponds to one sample. Normalized Z-score of peptides abundance are depicted by a pseudocolor scale with red indicating positive expression, white equal expression, and blue negative expression compared to each peptides values, whereas the tree dendrogram displays the results of a unsupervised hierarchical clustering analysis placing similar peptide profile values near each other. Venn diagram of significant NET peptides with at least one post trasductional modification identified by the comparison of CTR vs SLE with/without nephrites (one circle) and SLE vs LN (second circle). Venn diagram shows common and exclusive peptides. Numbers represent the distinct peptides in the respective overlapping and not-overlapping areas. 21 peptides allow to distinguish simultaneously Control, SLE and LN NETs.

Results of the characterization of post-translational modifications (PTM) of the NET peptides are presented in Fig. 4a. We evaluated all types of PTM, grouping them in: methionine (sulfoxide), thiol oxidation (sulfonic acid), deamination of N, Q and K aminoacids (citrullination) and all other types of PTM.

A total of 1931 peptides with at least one PTM were identified (Fig. 4a) corresponding to 440 proteins. The majority of PTM was found on Control peptides (1296) followed by LN (877) and SLE (662). Only a few of these PTM were exclusive for LN (16.1%) and SLE (8.5%) samples or(8.2%) were in common with the two. Despite some overlapping, SLE and LN showed a peculiarity of PTM peptide composition and profile intensity that allowed to clear distinguish these samples from control NETs (Supplement Fig. S2). To better describe these differences we utilized univariate statistical analysis, PLS-DA and SVM. A total of 123 peptides with at least one PTM were found when Control cells was compared to SLE and LN as considered together (Supplement Fig. S3a). The profile of these highlighted PTM peptides were showed by means of heat map after Z-score (Supplement Fig. S3b).

The same analysis was performed to describe the differences between SLE and LN. A total of 34 peptides were highlighted by volcano plot (Fig. 4b). The profile of these highlighted peptides were shown by means of heat map after Z-score (Fig. 4c left side): among this, 21 PTM peptides identify a core of shared peptides, when Control cells were compared to SLE and LN together and SLE was compared to LN (Fig. 4c right side). This core panel of peptides allows to distinguish simultaneously CTR, SLE and LN NETs samples (Supplement Table S3) and the corresponding proteins are in relationship with autoimmune and SLE disease (Uniprot, Disgenet, Open Target and Atlas database).

### Oxidised αenolase in NETs discriminates LN vs SLE and controls

**Figure 5:**
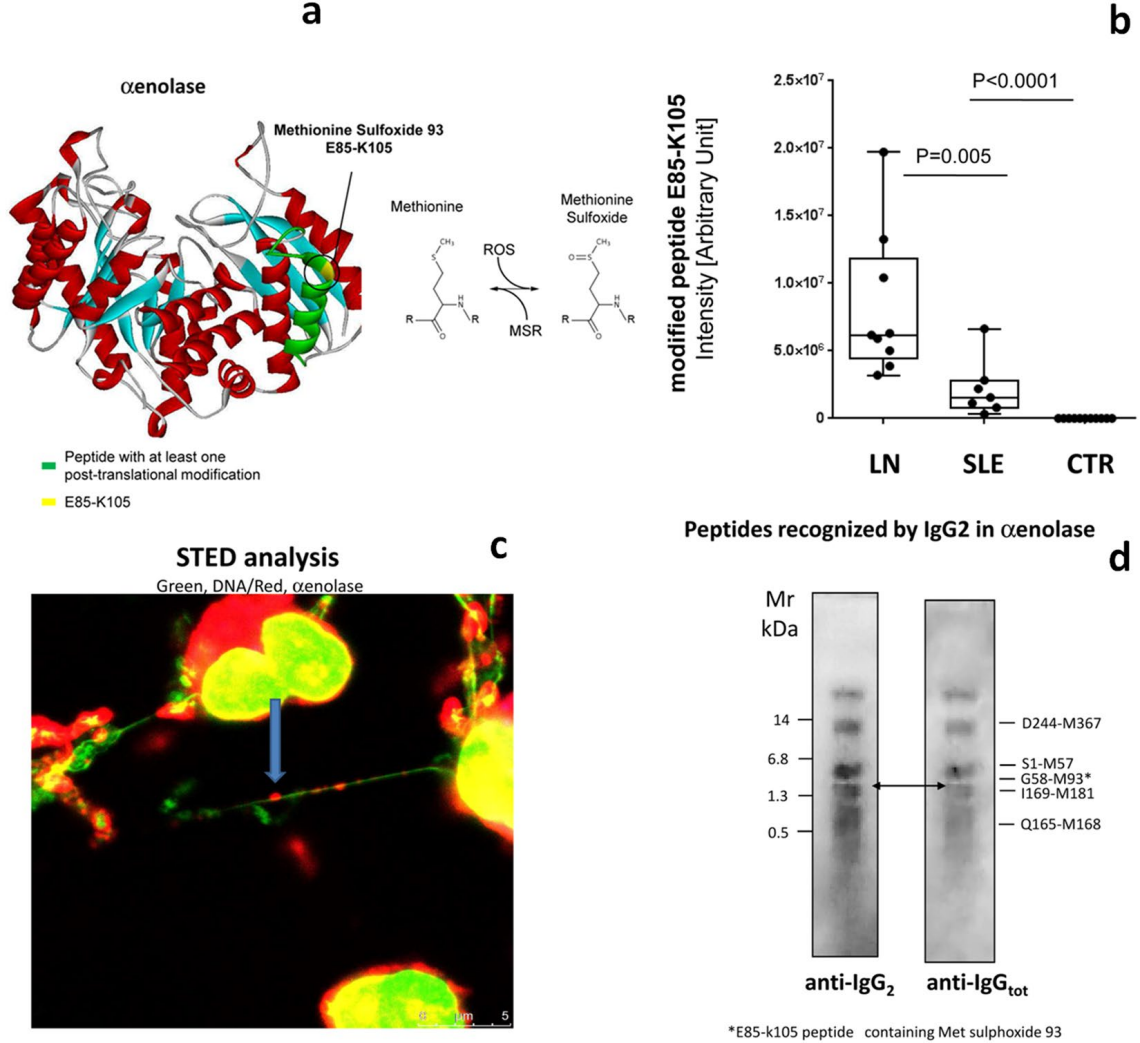
Oxidised αenolase: characterization, expression and localization. (**a**) Post-translational modifications of αenolase in NETs were characterized by Fusion Orbitrap; the E85-K105 peptide containing methy-sulfoxide methionine 93 was found in NETs limited to LN patients; three-dimensional structure of αenolase where the epitopes for IgG2 interaction (see below) are reported in green and the merge between G58-M93 and E85-K105 is shown in yellow-red. (**b**) Intensity of the E85-K105 peptide containing methy-sulfoxide methionine 93 in NETs from LN and SLE. (**c**) Stimulated emission depletion microscopy (STED) analysis of filaments. (**d**) The epitopes of αenolase that are recognized by anti-αenolase IgG2 purified from serum of LN patients. The protein was digested by CNbr and peptides deriving from digestion were separated by western-blot.

LN patient-derived NETs were characterized by a high expression of αenolase (ENO1, 2 isoforms: P06733 and P06733-2) (see Table 1). PTM relative to αenolase and characterized by Fusion Orbitrap in supernatant of NETs are outlined in Fig. 5a. A major PTM that characterized LN NETs was methionine sulfoxide in place of methionine 93 that is included in the E85-K105 peptide. The expression of the modified E85-K105 containing methionine sulphoxide was significantly increased in LN patients vs SLE; all controls had normal methionine 93 with null expression of the modified peptide (Fig. 5b). The localization of αenolase in the NET filaments was typically outlying DNA (Fig. 5c).

Based on this finding we finally sought to characterize the specific epitopes on αenolase recognized by antibodies and in particular by IgG2 that is the isotype of circulating anti-αenolase autoantibodies (see below). Six peptides of decreasing MW from 14 to 0.5 KDa generated by CNBr clevage of αenolase (Fig. 5a) were recognized. Noteworthy, the peptide containing the methionine sulfoxide in place of methionines 93, corresponding to CNBr fragment G58-M93, was recognized by circulating autoantibodies (Fig. 5d). That findings support the recognition by immune system of αenolase post-translational modified epitopes.

### Circulating anti-αenolase auto-antibodies in patients with LN and in Membranous nephropathy

**Figure 6:**
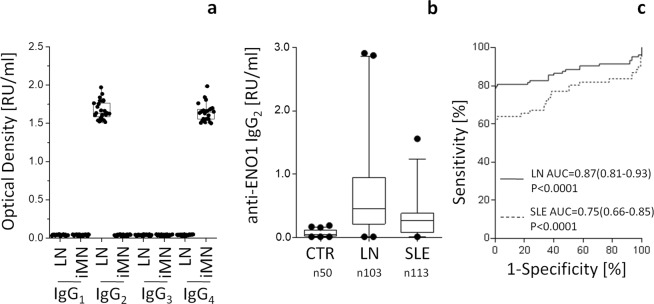
Isotype-specificity of anti-αenolase antibodies and circulating levels in different autoimmune-conditions. (**a**) Isotype specificity of anti-αenolase antibodies was evaluated in 20 patients with Lupus Nephritis (LN) and in 20 with idiopathic Membranous Nephropathy by western-blot. (**b**) Circulating levels of anti-αenolase IgG2 were determined in serum of patients with SLE (n113), in LN (n103) and in 20 with MN. The former groups had been recruited in the frame of the Zeus study https://clinicaltrials.gov (study number: NCT02403115). A home-made ELISA was utilized for these determinations^42^. (**c**) Sensitivity and specificity of anti-αenolase IgG2 in LN and in SLE patients were very high.

To further strengthen the relevance of αenolase antibodies in patients with LN, serum levels of autoantibodies against αenolase were assessed in a large cohort of 103/116 patients having LN/SLE. In a preliminary approach, isotype specificity of anti-αenolase antibodies was tested in 20 patients with LN and in 20 with Membranous Nephropathy (MN) a well characterized condition of idiopathic autoimmune glomerulonephritis: anti-αenolase IgG2 were uniquely found in LN while IgG4 were specific for anti-αenolase in MN patients (Fig. 6a) therefore clearly showing a high isotype specificity of these antibodies in different autoimmune conditions. According to the above finding, anti-αenolase IgG2 levels were increased in serum of LN patients (Fig. 6b) and to a minor extent in SLE patients compared to control sera. Sensitivity and specificity were very high in both cases (Fig. 6c).

## Discussion

This study sought to characterize the protein composition and post-translational modifications of NETs produced *ex vivo* by resting and PMA-stimulated neutrophils isolated from blood of healthy donors, patients with SLE or with LN. Results reveal a complex composition of post-translational modified NET proteins and suggest their relevance in autoimmunity. PMA, a substance that stimulates NAPH-oxidase activity and increases oxygen radical production^10–12^, is credited as the *in vitro* model of NETs activation^10^. We utilized PMA to maximize the production of NETs and the differences between the studied conditions. Our data cast a new light on NET composition in different clinical settings extend the knowledge beyond DNA and histones and potentially provide a springboard for further mechanistic studies in autoimmune conditions such as SLE and vasculitis.

NETs include overall almost 700 proteins, 50% of the total corresponding to proteins already described in association with autoimmunity, inflammation and SLE. Moreover, it is here shown that neutrophils respond to an oxidative stimulus (PMA) by producing components that could be immunogenic: the 90% are membrane, cytosol and cytoskeleton proteins and present phosphorylation, methyl/thiol oxidation and oxidative deamination as major post-translational modifications. Our study, therefore, vastly extends the number of proteins present in NETs from the original description of 25 reported by Urban *et al*.^16^; this evolution was allowed by the use of high-throughput Fusion Orbitrap that is a new technology of analysis with much enhanced sensitivity in respect to previous mass spectrometry approaches.

This new finding on NETs composition is of particular interest since recent observations indicate that circulating NETs are increased in patients with LN nephritis and that their levels correlate with complement consumption and with levels of other classic antibodies and biomarkers of SLE (*i.e*., anti-DNA, etc)^26,28^. Increased NETs levels in LN, prompted us to investigate NET post-translational modified proteins generated by neutrophils in subsets of patients with SLE since it was hypothesized that they could represent potential auto-antigens recognized *de novo* by the immune system. As the results demonstrate, there are, in fact, NET associated proteins that are highly specific for LN (n = 11) and for SLE (n = 4); in addition to specific NET-associated proteins, we also observed post-translational modifications specific for SLE and LN including oxidative changes and new deamination residues.

Among the most expressed NET-proteins that were produced by neutrophils deriving from LN patients there were two, i.e. Annexin A1 and αenolase, that have a recognized regulatory role within the immune system and were, for this reason, further characterized. Annexin A1 (AnxA1) is a 37 KDa protein with phospholipid–binding properties that is expressed in cytoplasm of nucleate cells of blood^29,30^; it has multifunctional roles in innate and adaptive immunity mainly in the control and resolution of inflammation^31,32^. Annexin A1 levels are regulated by glucocorticoid and play many of their anti-inflammatory effects^33^; it also modulates neutrophil apoptosis and promotes their phagocitosis by macrophages^34^. Increasing evidence indicates that Annexin A1 plays anti-inflammatory effects in Rheumatoid Arthritis^35^ and promotes breast cancer progression and metastasis^36^. Anti-Annexin A1 antibodies have been detected in association with SLE and Rheumatoid Arthritis^37^ and have been proposed as diagnostic markers of discoid lupus^38,39^. Alpha-enolase is a glycolytic enzyme with multiple localizations and functional implications that go behind the metabolic role^40^. In eukaryotes, αenolase is expressed manly in cytoplasm of cells but it is also present in the outer membrane of several epithelial, endothelial and hematopoietic cells^41^ where it acts as plasminogen receptor and activator. Circulating anti-αenolase antibodies have been described in association with LN^42–44^ and in other autoimmune conditions having the kidney as principal target such as idiopathic membranous Nephropathy^45^. Based on a potential connection between LN and NETs, αenolase became a main focus of our study. A first finding was that in NETs produced by LN cells, αenolase is modified for the presence of sulphoxide methionine 93, that is constant in all LN patients compared to few SLE (with a highly statistical difference). We hypothesized that oxidation of αenolase in NETs contributes to break tolerance and leads to the formation of anti-αenolase antibodies. It is, in fact, currently accepted that NET formation, beside determining externalization of nucleosome and DNA, produces post-translational modifications in other nucleosome components^20,25,27,46,47^ inducing the *de novo* formation of potential auto-antigens. Evidence of NETs as a source of auto-antigens has been documented in Small Vessels Vasculitis where concomitant increased of NET production is associated with the presence of ANCA-associated autoantibodies against MPO and proteinase 3 that are two components of NETs. Therefore, modified proteins in NETs should be considered as trigger of autoimmunity in terms of increased production of autoantigens.

Our main focus was LN, a severe complication of SLE developing in almost 50% of patients and leading to renal failure (ESRD)^48^. In fact, though anti-DNA antibodies are considered the main culprit for disease onset in SLE patients, other autoantibodies have also been detected in the kidney of those patients who develop LN and anti-αenolase represent a major nephritogenic auto-antibodies in this condition^44^.

In the validation study we focused some aspects related to the specificity of anti-αenolase antibodies in LN in comparison with other autoimmune conditions targeting the kidney and results add something to elucidate the mechanisms leading to their formation. In fact, circulating anti-αenolase antibodies have been also described in MN, an idiopathic autoimmune conditions linked with the presence in serum of circulating auto-antibodies versus several glomerular basement membrane proteins^49^ here including anti-αenolase^45,50^. We observed a rigid isotype specificity of anti-αenolase antibodies in LN versus MN, since IgG2 were the unique antibodies in LN and IgG4 in MN. High serum levels of anti-αenolase IgG2 in patients with LN suggest that the adaptive immune response leading to their formation involves TLR8 and TLR9 that are the TLRs driving an IgG2 isotype switching. Experiments are now in course to demonstrate a direct effect of oxidised αenolase deriving from NETs in stimulation of TLRs 8/9 and in anti-αenolase IgG2 production. Two ancillary observations presented here indirectly support this possibility: one is the localization of αenolase contiguous and external to DNA in NET filaments suggesting it is of easy accessibility to TLRs and B cells; the second is that IgG2 purified from sera of LN patients interact with the epitope G58-M93 that overlaps with the site of αenolase oxidation (*i.e*. sulphoxide methionine 93). It must be stressed here that, in spite generation of anti-αenolase IgG2 should be linked to oxidized αenolase and TLR8/9 stimulation, anti-αenolase antibodies react also with non oxidized αenolase as in our ELISA assay, that utilizes the unmodified protein.

## Conclusions

This study reveals, for the first time, the vast complexity of the protein composition of Neutrophil Extracellular traps produced ‘*ex vivo*’ by cells purified from patients with SLE and LN compared to cells extracted from healthy donors. NETs protein composition is highly specific for the different clinical settings and methyl-oxidized αenolase (with sulphoxide methionine 93) is highly characteristic for NET of LN vs SLE patients and absent in controls. We also showed that sulphoxide methionine 93 is included in the peptide recognized by specific IgG2 that are increased in sera of LN patients and represent autoantibodies highly characteristic of this pathology. Overall, data here presented support the idea that modification of NET αenolase is the starting mechanism leading to the formation of circulating auto-antibodies specific for LN that deposit within glomeruli and determine lupus nephritis, the most serious complication of lupus erythematosus.

## Materials and Methods

### Study design

*‘Ex vivo’* cell NETs production studies were performed in 9 LN, 7 SLE and 11 normal subjects. All the patients of the present study were out of therapy at the time of blood collection.

Diagnosis of SLE was done according to the American College Rheumatology criteria (SLICC) as outlined with details in the Validation study (see below). Lupus nephritis was defined according to WHO classification (see below). We obtained written approval of the protocol by the local Independent Ethics Committee (Comitato Etico Regione Liguria) on October 24, 2014. All methods were performed in accordance with the relevant guidelines and regulations.

### Methods

#### Isolation of neutrophils

Polymorphonuclear cells (PMN) were isolated from -EDTA peripheral blood under sterile conditions using a dextran sedimentation technique followed by Ficoll gradient centrifugation as previously described^51^. Briefly, 1 volume EDTA blood was mixed with 0.8 volume of Dextran Plander 70000 solution (Fresenius Kabi Italia s.r.l, IsoladellaScala, VR, Italy) and RBC were allowed to sediment for 45 minutes at room temperature. The granulocyte-rich supernatant was then collected, layered onto Ficoll-Histopaque 1077 and centrifuged at 800 g for 30 minutes. Residual RBC were removed from granulocyte- containing pellet by hypotonic lysis. PMNs were finally re-suspended in RPMI medium supplemented with 1 mM Calcium Chloride and 1% Human Serum Albumin solution (Albital 200 g/l, Kedron, CastelvecchioPascoli, Borgo, LU, Italy) for further investigations.

#### ‘*In vitro*’ NETs induction

PMN suspensions were allowed to adhere for 1 hour at 37 °C onto 24-well plastic dishes at the density of 10 × 10^6^ cells/ml in RPMI medium, supplemented with Calcium Chloride and Human Serum Albumin as above described. NET formation was thereafter induced by treating PMNs for 3 hours with 20 nM Phorbol myristate acetate (PMA). Cells were then washed with PBS and incubated with 15U/ml S7 Nuclease for 15 minutes at 37 °C. Reaction was stopped with 2 mM EDTA. Cellular debris were then pelleted by centrifugation at 300 g and supernatants saved for Elastase assay (see below).

#### Determination of NETs production

To quantify NETs production, it was used the Cayman’s NETosis assay kit (cat. No 601010, Cayman Chemical, MI, USA) according to the manufacturer’s instructions. Briefly, 100 ul of standard or culture supernatants per well, pre-heated to 37 °C, were incubated with 100 ul per well of the 1:30 diluted NET assay neutrophil elastase substrate in PBS. The 96-well plate was cover and incubated 2 hours at 37 °C. Finally, the cover was removed and the plate was read at 405 nm.

#### Sample preparation for Mass Spectrometry, NanoLC and Mass Spectrometer setup

Pellets obtained by acetone precipitation were re-suspended in 25 µl of lysis buffer (6 M GdmCl, 10 mM TCEP, 40 mM CAA, 100mMTris pH8.5). The samples were reduced and alkylated and lastly digested in a single step and then loaded into StageTip. Peptides were analyzed by nano-UHPLC-MS/MS using an Ultimate 3000 RSLC with EASY spray column (75 μm × 500 mm, 2 μm particle size, Thermo Scientific) and with a 180 minute non-linear gradient of 5–45% solution B (80% CAN and 20% H_2_O, 5% DMSO, 0.1% FA)at a flow rate of 250 nl/min. Eluting peptides were analyzed using an Orbitrap Fusion Tribrid mass spectrometer (Thermo Scientific Instruments, Bremen, Germany). Orbitrap detection was used for both MS1 and MS2 measurements at resolving powers of 120 K and 30 K (at m/z 200), respectively. Data dependent MS/MS analysis was performed in top speed mode with a 2 sec. cycle-time, during which precursors detected within the range of m/z 375−1500 were selected for activation in order of abundance. Quadrupole isolation with a 1.4 m/z isolation window was used, and dynamic exclusion was enabled for 45 s. Automatic gain control targets were 2.5 × 10E5 for MS1 and 5 × 10E4 for MS2, with 50 and 60 ms maximum injection times, respectively. The signal intensity threshold for MS2 was 1 × 10E4. HCD was performed using 30% normalized collision energy. One microscan was used for both MS1 and MS2 events. For all the MS1 scans the option ETD internal Calibration was selected.

MaxQuant software, version 1.5.5.30, was used to process the raw data, setting a false discovery rate (FDR) of 0.01 for the identification of proteins, peptides and PSM (peptide-spectrum match), moreover a minimum length of 6 amino acids for peptide identification was required. Andromeda engine, incorporated into MaxQuant software, was used to search MS/MS spectra against Uniprot human database (release UP000005640_9606 February 2016).Two different elaborations were made to identify the PTMs in order to limit the false positives. In the first processing, variable modifications are Acetyl (Protein N-Term) and Phospho (STY). The second processing step includes Oxidation (M), Deamidation (NQ), Trioxidation (C) and Carbamido-methyl (C). Finally, in order to overcome the common limitations of search engine based PTM analysis, we used the unbiased PTM Dependent Peptide search option, taking advantage of high mass accuracy data collected in high resolution mode with an internal calibration (MS1 error < 1 ppm). The intensity values were extracted and statistically evaluated using the different Site Table, DP table or Protein Groups table. Algorithm MaxLFQ was chosen for the protein quantification with the activated option ‘match between runs’to reduce the number of the missing proteins.

MS proteomics RAW data, Peptides and Proteins Table are available at the ProteomeXchange Consortium database via the Proteomics Identifications (PRIDE) partner repository, under data set IDs PXD007754 Reviewer account details:

Username: reviewer49884@ebi.ac.uk

Password: RmJZrfxD

#### Purification of serum αenolase and CNBr fragmentation

The experiments for defining the epitopes of αenolase recognized by anti-αenolase IgG2 were done with the protein purified from sera of 5 LN patients. Fifty ml of pooled serum were overall processed. Anti-αenolase monoclonal antibody (Abcam) were immobilized on 50 ml ProteinA Sepharose (Bio-Rad) and washed with PBS. Sera were loaded and re-circulated overnight at 4 °C and after several washes with the PBS buffer, bound substances were eluted in Glycine-HCL buffer pH2,5. The eluate was utilized for CNBr digestion according to the conditions below: 1-purified αenolase in 0.4 M ammonium bicarbonate was incubated with 1% v/v 2-mercaptoethanol at room temperature for 1 h in a dark box; 2-the sample was dried in speed vacuum and re-suspended in 5 μL of deionized water, 15 μL of trifluoroacetic acid (TFA) and 5 μL of 5 M CNBr in acetonitrile (ACN). The tube was wrapped in aluminum foil and left overnight at 4 °C; 3-the reaction was stopped by drying down under vacuum; 4-finally, the sample was re-suspended in Tris-HCl 62,5 mM pH 6,8, 2% w/v SDS and 10% glycerol, loaded in polyacrylamide gel and transferred on PVDF membrane. After saturation the membrane was incubated with a pooled sera of LN patients, diluted 1:50 in PBS-T and 1%w/v BSA, rinsed with PBS-T and then incubated with anti-human IgG HRP-conjugated diluted 1:3000 in PBS-T and 1%w/v BSA. Chemioluminescence was use for detection.

#### Stimulated emission depletion microscopy (STED)

Other images (1024 × 1024 pixels, 8 bit) were acquired with a super-resolution laser scanning microscope based on stimulated emission depletion method STED microscope Leica SP5 TCS STED-CW gated (Leica-microsystems, Mannheim, Germany) equipped with an oil immersion HCX PL APO 100× 1.4 NA objective set the pinhole to 1 Airy unit; time gating for the red channel was starting at 2 ns and ending at 10 ns. A series of confocal optical sections were taken at a z-step of 25 nm. The image acquisition through red (spectral window 514–553 nm) and green (spectral window 467–495 nm) channels were performed according to a time-sequential protocol to reject possible cross-talk artifacts. In particular, the excitation beams were at 514 nm (a pulsed, 80 MHz, super-continuumlaser) and 458 nm (a continuous wave, CWlaser) respectively. While the depletion beam was at 592 nm (CW laser) for both channels, the power was set at 120 mW and 350 mW respectively. The Leica Confocal Software program was used for image acquisition, storage and analysis. Illustrations were prepared using the freely available software ImageJ (rsb.info.nih.gov/ij), originally developed by NIH.

### Validation study

#### Sample size SLE/LN

Overall, 216 incident SLE patients were included in the study; 103 patients of the SLE patients presented a glomerulonephritis at the time of recruitment. All the patients above were recruited in the frame of the Zeus study^44^. The data base and samples collection is located at the Giannina Gaslini Institute of Genoa (I). None of the participants had a diagnosis before. Twenty-five healthy donors were recruited from the hospital staff (age 23–56 yrs). Patients were recruited prospectively and were out of therapy at the time of blood collection.

All gave their informed consent to the study protocol.

Inclusion Criteria were age between 4 and 65 years, any sex, the availability of informed consent. Diagnosis of systemic lupus erythematosus was done according to the American College Rheumatology systemic lupus classification criteria as revised by the Systemic Lupus International Collaborating Clinics (SLICC)^52^. Newly diagnosed lupus nephritis (stage I-VI according to WHO classification) were recruited among the large cohort of patients with lupus showing positivity of urinary elements such as hematuria, proteinuria and/or worsening of renal function in some cases as evaluated by the CPK-EPI formula. The diagnosis of LN was based on typical renal lesions as analyzed by immune-fluorescence and classical histology staining. For histology evaluation of kidney disease, Dubosq–Bresil solution-fixed tissues were embedded in paraffin, sectioned, and stained with hematoxylin/eosin, Masson’s trichrome, silver methenamine, and periodic–acid Schiff. Routine immunofluorescence studies on frozen sections were performed using anti-human IgG, IgA, IgM, C1q, C3, and fibrinogen antibody. Sera autoimmunity was evaluated utilizing commercial essays for ANA, nDNA, ENA. Exclusion Criteria were the presence of severe infections, malignancies, positivity for chronic hepatitis B Virus (HBV) or Hepatitis C Virus (HCV), breast-feeding or pregnant. Therapy manly consisted in steroids and hydroxylchloroqine in SLE and steroids plus cytotoxic drugs or cyclosporine in LN patients.

#### Membranous nephropathy

Twenty patients with Membranous Nephropathy were recruited within the Italian Consortium on Membranous Nephropathy that is chaired by Dr Ghiggeri at G. Gaslini Institute of Genoa. Criteria for enrollment were (1) a biopsy-based diagnosis of iMN, (2) a normal complement profile, (3) negative tests for ANA, nDNA, and ANCA and cryoglobulins and the absence of viral markers (hepatitis B surface antigen and HIV); (4) *the* absence, at the time of inclusion, of clinical and biochemical signs of cancer.

#### Ethical committee

Before initiation of the study, we obtained written approval of the protocol, Informed Consent Form and any information presented to potential subjects from the local Independent Ethics Committee (Comitato Etico Regione Liguria) on October 24, 2014. We also obtained approval from the Italian Drug Agency (Agenzia Italiana del Farmaco, AIFA). The study was registered at https://clinicaltrials.gov (study number: NCT02403115).

#### Isotype specificity

Isotype specificity of anti-αenolase antibodies in 20 sera of patients with LN and in 20 with MN was determined with Western blot as already described^42^.

#### Determination of anti-αenolase IgG2

A home made ELISA was utilized for determining serum levels of anti- αenolase IgG2 as already described^42^. Briefly, 100 ng of recombinant αenolase (Abnova Corporation, Taipei, Taiwan) were put in MaxiPrep plate 96 wells, in PBS buffer and incubate at room temperature for 5 hours and then at 4 °C overnight. Aliquots (200 μl) of blocking solution (PBS, 5% w/v BSA and 0.05% v/v Tween20) were put in each well. Serum samples (100 μl) diluted 1:50 in PBST (PBS – Tween20 0.05% v/v –BSA 1% w/v) was added and incubated for 4 hours at room temperature and then at 4 °C overnight. After three washes in PBST, HRP-conjugated rabbit anti human IgG2 (Clone: HP6014- InVitrogen Corporation, Camarillo, CA) diluted 1:3.000 in PBST and 1% w/v BSA were incubated at room temperature for 4 hours and after 3 washes in PBST, 100 μl of substrate TMB/H2O2 (10:1) was added and incubated up to 30 minutes. The reaction was stopped by adding 100 μl of 0.45 M of H2SO4 at any wells before reading absorbance at 450 nm. A standard curve was prepared utilizing HRP-IgG2 at different dilutions.

#### Normal limits

Normal limits for all the tests above were calculated from ROC curves; the Cut Off represented the value that minimizes the geometric distance from 100% sensitivity and 100% specificity on the ROC curves^53,54^.

### Standard confocal and Stimulated Emission Depletion Microscopy (STED)

Imaging techniques based on standard microscopy and STED are described in Supplementary Information.

### Statistics and bioinformatic analysis

After normalization, data obtained from mass spectrometry, were analyzed using unsupervised hierarchical clustering analyses, i.e. Multidimensional Scaling (MDS) to identify outlier and sample dissimilarity. Differences in protein and peptide expression between Control, SLE and LN NET cell supernatantss were analysed using a non-parametric U-Mann Whitney test. P-values were adjusted using the Benjamini-Hochberg method. Results were considered significant with two fold change and adjusted for P-value ≤ 0.05 (to identify the significant fold change a power analysis was performed considering the number of all biological replicates and their variability). Volcano plot was used to visualize the statistical differences, in which case the cutoff lines were established using the function y = c/(x − x0). Non-linear support vector machine (SVM) and partial least squares discriminant analysis (PLS-DA) were utilized to identify maximal discrimination among groups. In SVM a Cross-validation approach a 4-fold increment limit was applied to estimate the accuracy of classification. The results of these analysis were summarized by mean of heat map graph.

Differentially expressed proteins in NETs samples highlighted by the combine use of univariate/multivariate statistical analysis and machine learning were analyzed according to GO terms for biological process, cellular component and molecular function in the database (http://www.geneontology.org/). To assess functional associations between proteins, differentially expressed between CTR/SLE/LN, R software was applied and visualized with Cytoscape. Pathways enrichment of proteins clusters were performed according to UniProt, Reactome, KEGG and ClueGO database. The results of GO analysis were shown three and bi-dimensional scatter plots.

Test performance in terms of sensitivity (ability of the test to identify true positive subjects) and specificity (ability of the test to identify true negative subjects) was evaluated for each parameter by Receiving Operating Characteristic (ROC). The proportion of patients correctly diagnosed is proportional to the area under the curve (AUC) where accuracy is absent for AUC = 0.5, poor for 0.5 < AUC ≤ 0.7, moderate for 0.7 < AUC ≤ 0.9 and high for 0.9 < AUC < 1. A test is perfect for AUC = 1. The ROC curve also allows to identify the best cut off value that maximizes the difference between true positive subjects and false positives ones. To maximize sensitivity and specificity, the Youden’s index (Sensitivity + [1 − Specificity]) was applied. All statistical analysis were performed using software package R last version available at the time of experiments.

## Supplementary information

Supplementary Information
