# Supplementary material for: Neutrophil Extracellular Traps protein composition is specific for patients with Lupus nephritis and includes methyl-oxidized αenolase (methionine sulfoxide 93)
Source: Sci Rep. 2019 May 28;9:7934. doi: 10.1038/s41598-019-44379-w (PMC6538718; doi:10.1038/s41598-019-44379-w)
Supplement: Supplementary file 1 — Supplementary Information [file 41598_2019_44379_MOESM1_ESM.docx]

**Neutrophil Extracellular Traps protein composition is specific for patients with Lupus nephritis and includes methyl-oxidized αenolase (methionine sulfoxide 93).**

Maurizio Bruschi**^1^**, Andrea Petretto**^2^**, Laura Santucci^1^, Augusto Vaglio**^3^**, Federico Pratesi**^4^**, Paola Migliorini**^4^**, Roberta Bertelli**^1^**, Chiara Lavarello^2^, Martina Bartolucci^2^, Giovanni Candiano**^1^,** Marco Prunotto**^5^** and Gian Marco Ghiggeri^1, 6,*^

**^1^**Laboratory of Molecular Nephrology, IRCCS Istituto Giannina Gaslini, Genoa, Italy;

^2^Core Facilities-Proteomics Laboratory, IRCCS Istituto Giannina Gaslini, Genoa, Italy;

**^3^**Department of Biomedical Experimental and Clinical Sciences "Mario Serio", University of Firenze, and Meyer Children's Hospital, Firenze, Italy

**^4^**Department of Clinical and Experimental Medicine, Clinical Immunology Unit, University of Pisa, Pisa, Italy;

^5^School of Pharmaceutical Sciences, University of Geneva, Geneva, Switzerland;

^6^Division of Nephrology, Dialysis, and Transplantation, Scientific Institute for Research and Health Care, IRCCS Istituto Giannina Gaslini, Genoa, Italy;

**Corresponding Authors^*^**

Gian Marco Ghiggeri, MD, PhD,

Division of Nephrology, Dialysis and Transplantation, Laboratory Molecular Nephrology Science, Istituto G. Gaslini, Largo G. Gaslini 5, 16147, Genoa, Italy.

Phone: (+39) 010 380742. Fax: (+39) 010 395214.

E-mail: GMarcoGhiggeri@gaslini.org

**SUPPORTING INFORMATIONS**

**Suppllementary FIGURES**

**Figure S-1**


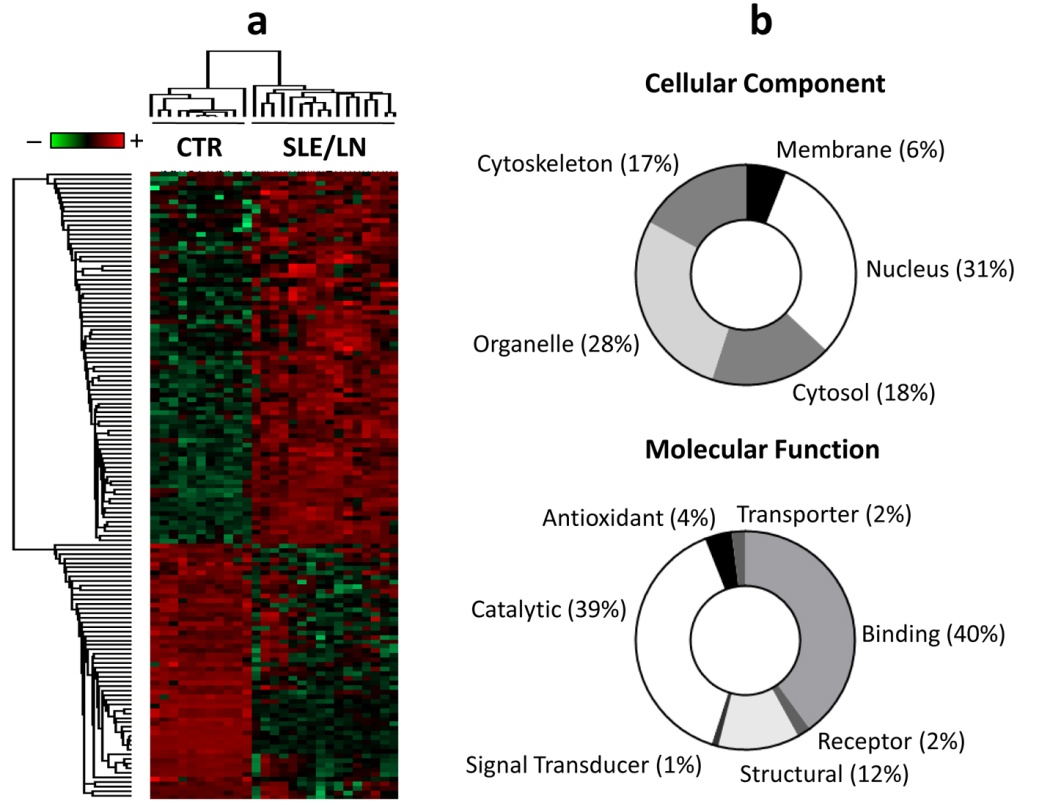


**Figure S-2**

**
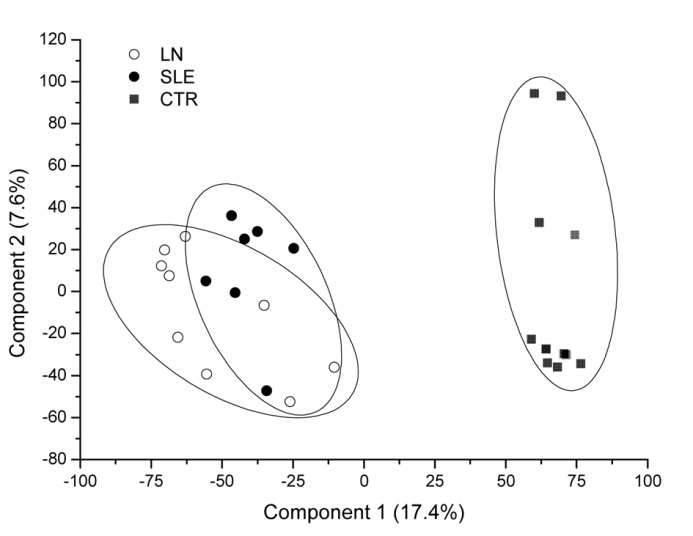
**

**Figure S-3**

**
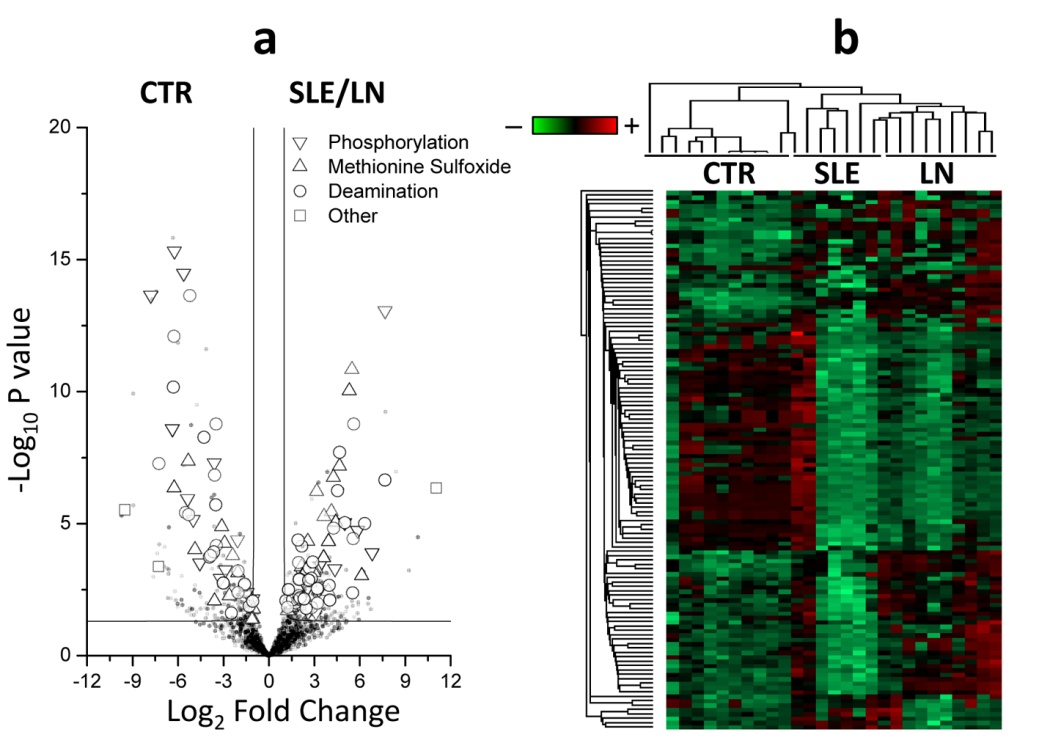
**

**Supplementary TABLES**

**Table S-1.** List of all proteins identified by mass spectrometry in NETs samples. The symbol "+" indicate the presence of the identified proteins in each group, or the association with different diseases (Proteins/diseases association were extracted by Uniprot, ATLAS, Open Target and DisGeNET databases).

| **Protein IDs** | **Protein names** | **Gene names** | **Peptides** | **Unique peptides** | **Sequence coverage [%]** | **Unique sequence coverage [%]** | **MS/MS Count** | **Presence in LN** | **Presence in SLE** | **Presence in Ctr** | **Associated with LUPUS** | **Associated with AutoImmuno Disease** | **Associated with Inflammation** |
| --- | --- | --- | --- | --- | --- | --- | --- | --- | --- | --- | --- | --- | --- |
| A0A0G2JRN3 | Alpha-1-antitrypsin | SERPINA1 | 7 | 7 | 20,3 | 20,3 | 56 | + | + | + | + | + | + |
| H3BT36 | Proteasome subunit alpha type | PSMA2 | 2 | 2 | 72,9 | 72,9 | 13 | + |  | + |  | + |  |
| A0A075B6H6 | Ig kappa chain C region | IGKC | 3 | 3 | 47,7 | 47,7 | 31 | + | + | + |  | + |  |
| A0A075B6L0 | Ig lambda-3 chain C regions | IGLC3 | 4 | 4 | 61,3 | 61,3 | 31 | + | + | + |  |  |  |
| A0A075B754 | ATPase family AAA domain-containing protein 5 | ATAD5 | 1 | 1 | 0,9 | 0,9 | 1 |  |  | + |  | + |  |
| A0A0G2JNW2 | Protein-tyrosine-phosphatase | PTPRC | 7 | 7 | 14,3 | 14,3 | 22 | + | + | + | + | + | + |
| A0A075B7A5 | Leukocyte immunoglobulin-like receptor subfamily A member 3 | LILRA4 | 1 | 1 | 13,6 | 13,6 | 4 | + | + |  |  | + |  |
| E9PIR7 | Thioredoxin reductase 1, cytoplasmic | TXNRD1 | 3 | 3 | 11 | 11 | 6 | + |  | + | + | + |  |
| A0A087WV45 | Transthyretin | TTR | 2 | 2 | 25,2 | 25,2 | 5 |  | + | + | + | + | + |
| A0A087WTK0 | Protein-tyrosine-phosphatase | PTPRJ | 5 | 5 | 5,2 | 5,2 | 15 | + | + | + |  |  |  |
| A0A087WUI2 | Heterogeneous nuclear ribonucleoproteins A2/B1 | HNRNPA2B1 | 3 | 3 | 16,1 | 16,1 | 11 | + | + | + | + | + | + |
| A0A087WUR9 | Low-density lipoprotein receptor-related protein 1B | LRP1B | 1 | 1 | 0,3 | 0,3 | 1 |  |  | + |  | + | + |
| E5RFT7 | Copine-3 | CPNE3 | 1 | 1 | 22,8 | 22,8 | 21 | + | + | + |  | + |  |
| A0A0A6YYL3 | POTE ankyrin domain family member C | POTEB | 1 | 1 | 4,4 | 4,4 | 1 |  |  | + |  |  |  |
| A0A087WUV6 | AT-rich interactive domain-containing protein 1A | ARID1A | 1 | 1 | 1,1 | 1,1 | 1 |  | + |  |  | + | + |
| A0A087WV01 | Elongation factor 1-alpha | EEF1A1 | 1 | 1 | 2,6 | 2,6 | 3 | + | + | + |  | + |  |
| A0A087WV05 | C-Myc-binding protein | MYCBP | 3 | 3 | 29,1 | 29,1 | 9 | + | + | + |  |  |  |
| A0A087WV23 | SH3 domain-binding glutamic acid-rich-like protein 3 | SH3BGRL3 | 7 | 7 | 35,8 | 35,8 | 59 | + | + | + | + | + |  |
| A0A0A0MS07 | Ig gamma-1 chain C region | IGHG1 | 5 | 5 | 24,4 | 24,4 | 17 | + | + | + |  | + |  |
| A0A087WV66 | Antigen KI-67 | MKI67 | 2 | 1 | 0,7 | 0,4 | 2 |  |  | + |  | + | + |
| A0A087WVE2 | Ficolin-1 | FCN1 | 4 | 4 | 7,2 | 7,2 | 15 | + | + | + | + | + | + |
| K7EQH1 | Uncharacterized protein C18orf25 | C18orf25 | 1 | 1 | 5,5 | 5,5 | 3 |  | + |  |  | + |  |
| A0A087WVM2 | CD177 antigen | CD177 | 2 | 2 | 7,1 | 7,1 | 14 | + | + | + | + | + | + |
| A0A087WVT9 | Nucleoside diphosphate kinase | NME4 | 1 | 1 | 7,8 | 7,8 | 4 | + | + |  |  | + |  |
| F8W0M9 | Secernin-3 | SCRN3 | 1 | 1 | 8,1 | 8,1 | 1 |  |  | + |  |  |  |
| A0A087WW30 | 8-oxo-dGDP phosphatase NUDT18 | NUDT18 | 1 | 1 | 9,8 | 9,8 | 6 | + | + |  |  |  |  |
| A0A087X0K8 | Probable G-protein coupled receptor 179 | GPR179 | 1 | 1 | 0,5 | 0,5 | 1 |  |  | + |  |  |  |
| S4R460 | Ig heavy chain V-III region TEI | IGHV3OR16-9 | 1 | 1 | 19,8 | 19,8 | 1 | + |  |  |  |  |  |
| A0A087WW95 | T-box transcription factor TBX3 | TBX3 | 1 | 1 | 4,3 | 4,3 | 1 |  |  | + |  | + | + |
| A0A087WWK2 | Alpha-N-acetylgalactosaminide alpha-2,6-sialyltransferase 3 | ST6GALNAC3 | 1 | 1 | 9,4 | 9,4 | 7 | + | + | + |  |  |  |
| A0A087WX70 |  | PCDH15 | 1 | 1 | 3,4 | 3,4 | 26 | + | + | + |  | + |  |
| A0A087WX84 | A-kinase anchor protein 9 | AKAP9 | 1 | 1 | 1 | 1 | 1 | + |  |  |  | + | + |
| A0A087WXI0 | Ras-related protein Rab-44 | RAB44 | 1 | 1 | 2,3 | 2,3 | 1 |  | + |  |  |  |  |
| A0A087WXI2 | IgGFc-binding protein | FCGBP | 1 | 1 | 0,3 | 0,3 | 1 |  |  | + | + | + | + |
| A0A087WYI3 | Folate receptor gamma | FOLR3 | 3 | 3 | 27,5 | 27,5 | 5 | + | + |  |  |  |  |
| A0A087WXP0 |  | AZU1 | 5 | 2 | 58,7 | 16,7 | 11 | + | + | + |  | + | + |
| Q5TGM0 | Vacuolar protein sorting-associated protein VTA1 homolog | VTA1 | 1 | 1 | 8 | 8 | 1 |  |  | + |  | + |  |
| H0YFY6 | Nuclear mitotic apparatus protein 1 | NUMA1 | 2 | 2 | 2,9 | 2,9 | 10 | + | + | + |  |  | + |
| R4GN62 | Partitioning defective 3 homolog B | PARD3B | 1 | 1 | 1,8 | 1,8 | 1 |  |  | + |  | + |  |
| A0A087WZC3 | Synaptonemal complex protein 1 | SYCP1 | 1 | 1 | 1,3 | 1,3 | 11 | + | + | + |  |  |  |
| A0A087WZE4 | Spectrin alpha chain, erythrocytic 1 | SPTA1 | 16 | 16 | 7,1 | 7,1 | 23 | + | + | + |  |  |  |
| C9JXK9 | Lipoma-preferred partner | LPP | 1 | 1 | 5,6 | 5,6 | 1 |  |  | + | + | + | + |
| A0A087WZH7 | Myristoylated alanine-rich C-kinase substrate | MARCKS | 5 | 5 | 13,9 | 13,9 | 10 | + | + | + |  | + | + |
| D6RHI9 | Ribonuclease T2 | RNASET2 | 6 | 6 | 24,4 | 24,4 | 6 | + |  | + |  | + |  |
| A0A087WZR4 | Low affinity immunoglobulin gamma Fc region receptor III-B | FCGR3B | 5 | 2 | 20,8 | 9,7 | 50 | + | + | + | + | + | + |
| A0A0C4DFV9 | Protein SET | SET | 2 | 2 | 8,6 | 8,6 | 6 | + | + |  |  | + |  |
| A0A087X0E7 |  | C18orf21 | 1 | 1 | 12,3 | 12,3 | 1 | + |  |  |  |  |  |
| A0A087X0L4 | Interferon-inducible protein AIM2 | AIM2 | 1 | 1 | 2,3 | 2,3 | 6 | + | + |  | + | + | + |
| A0A087X0P0 | Kinesin-like protein | CENPE | 1 | 1 | 0,4 | 0,4 | 2 | + |  | + |  | + |  |
| A0A087X0P5 | Alkaline phosphatase | ALPPL2 | 1 | 1 | 3,4 | 3,4 | 1 | + |  |  |  |  |  |
| A0A087X0U0 | Mediator of RNA polymerase II transcription subunit 4 | MED4 | 1 | 1 | 18,3 | 18,3 | 1 |  |  | + |  |  |  |
| A0A087X1H5 | Arf-GAP with coiled-coil, ANK repeat and PH domain-containing protein 2 | ACAP2 | 4 | 4 | 4,6 | 4,6 | 3 | + | + | + |  | + |  |
| A0A087X1N2 | CCAAT/enhancer-binding protein zeta | CEBPZ | 1 | 1 | 2,4 | 2,4 | 1 |  |  | + | + | + | + |
| A0A087X1N7 | Nebulin | NEB | 4 | 4 | 1,1 | 1,1 | 4 |  |  | + |  | + |  |
| A0A087X1S2 | Nuclease-sensitive element-binding protein 1 | YBX1 | 2 | 2 | 7,5 | 7,5 | 16 | + | + | + | + | + | + |
| A0A087X208 | Agrin | AGRN | 2 | 2 | 1,5 | 1,5 | 4 |  | + | + | + | + | + |
| A0A087X253 | AP-2 complex subunit beta | AP2B1 | 14 | 14 | 16 | 16 | 86 | + | + | + |  |  |  |
| H7BY63 | Protein FAM184A | FAM184A | 1 | 1 | 1,2 | 1,2 | 0 |  |  | + |  |  |  |
| A0A087X2C1 | Sorting nexin-20 | SNX20 | 1 | 1 | 7,9 | 7,9 | 4 | + | + | + |  | + |  |
| A0A096LPD5 | Serpin B11 | SERPINB11 | 1 | 1 | 6,2 | 6,2 | 1 |  |  | + |  |  | + |
| A0A0A0MR61 | Serine protease 57 | PRSS57 | 3 | 3 | 18,1 | 18,1 | 18 | + |  | + |  |  |  |
| A0A0A0MRY2 | Zinc finger MYND domain-containing protein 11 | ZMYND11 | 1 | 1 | 1,7 | 1,7 | 0 | + |  |  |  | + |  |
| A0A0A0MS53 | Methylcytosine dioxygenase TET2 | TET2 | 1 | 1 | 5,3 | 5,3 | 0 |  |  | + | + | + | + |
| F8VYN8 | Centrosomal protein of 83 kDa | CEP83 | 2 | 2 | 5,7 | 5,7 | 4 | + | + | + |  |  |  |
| A0A0A0MSC8 | Slit homolog 3 protein | SLIT3 | 1 | 1 | 1 | 1 | 1 |  |  | + |  | + | + |
| A0A0A0MSI0 | Peroxiredoxin-1 | PRDX1 | 3 | 2 | 25,7 | 19,3 | 7 | + | + | + |  | + | + |
| A0A0A0MSK1 | Angiopoietin-related protein 1 | ANGPTL1 | 1 | 1 | 20,2 | 20,2 | 1 |  |  | + |  |  | + |
| A0A0A0MSK5 | Torsin-1A-interacting protein 1 | TOR1AIP1 | 2 | 2 | 5 | 5 | 2 | + |  |  |  | + |  |
| H3BS90 | Calcium-transporting ATPase | ATP2C2 | 1 | 1 | 13,2 | 13,2 | 0 |  |  | + |  |  |  |
| A0A0A0MSP7 | FERM and PDZ domain-containing protein 3 | FRMPD3 | 1 | 1 | 0,7 | 0,7 | 2 | + | + |  |  |  |  |
| F6UBH9 | Zinc fingers and homeoboxes protein 3 | ZHX3 | 1 | 1 | 23,9 | 23,9 | 1 |  |  | + |  | + |  |
| H7C4R7 | Deoxyribonuclease | DNASE1L3 | 1 | 1 | 5,8 | 5,8 | 43 | + | + | + | + | + |  |
| A0A0A0MTK7 | Protein kinase C-binding protein 1 | ZMYND8 | 2 | 2 | 2,2 | 2,2 | 1 |  |  | + |  | + |  |
| E5RHK4 | La-related protein 1 | LARP1 | 1 | 1 | 17,2 | 17,2 | 3 | + | + |  |  | + | + |
| F8WCN2 |  | TM9SF4 | 1 | 1 | 38,2 | 38,2 | 1 |  |  | + |  | + |  |
| A0A0C4DFU2 | Superoxide dismutase | SOD2 | 4 | 4 | 24,3 | 24,3 | 15 | + | + | + |  | + | + |
| A0A0C4DFX7 | WD repeat-containing protein 76 | WDR76 | 1 | 1 | 2,3 | 2,3 | 2 |  |  | + |  | + |  |
| A0A0C4DGA6 | Helicase-like transcription factor | HLTF | 1 | 1 | 1 | 1 | 4 | + | + | + |  | + | + |
| A0A0C4DGH5 | Cullin-associated NEDD8-dissociated protein 1 | CAND1 | 1 | 1 | 1,8 | 1,8 | 1 |  |  | + |  |  |  |
| F8W642 | Citrate synthase | CS | 1 | 1 | 27,3 | 27,3 | 2 | + |  |  |  |  |  |
| A0A0C4DGJ9 | Granzyme H | GZMH | 1 | 1 | 8,7 | 8,7 | 35 | + | + | + |  | + | + |
| A0A0C4DGP4 | Glucosidase 2 subunit beta | PRKCSH | 1 | 1 | 6,5 | 6,5 | 7 | + |  | + |  | + |  |
| C9J7N5 | Serpin I2 | SERPINI2 | 10 | 9 | 35,9 | 31,1 | 106 | + | + | + |  | + | + |
| A0A0D9SFR6 | Diacylglycerol kinase eta | DGKH | 1 | 1 | 1,8 | 1,8 | 2 |  |  | + |  |  |  |
| Q5SRN7 | HLA class I histocompatibility antigen, Cw-16 alpha chain | HLA-A | 2 | 1 | 12,7 | 8 | 2 | + |  | + |  | + |  |
| A0A0G2JIW1 | Heat shock 70 kDa protein 1B | HSPA1B | 27 | 13 | 46,3 | 24,6 | 330 | + | + | + | + | + | + |
| A0A0G2JPZ0 | Immunoglobulin alpha Fc receptor | FCAR | 1 | 1 | 10,3 | 10,3 | 1 | + |  |  | + | + | + |
| H0YCV8 | Transcription factor TFIIIB component B homolog | BDP1 | 1 | 1 | 2,1 | 2,1 | 1 |  |  | + |  | + |  |
| Q5VZZ6 | CUGBP Elav-like family member 2 | CELF2 | 2 | 2 | 4,8 | 4,8 | 20 | + | + | + | + | + |  |
| A0A0J9YXC4 |  |  | 1 | 1 | 87,5 | 87,5 | 7 | + |  | + |  |  |  |
| A0A0U1RQI2 |  |  | 1 | 1 | 41,5 | 41,5 | 1 |  |  | + |  |  |  |
| A0A0U1RQL8 |  |  | 7 | 2 | 32,3 | 7,3 | 7 | + | + | + |  |  |  |
| A0A0U1RRJ3 |  |  | 2 | 2 | 2,4 | 2,4 | 8 | + | + | + |  |  |  |
| A0A0U1RQV4 | Rho-associated protein kinase 1 | ROCK1 | 1 | 1 | 1 | 1 | 4 | + | + |  |  | + | + |
| E7EPU2 | Disco-interacting protein 2 homolog C | DIP2C | 1 | 1 | 2 | 2 | 1 |  |  | + |  |  |  |
| A0A0U1RQY8 | Copper-transporting ATPase 2 | ATP7B | 1 | 1 | 2 | 2 | 1 | + |  |  |  |  | + |
| A0A0U1RR22 | Protein kinase C and casein kinase substrate in neurons protein 2 | PACSIN2 | 2 | 2 | 4,9 | 4,9 | 2 |  | + |  |  |  |  |
| A0A0U1RR32 | Histone H2A type 1-J | HIST1H2AJ | 4 | 4 | 27,2 | 27,2 | 11 |  | + | + |  |  |  |
| A0A0U1RRH6 | PHD finger protein 14 | PHF14 | 1 | 1 | 1,6 | 1,6 | 1 |  |  | + |  |  |  |
| A0A0X1KG69 |  |  | 1 | 1 | 2,5 | 2,5 | 1 |  |  | + |  |  |  |
| A0JP02 | Pleckstrin homology domain-containing family A member 5 | PLEKHA5 | 1 | 1 | 2,4 | 2,4 | 0 |  |  | + |  | + |  |
| A1A4F0 | Putative uncharacterized protein PQLC2L | PQLC2L | 1 | 1 | 11,1 | 11,1 | 2 |  |  | + |  | + |  |
| H7BYP1 | Transient receptor potential cation channel subfamily M member 3 | TRPM3 | 3 | 3 | 2,6 | 2,6 | 5 |  |  | + |  | + | + |
| A2A3R5 | 40S ribosomal protein S6 | RPS6 | 5 | 5 | 15,1 | 15,1 | 20 | + | + | + |  | + |  |
| A4D1F6 | Leucine-rich repeat and death domain-containing protein 1 | LRRD1 | 1 | 1 | 1,9 | 1,9 | 1 |  |  | + |  |  |  |
| A4FU16 |  | RHBDL3 | 1 | 1 | 3,8 | 3,8 | 1 |  |  | + |  |  |  |
| A6NC48 | ADP-ribosyl cyclase/cyclic ADP-ribose hydrolase 2 | BST1 | 7 | 7 | 24,6 | 24,6 | 23 | + | + | + |  | + |  |
| A6NCN2 | Putative keratin-87 protein | KRT87P | 25 | 4 | 53,7 | 7,8 | 27 | + | + | + |  |  |  |
| A6NED7 | Volume-regulated anion channel subunit LRRC8C | LRRC8C | 1 | 1 | 25 | 25 | 1 |  |  | + |  | + |  |
| A6NGU7-2 |  |  | 1 | 1 | 13,5 | 13,5 | 1 |  |  | + |  |  |  |
| B5MC82 | D-dopachrome decarboxylase | DDT | 2 | 2 | 16,9 | 16,9 | 4 | + |  | + | + | + | + |
| K7EIR6 | Zinc finger protein 233 | ZNF112 | 1 | 1 | 20,4 | 20,4 | 1 |  |  | + |  |  |  |
| A6NMQ3 | Alpha-endosulfine | ENSA | 1 | 1 | 10 | 10 | 1 |  |  | + |  | + |  |
| A8K878 | Mesencephalic astrocyte-derived neurotrophic factor | MANF | 2 | 2 | 15,7 | 15,7 | 2 | + | + |  | + | + | + |
| V9GY10 | Protection of telomeres protein 1 | POT1 | 1 | 1 | 28,6 | 28,6 | 1 |  |  | + | + | + | + |
| A8MUA9 | Small ubiquitin-related modifier 2 | SUMO3 | 1 | 1 | 8,9 | 8,9 | 2 | + |  |  |  |  |  |
| H7C571 | Transcription cofactor vestigial-like protein 3 | VGLL3 | 1 | 1 | 7,7 | 7,7 | 1 |  | + |  |  | + |  |
| M0R1E7 | Serine/arginine repetitive matrix protein 1 | SRRM1 | 1 | 1 | 21,5 | 21,5 | 1 |  |  | + |  | + |  |
| B0YIW6 | Coatomer subunit delta | ARCN1 | 1 | 1 | 2,2 | 2,2 | 1 | + |  |  |  | + |  |
| B1AHD3 | Pyridoxal phosphate phosphatase | PDXP | 1 | 1 | 17,7 | 17,7 | 2 | + |  | + |  |  |  |
| B1AKP8 | Serine/threonine-protein kinase mTOR | MTOR | 1 | 1 | 1,9 | 1,9 | 1 |  |  | + | + | + | + |
| B1ALD9 | Periostin | POSTN | 1 | 1 | 1,7 | 1,7 | 1 |  |  | + |  | + | + |
| H0YDI1 | Lymphocyte function-associated antigen 3 | CD58 | 1 | 1 | 13,7 | 13,7 | 1 |  |  | + | + | + | + |
| B2R4S9 | Histone H2B | HIST1H2BI | 6 | 1 | 43,7 | 7,9 | 104 | + | + | + |  |  |  |
| G3V576 | Heterogeneous nuclear ribonucleoproteins C1/C2 | HNRNPC | 4 | 4 | 21,6 | 21,6 | 23 | + | + | + |  |  |  |
| Q5T7C4 | Putative high mobility group protein B1-like 1 | HMGB1 | 5 | 5 | 21,5 | 21,5 | 29 | + | + |  | + | + | + |
| K7EMV3 | Histone H3 | H3F3B | 4 | 3 | 22,8 | 15,2 | 36 | + | + | + | + | + |  |
| B4DIP2 | Protein LAP2 | ERBB2IP | 1 | 1 | 2 | 2 | 1 |  |  | + |  |  |  |
| E9PRD9 | Vascular non-inflammatory molecule 2 | VNN2 | 3 | 3 | 40,2 | 40,2 | 27 | + | + | + |  | + | + |
| J3QS39 | Ubiquitin-60S ribosomal protein L40 | UBB | 10 | 6 | 71 | 32,3 | 188 | + | + | + |  | + | + |
| E9PPA1 | Oxysterols receptor LXR-alpha | NR1H3 | 1 | 1 | 7,8 | 7,8 | 1 |  |  | + | + | + | + |
| B4DXW1 | Actin-related protein 3 | ACTR3 | 2 | 2 | 3,3 | 3,3 | 9 | + | + |  |  | + |  |
| G3XAN3 | MCM domain-containing protein 2 | MCMDC2 | 1 | 1 | 6,5 | 6,5 | 1 |  |  | + |  |  |  |
| H0YHL7 | Coronin | CORO1C | 1 | 1 | 7,3 | 7,3 | 4 | + | + |  |  |  |  |
| H7C3M2 | 60S ribosomal protein L3 | RPL3 | 1 | 1 | 7,3 | 7,3 | 1 |  |  | + |  | + |  |
| Q14500 | ATP-sensitive inward rectifier potassium channel 12 | KCNJ12 | 1 | 1 | 3,7 | 3,7 | 1 |  |  | + |  | + |  |
| H0Y8F7 | Dihydropteridine reductase | QDPR | 1 | 1 | 10,9 | 10,9 | 1 |  | + |  |  | + |  |
| F5H7S3 | Tropomyosin alpha-1 chain | TPM1 | 4 | 1 | 17,1 | 4,9 | 2 | + | + |  |  | + | + |
| F6UXX1 | Heterogeneous nuclear ribonucleoprotein Q | SYNCRIP | 1 | 1 | 7 | 7 | 2 | + |  | + |  | + | + |
| F8W1R7 | Myosin light polypeptide 6 | MYL6 | 7 | 7 | 51,7 | 51,7 | 30 | + | + | + |  |  |  |
| K7EIJ4 | Ran-binding protein 3 | RANBP3 | 3 | 3 | 26,5 | 26,5 | 8 | + |  | + |  | + |  |
| B7Z7G7 | Vacuolar protein sorting-associated protein 45 | VPS45 | 2 | 2 | 14,1 | 14,1 | 2 |  |  | + |  |  |  |
| B7ZBK6 | Delta-aminolevulinic acid dehydratase | ALAD | 2 | 2 | 29,2 | 29,2 | 2 | + |  |  |  | + |  |
| B7ZKW8 | CapZ-interacting protein | RCSD1 | 1 | 1 | 4,1 | 4,1 | 4 | + | + |  |  | + |  |
| B8ZZQ6 | Prothymosin alpha | PTMA | 4 | 4 | 27,1 | 27,1 | 39 | + | + | + | + | + | + |
| C9J524 | Calcyphosin-2 | CAPS2 | 1 | 1 | 11,1 | 11,1 | 1 |  |  | + |  |  |  |
| CON__Q92764 | Keratin, type I cuticular Ha5 | KRT35 | 11 | 2 | 20,5 | 7,5 | 4 | + | + | + |  |  |  |
| Q5VVQ1 | Translin-associated protein X | TSNAX | 1 | 1 | 12,3 | 12,3 | 2 | + | + |  |  |  |  |
| C9IYS5 |  | PHYH | 1 | 1 | 9,1 | 9,1 | 1 |  |  | + |  | + |  |
| C9IZG4 | Protein CutA | CUTA | 2 | 2 | 19,3 | 19,3 | 10 | + | + | + |  |  |  |
| C9J330 | Nuclear receptor corepressor 2 | NCOR2 | 1 | 1 | 3,2 | 3,2 | 1 |  |  | + |  |  | + |
| C9J250 |  | RBM6 | 1 | 1 | 10,9 | 10,9 | 1 |  |  | + |  |  |  |
| C9J4A7 | Pleckstrin homology domain-containing family O member 2 | PLEKHO2 | 1 | 1 | 6,2 | 6,2 | 1 |  | + |  |  | + |  |
| C9J7B7 | 26S proteasome non-ATPase regulatory subunit 6 | PSMD6 | 1 | 1 | 11,8 | 11,8 | 8 | + | + | + |  | + |  |
| C9J813 | Caldesmon | CALD1 | 3 | 3 | 2,2 | 2,2 | 30 | + | + | + |  | + | + |
| C9J8P9 | Clathrin light chain A | CLTA | 1 | 1 | 4,6 | 4,6 | 1 |  |  | + | + | + |  |
| C9J9W2 | LIM and SH3 domain protein 1 | LASP1 | 1 | 1 | 8,4 | 8,4 | 4 | + | + |  |  | + | + |
| C9JAA7 | Armadillo repeat-containing protein 8 | ARMC8 | 1 | 1 | 6,1 | 6,1 | 1 |  |  | + |  | + |  |
| C9JDW2 | Latent-transforming growth factor beta-binding protein 1 | LTBP1 | 2 | 2 | 9,6 | 9,6 | 19 | + | + | + |  |  | + |
| C9JGQ2 |  | MAP3K19 | 1 | 1 | 76,2 | 76,2 | 1 |  |  | + |  |  |  |
| C9JEV0 | Zinc-alpha-2-glycoprotein | AZGP1 | 1 | 1 | 5,7 | 5,7 | 5 | + | + |  |  | + | + |
| C9JF14 | Caspase-5 | CASP5 | 1 | 1 | 15,2 | 15,2 | 1 |  |  | + |  | + | + |
| C9JFP8 |  | SHANK2 | 1 | 1 | 23 | 23 | 1 |  |  | + |  |  |  |
| F8WEA2 |  | NME8 | 1 | 1 | 37 | 37 | 1 |  |  | + |  | + |  |
| H7C393 | Acylamino-acid-releasing enzyme | APEH | 4 | 4 | 16,9 | 16,9 | 5 | + |  | + |  | + | + |
| C9JZN1 | Guanine nucleotide-binding protein G(I)/G(S)/G(T) subunit beta-2 | GNB2 | 5 | 2 | 42,1 | 21,1 | 5 | + |  | + |  |  |  |
| C9JIZ6 | Prosaposin | PSAP | 5 | 5 | 7,2 | 7,2 | 25 | + | + | + |  | + | + |
| J3KS76 |  | KIAA0195 | 1 | 1 | 11,1 | 11,1 | 5 |  |  | + |  |  |  |
| C9JN86 |  | ANK1 | 1 | 1 | 11,5 | 11,5 | 2 |  |  | + |  | + | + |
| C9JRY4 | Vesicle-trafficking protein SEC22a | SEC22A | 1 | 1 | 5 | 5 | 2 | + |  |  |  |  |  |
| C9JQ42 | Glycogenin-1 | GYG1 | 7 | 7 | 27,4 | 27,4 | 76 | + | + | + | + | + |  |
| U3KPY6 | Prospero homeobox protein 1 | PROX1 | 1 | 1 | 19,6 | 19,6 | 1 |  |  | + |  |  |  |
| C9JU59 | Lymphocyte-specific protein 1 | LSP1 | 3 | 3 | 26,9 | 26,9 | 20 | + | + |  | + | + | + |
| C9JUY1 |  | AFF3 | 1 | 1 | 4,9 | 4,9 | 8 | + |  | + | + | + |  |
| C9JVN1 | Melanotransferrin | MFI2 | 1 | 1 | 4,4 | 4,4 | 3 |  |  | + |  |  |  |
| C9JZR7 |  | ACTB | 9 | 1 | 67 | 7,8 | 25 | + | + | + | + | + | + |
| C9K0D8 | 6-phosphofructo-2-kinase/fructose-2,6-bisphosphatase 4 | PFKFB4 | 1 | 1 | 6,9 | 6,9 | 2 |  |  | + |  |  |  |
| C9K0I0 | Testis-specific serine kinase substrate | TSKS | 1 | 1 | 3,3 | 3,3 | 1 |  |  | + |  |  |  |
| CON__A2A5Y0 |  |  | 21 | 2 | 33,4 | 4,1 | 18 | + | + | + |  |  |  |
| F8W7U0 | Intersectin-1 | ITSN1 | 2 | 1 | 3,6 | 2,8 | 1 |  |  | + |  | + | + |
| CON__ENSEMBL:ENSBTAP00000014147 | |  | 2 | 2 | 25,7 | 25,7 | 31 | + | + | + |  |  |  |
| CON__Q3MHN5 | |  | 10 | 10 | 30 | 30 | 44 | + | + | + |  |  |  |
| CON__ENSEMBL:ENSBTAP00000023055 | |  | 1 | 1 | 1,9 | 1,9 | 13 | + | + | + |  |  |  |
| CON__ENSEMBL:ENSBTAP00000024146 | |  | 2 | 2 | 2,2 | 2,2 | 2 |  |  | + |  |  |  |
| CON__ENSEMBL:ENSBTAP00000024466 | |  | 3 | 3 | 21,2 | 21,2 | 22 | + | + | + |  |  |  |
| CON__ENSEMBL:ENSBTAP00000031360 | |  | 6 | 6 | 22,8 | 22,8 | 24 | + | + | + |  |  |  |
| CON__ENSEMBL:ENSBTAP00000033053 | |  | 2 | 1 | 25,9 | 19,4 | 5 | + | + | + |  |  |  |
| CON__ENSEMBL:ENSBTAP00000038253 | |  | 14 | 1 | 17,8 | 1,8 | 2 | + | + | + |  |  |  |
| K7ERE3 | Keratin, type I cytoskeletal 13 | KRT13 | 7 | 1 | 14 | 3,9 | 0 | + | + | + |  |  | + |
| CON__O43790 | Keratin, type II cuticular Hb6 | KRT86 | 29 | 2 | 42,2 | 9,1 | 11 | + | + | + |  | + |  |
| CON__O76013 | Keratin, type I cuticular Ha6 | KRT36 | 10 | 5 | 14,6 | 7,7 | 23 | + | + | + | + | + | + |
| CON__O76014 | Keratin, type I cuticular Ha7 | KRT37 | 5 | 1 | 10 | 3,8 | 1 | + | + | + |  |  | + |
| CON__O95678 | Keratin, type II cytoskeletal 75 | KRT75 | 14 | 2 | 16,7 | 2,2 | 4 | + | + | + |  | + |  |
| CON__P00761 |  |  | 12 | 11 | 62,3 | 58,9 | 1043 | + | + | + |  |  |  |
| CON__P00766 |  |  | 18 | 18 | 94,7 | 94,7 | 5543 | + | + | + |  |  |  |
| CON__P00978 |  |  | 8 | 8 | 20,7 | 20,7 | 166 | + | + | + |  |  |  |
| CON__P01966 |  |  | 8 | 3 | 38 | 20,4 | 103 | + | + | + |  |  |  |
| CON__P02070 |  |  | 5 | 2 | 43,4 | 22,8 | 13 | + | + | + |  |  |  |
| CON__P02533 | Keratin, type I cytoskeletal 14 | KRT14 | 23 | 6 | 45,6 | 17,4 | 110 | + | + | + |  | + | + |
| CON__P02538 | Keratin, type II cytoskeletal 6A | KRT6A | 29 | 1 | 34,4 | 3,9 | 3 | + | + | + |  | + |  |
| CON__P02768-1 | Serum albumin | ALB | 83 | 78 | 85,9 | 81,9 | 1218 | + | + | + | + | + | + |
| CON__P02769 |  |  | 88 | 83 | 87,6 | 82,5 | 2896 | + | + | + |  |  |  |
| CON__P06868 |  |  | 11 | 11 | 20,7 | 20,7 | 67 | + | + | + |  |  |  |
| CON__P07477 | Trypsin-1 | PRSS1 | 3 | 2 | 15,4 | 12,1 | 54 | + | + | + | + | + | + |
| CON__Q3KNV1 | Keratin, type II cytoskeletal 7 | KRT7 | 5 | 0 | 6,8 | 0 | 2 | + | + | + |  | + | + |
| CON__P08779 | Keratin, type I cytoskeletal 16 | KRT16 | 20 | 8 | 40,4 | 22,2 | 25 | + | + | + |  | + | + |
| CON__P13645 | Keratin, type I cytoskeletal 10 | KRT10 | 43 | 35 | 55,1 | 50,6 | 597 | + | + | + | + | + | + |
| CON__P13647 | Keratin, type II cytoskeletal 5 | KRT5 | 28 | 5 | 36,4 | 11 | 17 | + | + | + |  |  | + |
| CON__P17690 |  |  | 7 | 7 | 23,2 | 23,2 | 23 | + | + | + |  |  |  |
| CON__P34955 |  |  | 11 | 11 | 26,2 | 26,2 | 40 | + | + | + |  |  |  |
| CON__P35908 | Keratin, type II cytoskeletal 2 epidermal | KRT2 | 40 | 25 | 53,5 | 41,6 | 205 | + | + | + |  | + |  |
| CON__P41361 |  |  | 5 | 5 | 16,8 | 16,8 | 11 | + | + | + |  |  |  |
| CON__P48668 | Keratin, type II cytoskeletal 6C | KRT6C | 30 | 0 | 36,2 | 0 | 12 | + | + | + |  |  |  |
| CON__P67983 |  |  | 4 | 2 | 50,8 | 29,5 | 8 | + | + | + |  |  |  |
| CON__P78386 | Keratin, type II cuticular Hb5 | KRT85 | 29 | 6 | 35,5 | 11,6 | 22 | + | + | + |  |  |  |
| CON__Q04695 | Keratin, type I cytoskeletal 17 | KRT17 | 16 | 4 | 33,6 | 13,7 | 6 | + | + | + |  | + | + |
| CON__Q05B55 |  |  | 3 | 3 | 23,3 | 23,3 | 4 | + | + |  |  |  |  |
| CON__Q29443 |  |  | 7 | 3 | 11,8 | 7,4 | 7 | + | + | + |  |  |  |
| CON__Q14525 | Keratin, type I cuticular Ha3-II | KRT33B | 18 | 2 | 37,6 | 3 | 3 | + | + | + |  |  |  |
| CON__Q9UE12 | Keratin, type I cuticular Ha1 | KRT31 | 22 | 0 | 47,1 | 0 | 64 | + | + | + |  |  |  |
| CON__Q1RMK2 | |  | 3 | 2 | 7,7 | 6,2 | 42 | + | + | + |  |  |  |
| CON__Q1RMN8 | |  | 11 | 11 | 42,3 | 42,3 | 481 | + | + | + |  |  |  |
| CON__Q29RQ1 |  |  | 16 | 16 | 21,9 | 21,9 | 135 | + | + | + |  |  |  |
| CON__Q32PI4 |  |  | 3 | 3 | 4,7 | 4,7 | 3 |  | + | + |  |  |  |
| CON__Q3KUS7 |  |  | 1 | 1 | 2,8 | 2,8 | 4 | + | + | + |  |  |  |
| CON__Q3SX09 |  |  | 4 | 1 | 22,9 | 8 | 2 | + | + | + |  |  |  |
| CON__Q3SZV7 |  |  | 9 | 9 | 22,8 | 22,8 | 43 | + | + | + |  |  |  |
| CON__Q3Y5Z3 |  |  | 7 | 7 | 28,8 | 28,8 | 106 | + | + | + |  |  |  |
| CON__Q9Z2K1 |  |  | 7 | 1 | 13,6 | 4,9 | 1 | + | + | + |  |  |  |
| CON__Q5D862 | Filaggrin-2 | FLG2 | 1 | 1 | 0,5 | 0,5 | 1 | + |  |  |  |  |  |
| CON__Q5XKE5 | Keratin, type II cytoskeletal 79 | KRT79 | 10 | 0 | 14 | 0 | 1 | + | + | + |  |  |  |
| CON__Q61726 |  |  | 25 | 6 | 28,6 | 11,5 | 21 | + | + | + |  |  |  |
| CON__Q9NSB2 | Keratin, type II cuticular Hb4 | KRT84 | 10 | 6 | 19,8 | 14,5 | 5 | + | + | + |  |  |  |
| CON__Q6NT21 | Keratin, type II cuticular Hb3 | KRT83 | 30 | 1 | 38,5 | 2,4 | 90 | + | + | + |  |  |  |
| CON__Q9D646 |  |  | 15 | 0 | 30,9 | 0 | 5 | + | + | + |  |  |  |
| CON__Q9NSB4 | Keratin, type II cuticular Hb2 | KRT82 | 8 | 3 | 10,1 | 2,7 | 7 | + | + | + |  |  | + |
| CON__Q9R0H5 | Keratin, type II cytoskeletal 71 | KRT71 | 3 | 1 | 5,9 | 1,7 | 1 | + | + | + |  | + | + |
| CON__Q9TTE1 |  |  | 3 | 3 | 9,2 | 9,2 | 4 | + | + | + |  |  |  |
| CON__REFSEQ:XP_986630 | |  | 15 | 1 | 20,4 | 2,5 | 1 | + | + | + |  |  |  |
| D3DSM0 | Integrin beta | ITGB2 | 24 | 24 | 40 | 40 | 209 | + | + | + | + | + | + |
| D6R9A6 | High mobility group protein B2 | HMGB2 | 5 | 5 | 19,4 | 19,4 | 87 | + | + | + | + | + | + |
| D6RD83 | Heterogeneous nuclear ribonucleoprotein D0 | HNRNPD | 2 | 2 | 18,5 | 18,5 | 5 | + |  |  | + | + | + |
| D6RCA8 | Annexin | ANXA3 | 11 | 1 | 64,2 | 8,2 | 1 | + | + | + | + | + | + |
| D6RD63 | COP9 signalosome complex subunit 4 | COPS4 | 1 | 1 | 5,5 | 5,5 | 1 |  |  | + |  |  |  |
| D6RG15 | Twinfilin-2 | TWF2 | 3 | 3 | 18,1 | 18,1 | 12 | + | + | + |  |  |  |
| F6S289 | Pentatricopeptide repeat-containing protein 2, mitochondrial | PTCD2 | 1 | 1 | 5,6 | 5,6 | 1 |  |  | + |  |  |  |
| D6RGX7 |  | DCUN1D4 | 1 | 1 | 27 | 27 | 1 |  |  | + |  |  |  |
| D6RJB7 | Putative ankyrin repeat domain-containing protein 31 | ANKRD31 | 1 | 1 | 1,1 | 1,1 | 1 |  |  | + |  |  |  |
| E5RFR7 | Tumor protein D52 | TPD52 | 3 | 3 | 18,9 | 18,9 | 24 | + | + | + |  | + |  |
| E5RG43 |  | CA1 | 14 | 1 | 83,2 | 7,4 | 1 | + | + | + | + | + | + |
| E5RG74 | Brain-specific angiogenesis inhibitor 1 | ADGRB1 | 1 | 1 | 2,3 | 2,3 | 1 |  |  | + |  |  | + |
| E5RGE1 | 14-3-3 protein zeta/delta | YWHAZ | 1 | 1 | 26,9 | 26,9 | 8 | + | + | + |  | + | + |
| E5RHP7 | Carbonic anhydrase 1 | CA1 | 20 | 0 | 64,5 | 0 | 303 | + | + | + | + | + | + |
| E5RI69 |  | TATDN1 | 1 | 1 | 28,6 | 28,6 | 1 |  |  | + |  |  |  |
| E5RJI8 |  | CA1 | 9 | 1 | 53,6 | 10,9 | 1 | + | + | + | + | + | + |
| H0YB43 | Proton-coupled amino acid transporter 2 | SLC36A2 | 1 | 1 | 25,2 | 25,2 | 1 |  |  | + |  | + |  |
| E5RJL1 | Transporter | SLC6A7 | 1 | 1 | 3,5 | 3,5 | 2 |  |  | + |  |  |  |
| E7EN95 | Filamin-B | FLNB | 9 | 8 | 4,4 | 4,1 | 19 | + | + | + | + | + |  |
| E7EPZ9 | Tenascin-X | TNXB | 2 | 2 | 0,4 | 0,4 | 6 | + | + | + | + | + | + |
| H0Y8L0 | PHD finger protein 3 | PHF3 | 1 | 1 | 8,9 | 8,9 | 1 |  |  | + |  | + |  |
| E7ER45 | Maltase-glucoamylase, intestinal | MGAM | 5 | 5 | 5 | 5 | 18 | + | + | + |  | + |  |
| E7ES84 | Kinetochore-associated protein 1 | KNTC1 | 1 | 1 | 1 | 1 | 2 | + |  |  |  |  |  |
| E7EVM7 | Piezo-type mechanosensitive ion channel component | PIEZO2 | 1 | 1 | 0,5 | 0,5 | 1 |  |  | + |  | + |  |
| E7EVZ1 | Zinc finger homeobox protein 4 | ZFHX4 | 1 | 1 | 0,7 | 0,7 | 1 |  |  | + |  |  |  |
| E7EW16 | Alpha-1A adrenergic receptor | ADRA1A | 1 | 1 | 2,6 | 2,6 | 1 |  |  | + |  |  |  |
| E7EWD9 | Steroid hormone receptor ERR2 | ESRRB | 1 | 1 | 2,8 | 2,8 | 1 |  | + |  |  |  |  |
| E9PAQ1 | Properdin | CFP | 2 | 2 | 8 | 8 | 9 | + | + | + | + | + | + |
| E9PDH4 | 1-phosphatidylinositol 3-phosphate 5-kinase | PIKFYVE | 1 | 1 | 1,1 | 1,1 | 3 | + |  | + |  | + | + |
| E9PDP5 | Ankyrin repeat and KH domain-containing protein 1 | ANKHD1 | 1 | 1 | 1 | 1 | 0 |  |  | + |  | + |  |
| E9PEI0 | Cell division cycle-associated protein 2 | CDCA2 | 1 | 1 | 3,1 | 3,1 | 1 |  |  | + |  | + |  |
| E9PEW8 |  | HBD | 17 | 1 | 96,2 | 7,7 | 40 | + | + | + | + | + | + |
| E9PGG2 | Anomalous homeobox protein | ANHX | 1 | 1 | 6,1 | 6,1 | 1 |  |  | + |  |  |  |
| E9PH88 |  | PMS1 | 1 | 1 | 5,3 | 5,3 | 2 |  |  | + | + | + |  |
| E9PIF2 | Probable ATP-dependent RNA helicase DDX10 | DDX10 | 1 | 1 | 1,4 | 1,4 | 2 |  | + | + |  |  |  |
| E9PJ32 | Transgelin | TAGLN | 1 | 1 | 12,1 | 12,1 | 1 |  |  | + | + | + | + |
| E9PJ90 | HBS1-like protein | HBS1L | 1 | 1 | 9,3 | 9,3 | 1 |  | + |  |  |  |  |
| E9PK47 | Alpha-1,4 glucan phosphorylase | PYGL | 1 | 1 | 1,2 | 1,2 | 4 | + | + |  |  | + |  |
| E9PKB0 | Sortilin-related receptor | SORL1 | 1 | 1 | 3,5 | 3,5 | 1 |  |  | + |  | + | + |
| E9PP60 | GDP-L-fucose synthase | TSTA3 | 1 | 1 | 18,3 | 18,3 | 1 |  |  | + |  | + | + |
| E9PKZ0 | 60S ribosomal protein L8 | RPL8 | 4 | 4 | 12,7 | 12,7 | 36 | + | + | + |  | + |  |
| E9PPU1 | 40S ribosomal protein S3 | RPS3 | 5 | 5 | 38 | 38 | 15 | + | + | + |  | + | + |
| E9PNR2 | Ras and Rab interactor 1 | RIN1 | 1 | 1 | 2,6 | 2,6 | 1 |  |  | + |  | + |  |
| E9PNW4 | CD59 glycoprotein | CD59 | 2 | 2 | 22,2 | 22,2 | 4 |  |  | + | + | + | + |
| E9PP76 | Superoxide dismutase [Cu-Zn] | CCS | 3 | 3 | 50 | 50 | 4 | + |  |  | + | + | + |
| E9PPM7 | SPOC domain-containing protein 1 | SPOCD1 | 1 | 1 | 1,6 | 1,6 | 5 | + | + | + |  |  |  |
| F2Z2K0 | NSFL1 cofactor p47 | NSFL1C | 1 | 1 | 5,5 | 5,5 | 2 | + |  |  |  |  |  |
| F2Z2K5 | Janus kinase and microtubule-interacting protein 1 | JAKMIP1 | 1 | 1 | 3,5 | 3,5 | 1 | + |  |  |  | + |  |
| K7EMY3 | Chromodomain-helicase-DNA-binding protein 5 | CHD5 | 1 | 1 | 0,8 | 0,8 | 1 |  |  | + |  |  |  |
| F2Z2W8 | Selenium-binding protein 1 | SELENBP1 | 5 | 5 | 43,1 | 43,1 | 13 | + | + |  |  | + | + |
| F2Z2Y4 | Pyridoxal kinase | PDXK | 2 | 2 | 13,6 | 13,6 | 2 | + |  |  |  | + | + |
| F8VQ14 | T-complex protein 1 subunit beta | CCT2 | 1 | 1 | 6 | 6 | 1 |  |  | + |  | + |  |
| F5GXY9 | Low affinity immunoglobulin gamma Fc region receptor II-a | FCGR2A | 1 | 1 | 18,4 | 18,4 | 1 | + |  |  | + | + | + |
| F5GY16 |  | INPPL1 | 1 | 1 | 8,7 | 8,7 | 1 |  |  | + |  | + | + |
| K7ES92 | Minor histocompatibility protein HA-1 | HMHA1 | 1 | 1 | 2,3 | 2,3 | 1 |  | + |  |  |  |  |
| H0YGC7 | Acyl-CoA synthetase family member 3, mitochondrial | ACSF3 | 1 | 1 | 15,8 | 15,8 | 1 |  |  | + |  |  |  |
| F5H4C6 | N-acetylglucosamine-6-sulfatase | GNS | 2 | 2 | 11,3 | 11,3 | 5 | + |  | + |  | + | + |
| F5H6E2 | Unconventional myosin-Ic | MYO1C | 1 | 1 | 1,3 | 1,3 | 2 |  |  | + |  |  |  |
| F5H6P7 | Protein mago nashi homolog | MAGOHB | 1 | 1 | 10,8 | 10,8 | 2 |  | + | + | + | + |  |
| F5H6X6 | Neutral alpha-glucosidase AB | GANAB | 1 | 1 | 2,5 | 2,5 | 2 | + |  |  |  |  |  |
| F6RGN5 |  | SLC25A10 | 1 | 1 | 2 | 2 | 1 |  |  | + | + | + | + |
| F6TLX2 | Glyoxalase domain-containing protein 4 | GLOD4 | 5 | 5 | 16,7 | 16,7 | 6 | + | + | + |  | + |  |
| F8VWG6 | Liprin-alpha-2 | PPFIA2 | 1 | 1 | 17,3 | 17,3 | 1 | + |  |  |  |  |  |
| F8W0G4 | Poly(rC)-binding protein 2 | PCBP2 | 3 | 1 | 20,9 | 8,2 | 5 | + | + | + |  | + |  |
| F8W6I7 | Heterogeneous nuclear ribonucleoprotein A1 | HNRNPA1 | 5 | 5 | 20,8 | 20,8 | 24 | + | + | + | + | + | + |
| F8W6P5 |  | HBB | 18 | 2 | 100 | 26,7 | 7 | + | + | + |  | + | + |
| H0Y7J9 | Serine/threonine-protein kinase WNK2 | WNK2 | 1 | 1 | 1,7 | 1,7 | 0 |  |  | + |  |  |  |
| F8WCG7 | Phytanoyl-CoA dioxygenase domain-containing protein 1 | PHYHD1 | 1 | 1 | 23,9 | 23,9 | 3 | + | + |  |  | + |  |
| F8WE04 | Heat shock protein beta-1 | HSPB1 | 1 | 1 | 8,6 | 8,6 | 2 | + |  |  | + | + | + |
| F8WF40 |  | GLB1 | 1 | 1 | 48,8 | 48,8 | 1 |  |  | + | + | + | + |
| G3V1D3 | Dipeptidyl peptidase 3 | DPP3 | 5 | 5 | 12,6 | 12,6 | 8 | + | + |  |  |  | + |
| G3V1B3 | 60S ribosomal protein L21 | RPL21 | 2 | 2 | 17,2 | 17,2 | 3 | + | + |  |  |  |  |
| G3V1N2 |  | HBA2 | 15 | 1 | 98,2 | 7,3 | 21 | + | + | + |  |  |  |
| G3V1Q8 | Serine/threonine-protein kinase TAO3 | TAOK3 | 1 | 1 | 3,2 | 3,2 | 1 |  |  | + |  | + |  |
| G3V295 | Proteasome subunit alpha type | PSMA6 | 7 | 7 | 42,4 | 42,4 | 15 | + | + | + |  | + | + |
| G3V2B0 | MAGUK p55 subfamily member 5 | MPP5 | 1 | 1 | 10,5 | 10,5 | 1 |  |  | + |  | + |  |
| G3V3R5 | Brain-enriched guanylate kinase-associated protein | BEGAIN | 1 | 1 | 2,8 | 2,8 | 1 |  |  | + |  |  |  |
| G3V5U0 | MAM domain-containing glycosylphosphatidylinositol anchor protein 2 | MDGA2 | 1 | 1 | 7,7 | 7,7 | 1 |  |  | + | + | + | + |
| G3XAM7 | Catenin alpha-1 | CTNNA1 | 4 | 4 | 5,4 | 5,4 | 15 | + | + | + |  | + |  |
| G5E977 | Nicotinate phosphoribosyltransferase | NAPRT | 2 | 2 | 5,7 | 5,7 | 5 | + | + |  |  |  |  |
| G5E9X3 | Fibronectin type-III domain-containing protein 3A | FNDC3A | 2 | 2 | 4,5 | 4,5 | 2 |  |  | + |  | + |  |
| G5EA03 | LIM and calponin homology domains-containing protein 1 | LIMCH1 | 2 | 2 | 2 | 2 | 2 |  |  | + |  | + |  |
| H7C3D3 | Zyxin | ZYX | 2 | 2 | 15,9 | 15,9 | 11 | + | + |  |  | + |  |
| H0Y3C5 | Non-specific protein-tyrosine kinase | HCK | 1 | 1 | 2,1 | 2,1 | 11 | + | + | + |  | + | + |
| J3KSB5 | Neurofibromin | NF1 | 2 | 2 | 2,5 | 2,5 | 3 |  | + | + | + | + | + |
| H0Y512 | Adipocyte plasma membrane-associated protein | APMAP | 4 | 4 | 14,4 | 14,4 | 7 | + |  | + |  | + |  |
| H0Y7H7 | Dedicator of cytokinesis protein 4 | DOCK4 | 1 | 1 | 0,6 | 0,6 | 1 |  |  | + |  | + |  |
| H0Y5S6 | CTP synthase 2 | CTPS2 | 1 | 1 | 8,3 | 8,3 | 2 |  |  | + |  |  |  |
| H0Y6Z7 | Receptor-type tyrosine-protein phosphatase F | PTPRF | 6 | 6 | 6,3 | 6,3 | 31 | + | + | + |  | + |  |
| H0Y755 | Low affinity immunoglobulin gamma Fc region receptor III-A | FCGR3A | 5 | 2 | 16,6 | 7,7 | 2 | + | + | + | + | + | + |
| H3BLT5 | Uncharacterized protein KIAA1109 | KIAA1109 | 1 | 1 | 1,2 | 1,2 | 1 |  |  | + | + | + |  |
| H0Y7A7 | Calmodulin | CALM2 | 6 | 6 | 28,3 | 28,3 | 41 | + | + | + |  | + |  |
| H0Y7Z1 | Fibronectin | FN1 | 2 | 2 | 4,2 | 4,2 | 4 | + |  | + | + | + | + |
| H0Y8J2 | Centromere protein C | CENPC | 2 | 2 | 4,3 | 4,3 | 1 | + |  | + | + | + |  |
| H0YBS0 | Ankyrin-1 | ANK1 | 3 | 3 | 3,7 | 3,7 | 6 | + | + |  |  | + | + |
| H0YCS0 | Beta-2-syntrophin | SNTB2 | 1 | 1 | 6,2 | 6,2 | 1 |  |  | + |  |  |  |
| H0YD08 | Ubiquitin carboxyl-terminal hydrolase 33 | USP33 | 1 | 1 | 14,4 | 14,4 | 2 |  |  | + |  | + |  |
| H0YDC3 |  | AMPD3 | 1 | 1 | 24,5 | 24,5 | 1 |  |  | + |  | + | + |
| H0YEP5 | Sphingomyelin phosphodiesterase | SMPD1 | 1 | 1 | 6,6 | 6,6 | 1 |  |  | + |  | + | + |
| H0YIZ1 | Epididymal secretory protein E1 | NPC2 | 5 | 5 | 42,2 | 42,2 | 41 | + | + | + |  | + | + |
| H0YJT6 | Protein NRDE2 homolog | NRDE2 | 1 | 1 | 6,6 | 6,6 | 21 | + | + | + |  | + |  |
| H0YKU5 | COP9 signalosome complex subunit 2 | COPS2 | 2 | 2 | 13,3 | 13,3 | 2 |  |  | + |  | + |  |
| H0YLU2 | Proteasome activator complex subunit 1 | PSME1 | 1 | 1 | 16,7 | 16,7 | 2 | + | + |  | + | + |  |
| H0YLY0 | Serine/threonine-protein kinase MRCK beta | CDC42BPB | 1 | 1 | 2,7 | 2,7 | 1 |  |  | + |  |  |  |
| H0YND0 |  | FBN1 | 2 | 1 | 5 | 2,2 | 1 | + | + | + | + | + | + |
| H3BR01 | Kunitz-type protease inhibitor 1 | SPINT1 | 1 | 1 | 4,6 | 4,6 | 6 | + | + | + |  |  | + |
| H3BNU3 | Elongation factor Tu, mitochondrial | TUFM | 1 | 1 | 14,9 | 14,9 | 3 | + | + |  |  | + |  |
| H3BP66 | N-acetylgalactosamine-6-sulfatase | GALNS | 1 | 1 | 14,7 | 14,7 | 3 |  |  | + |  | + | + |
| H3BR20 | Alpha-1,6-mannosylglycoprotein 6-beta-N-acetylglucosaminyltransferase B | MGAT5B | 1 | 1 | 15,5 | 15,5 | 1 |  |  | + |  |  |  |
| H3BTN5 | Pyruvate kinase | PKM | 6 | 6 | 10,7 | 10,7 | 19 | + | + |  | + | + | + |
| H3BU51 | Neuroplastin | NPTN | 1 | 1 | 8,2 | 8,2 | 2 | + | + |  |  | + | + |
| H7BXD5 | Grancalcin | GCA | 5 | 5 | 33,3 | 33,3 | 24 | + | + | + |  | + | + |
| H7BXK7 | Serpin B8 | SERPINB8 | 2 | 1 | 13,2 | 7,9 | 1 | + | + | + | + | + | + |
| H7BZ98 | EH domain-binding protein 1 | EHBP1 | 2 | 2 | 9,9 | 9,9 | 1 |  |  | + |  |  |  |
| H7BZJ3 |  | PDIA3 | 4 | 1 | 41,5 | 11,4 | 10 | + | + |  |  | + | + |
| H7C1K5 | Actin-binding protein anillin | ANLN | 1 | 1 | 5,1 | 5,1 | 1 |  |  | + |  | + |  |
| H7C204 | Rabenosyn-5 | RBSN | 1 | 1 | 6,4 | 6,4 | 1 |  |  | + |  |  |  |
| H7C2H6 | Fibronectin type III domain-containing protein 7 | FNDC7 | 1 | 1 | 2,6 | 2,6 | 1 |  |  | + |  |  |  |
| H7C389 | WD repeat-containing protein 92 | WDR92 | 1 | 1 | 16,3 | 16,3 | 1 | + |  |  |  |  |  |
| H7C3U4 | E3 ubiquitin-protein ligase MYCBP2 | MYCBP2 | 1 | 1 | 0,8 | 0,8 | 5 | + | + |  |  | + |  |
| H7C5M3 |  | IFT80 | 1 | 1 | 36,4 | 36,4 | 0 |  |  | + |  | + |  |
| H7C5W8 |  | ACTN1 | 19 | 1 | 58,2 | 9,1 | 1 | + | + | + | + | + |  |
| I3L0N3 | Vesicle-fusing ATPase | NSF | 2 | 2 | 3,7 | 3,7 | 2 | + | + | + |  | + | + |
| I3L276 | Heme oxygenase 2 | HMOX2 | 1 | 1 | 14,5 | 14,5 | 4 | + | + | + |  |  | + |
| I3L3R1 | Homeobox protein Hox-B8 | HOXB8 | 1 | 1 | 5 | 5 | 1 |  |  | + |  |  |  |
| I6L8B7 | Fatty acid-binding protein, epidermal | FABP5 | 2 | 2 | 40,6 | 40,6 | 7 | + | + |  |  | + | + |
| J3KN29 | 26S proteasome non-ATPase regulatory subunit 9 | PSMD9 | 1 | 1 | 5,4 | 5,4 | 4 | + | + |  |  |  | + |
| J3KN67 | Tropomyosin alpha-3 chain | TPM3 | 9 | 6 | 29,1 | 18,6 | 57 | + | + | + |  | + |  |
| J3KNB4 | Cathelicidin antimicrobial peptide | CAMP | 9 | 9 | 38,7 | 38,7 | 286 | + | + | + | + | + | + |
| J3KNF5 | Centrosomal protein of 290 kDa | CEP290 | 1 | 1 | 0,7 | 0,7 | 1 |  |  | + |  |  |  |
| J3KNK7 |  | PDXDC1 | 1 | 1 | 3,5 | 3,5 | 1 |  | + |  |  |  |  |
| J3KPA1 | Cysteine-rich secretory protein 3 | CRISP3 | 2 | 2 | 8 | 8 | 17 | + | + | + |  | + | + |
| J3KQN7 | Interleukin-17 receptor E | IL17RE | 1 | 1 | 4,6 | 4,6 | 1 |  |  | + | + | + |  |
| M0R1R2 | Zinc finger protein 418 | ZNF418 | 1 | 1 | 16,1 | 16,1 | 0 |  |  | + |  |  |  |
| J3KRA9 | Serine/threonine-protein kinase SMG1 | SMG1 | 1 | 1 | 0,3 | 0,3 | 1 |  |  | + |  | + |  |
| J3KRI1 |  | SLC39A11 | 1 | 1 | 96,2 | 96,2 | 1 |  |  | + |  | + |  |
| J3QR00 | Trafficking protein particle complex subunit 8 | TRAPPC8 | 1 | 1 | 13,9 | 13,9 | 1 |  |  | + |  | + |  |
| J3QLI9 | Small nuclear ribonucleoprotein Sm D1 | SNRPD1 | 1 | 1 | 26,7 | 26,7 | 1 |  |  | + | + | + |  |
| J3QLM5 |  | KIAA0100 | 1 | 1 | 12,8 | 12,8 | 1 |  |  | + |  |  |  |
| J3QQJ4 | Carboxypeptidase D | CPD | 1 | 1 | 15,2 | 15,2 | 1 |  |  | + | + | + | + |
| J3QRP6 | Na(+)/H(+) exchange regulatory cofactor NHE-RF1 | SLC9A3R1 | 2 | 2 | 9,8 | 9,8 | 20 | + | + | + |  | + | + |
| J3QRS3 | Myosin regulatory light chain 12A | MYL12A | 3 | 3 | 15,8 | 15,8 | 3 |  | + |  |  | + | + |
| J3QSA3 |  | UBB | 5 | 1 | 86 | 32,6 | 17 | + | + | + |  | + | + |
| J3QTJ6 | Fibrous sheath-interacting protein 2 | FSIP2 | 4 | 4 | 0,9 | 0,9 | 4 | + |  | + |  |  |  |
| J9JID7 | Lamin-B2 | LMNB2 | 2 | 2 | 4,2 | 4,2 | 3 | + | + |  |  | + |  |
| K7EJ01 |  | AP2B1 | 1 | 1 | 12,2 | 12,2 | 1 |  |  | + |  |  |  |
| K7EJC1 | 26S proteasome non-ATPase regulatory subunit 8 | PSMD8 | 1 | 1 | 9,3 | 9,3 | 2 |  |  | + |  | + |  |
| K7EJZ9 | Microtubule-associated serine/threonine-protein kinase 1 | MAST1 | 1 | 1 | 7,4 | 7,4 | 1 |  |  | + |  |  |  |
| K7EKG2 | Thioredoxin-like protein 1 | TXNL1 | 1 | 1 | 81,5 | 81,5 | 1 | + |  |  |  | + | + |
| K7EQ37 | Protein unc-13 homolog D | UNC13D | 2 | 2 | 10,1 | 10,1 | 3 | + | + |  |  | + | + |
| K7ENJ9 | Rab GTPase-binding effector protein 1 | RABEP1 | 1 | 1 | 11,8 | 11,8 | 2 |  |  | + | + | + |  |
| K7ENX9 |  | ZNF570 | 1 | 1 | 38,6 | 38,6 | 1 |  |  | + |  |  |  |
| K7EPW0 | Spindle and kinetochore-associated protein 1 | SKA1 | 1 | 1 | 12,1 | 12,1 | 1 |  |  | + |  |  |  |
| K7EQT1 | Fizzy-related protein homolog | FZR1 | 1 | 1 | 3,3 | 3,3 | 2 | + | + |  |  |  |  |
| K7ERG4 | Small nuclear ribonucleoprotein Sm D2 | SNRPD2 | 2 | 2 | 37,2 | 37,2 | 3 | + | + | + |  | + |  |
| K7ES95 | Protein LZIC | LZIC | 1 | 1 | 9,5 | 9,5 | 1 | + |  |  |  |  |  |
| Q3HM38 | Programmed cell death protein 5 | PDCD5 | 1 | 1 | 32,5 | 32,5 | 1 | + |  |  |  | + | + |
| M0QXF7 | Myeloid-derived growth factor | MYDGF | 4 | 4 | 48,8 | 48,8 | 16 | + | + | + |  |  |  |
| M0R024 |  | ZNF738 | 1 | 1 | 14,7 | 14,7 | 0 |  |  | + |  | + |  |
| M0R0S5 | Mast cell-expressed membrane protein 1 | MCEMP1 | 3 | 3 | 30,6 | 30,6 | 6 | + |  | + |  | + |  |
| M0R131 | Dual specificity tyrosine-phosphorylation-regulated kinase 1B | DYRK1B | 1 | 1 | 7,6 | 7,6 | 1 |  |  | + |  |  |  |
| O00254-2 |  |  | 1 | 1 | 2,8 | 2,8 | 16 | + | + | + |  |  |  |
| O00299 | Chloride intracellular channel protein 1 | CLIC1 | 3 | 3 | 16,2 | 16,2 | 8 | + | + |  |  | + |  |
| O00370 | LINE-1 retrotransposable element ORF2 protein | | 1 | 1 | 1 | 1 | 1 |  |  | + |  |  |  |
| O00421 | C-C chemokine receptor-like 2 | CCRL2 | 1 | 1 | 6,1 | 6,1 | 1 |  |  | + |  | + | + |
| O00443 | Phosphatidylinositol 4-phosphate 3-kinase C2 domain-containing subunit alpha | PIK3C2A | 1 | 1 | 1,6 | 1,6 | 1 |  |  | + |  |  |  |
| O00560-2 | Syntenin-1 | SDCBP | 4 | 4 | 22,2 | 22,2 | 23 | + | + | + |  | + | + |
| O14793 | Growth/differentiation factor 8 | MSTN | 1 | 1 | 3,2 | 3,2 | 1 |  |  | + |  | + | + |
| O14818 | Proteasome subunit alpha type-7 | PSMA7 | 6 | 6 | 39,1 | 39,1 | 24 | + | + | + | + | + | + |
| O15143 | Actin-related protein 2/3 complex subunit 1B | ARPC1B | 3 | 3 | 8,6 | 8,6 | 4 | + | + |  |  | + |  |
| O15400-2 | Syntaxin-7 | STX7 | 5 | 5 | 23,4 | 23,4 | 11 | + | + |  |  |  |  |
| O43593-2 | Lysine-specific demethylase hairless | HR | 1 | 1 | 1,9 | 1,9 | 1 |  |  | + |  |  |  |
| O43707 | Alpha-actinin-4 | ACTN4 | 44 | 26 | 51,6 | 34,5 | 136 | + | + | + | + | + | + |
| O75083 | WD repeat-containing protein 1 | WDR1 | 11 | 11 | 26,2 | 26,2 | 95 | + | + | + |  | + |  |
| O75223 | Gamma-glutamylcyclotransferase | GGCT | 4 | 4 | 19,7 | 19,7 | 30 | + | + | + |  |  | + |
| O75368 | SH3 domain-binding glutamic acid-rich-like protein | SH3BGRL | 7 | 7 | 59,6 | 59,6 | 62 | + | + | + |  | + |  |
| O75531 | Barrier-to-autointegration factor | BANF1 | 3 | 3 | 30,3 | 30,3 | 6 | + |  | + |  | + | + |
| O75533 | Splicing factor 3B subunit 1 | SF3B1 | 1 | 1 | 1,4 | 1,4 | 2 | + |  |  |  | + |  |
| O75594 | Peptidoglycan recognition protein 1 | PGLYRP1 | 3 | 3 | 27,6 | 27,6 | 63 | + | + | + |  | + | + |
| O75822-2 | Eukaryotic translation initiation factor 3 subunit J | EIF3J | 3 | 3 | 9,8 | 9,8 | 29 | + | + | + |  |  |  |
| O75955-2 | Flotillin-1 | FLOT1 | 2 | 2 | 8,4 | 8,4 | 4 | + |  | + |  |  | + |
| O94875-7 | Sorbin and SH3 domain-containing protein 2 | SORBS2 | 2 | 2 | 4,3 | 4,3 | 2 |  |  | + |  | + |  |
| P00338 | L-lactate dehydrogenase A chain | LDHA | 10 | 9 | 40,4 | 36,7 | 26 | + | + | + |  |  | + |
| P00390-2 | Glutathione reductase, mitochondrial | GSR | 12 | 12 | 39,9 | 39,9 | 40 | + | + | + |  | + | + |
| P00441 | Superoxide dismutase [Cu-Zn] | SOD1 | 12 | 12 | 87,7 | 87,7 | 70 | + | + | + | + | + | + |
| P00491 | Purine nucleoside phosphorylase | PNP | 3 | 3 | 17,3 | 17,3 | 7 | + | + |  | + | + | + |
| P00492 | Hypoxanthine-guanine phosphoribosyltransferase | HPRT1 | 3 | 3 | 26,6 | 26,6 | 3 | + |  | + | + | + | + |
| P00558 | Phosphoglycerate kinase 1 | PGK1 | 31 | 31 | 76,5 | 76,5 | 103 | + | + | + | + | + | + |
| P00738 | Haptoglobin | HP | 21 | 21 | 51,7 | 51,7 | 210 | + | + | + | + | + | + |
| P00918 | Carbonic anhydrase 2 | CA2 | 25 | 25 | 70,4 | 70,4 | 119 | + | + | + | + | + | + |
| P01034 | Cystatin-C | CST3 | 2 | 2 | 18,5 | 18,5 | 3 |  | + |  | + | + | + |
| P01040 | Cystatin-A | CSTA | 4 | 4 | 65,3 | 65,3 | 14 | + | + | + |  | + | + |
| P02042 | Hemoglobin subunit delta | HBD | 25 | 6 | 97,3 | 54,4 | 551 | + | + | + | + | + | + |
| P02100 | Hemoglobin subunit epsilon | HBE1 | 3 | 1 | 18,4 | 5,4 | 2 | + | + | + |  | + | + |
| P02730-2 | Band 3 anion transport protein | SLC4A1 | 7 | 7 | 11,8 | 11,8 | 9 | + |  | + | + | + | + |
| P02763 | Alpha-1-acid glycoprotein 1 | ORM1 | 4 | 4 | 20,4 | 20,4 | 14 | + | + | + |  | + | + |
| P02788 | Lactotransferrin | LTF | 103 | 99 | 89,3 | 88,2 | #### | + | + | + | + | + | + |
| P02792 | Ferritin light chain | FTL | 3 | 3 | 22,9 | 22,9 | 15 | + | + | + | + | + | + |
| P02795 | Metallothionein-2 | MT2A | 5 | 3 | 50,8 | 29,5 | 142 | + | + | + | + | + | + |
| P04040 | Catalase | CAT | 34 | 34 | 58,8 | 58,8 | 559 | + | + | + | + | + | + |
| P04075 | Fructose-bisphosphate aldolase A | ALDOA | 30 | 24 | 73,6 | 61,5 | 478 | + | + | + |  | + |  |
| P04080 | Cystatin-B | CSTB | 2 | 2 | 24,5 | 24,5 | 22 | + | + | + |  | + | + |
| P04083 | Annexin A1 | ANXA1 | 27 | 27 | 69,4 | 69,4 | 319 | + | + | + | + | + | + |
| P04150-7 |  |  | 1 | 1 | 2,9 | 2,9 | 1 |  |  | + |  |  |  |
| P04259 | Keratin, type II cytoskeletal 6B | KRT6B | 30 | 1 | 37,8 | 1,6 | 69 | + | + | + |  | + |  |
| P04264 | Keratin, type II cytoskeletal 1 | KRT1 | 56 | 42 | 56,2 | 50,2 | 865 | + | + | + |  | + | + |
| P04406 | Glyceraldehyde-3-phosphate dehydrogenase | GAPDH | 12 | 12 | 46,3 | 46,3 | 73 | + | + | + | + | + | + |
| P04839 | Cytochrome b-245 heavy chain | CYBB | 4 | 4 | 10 | 10 | 7 | + | + | + | + | + | + |
| P04899 | Guanine nucleotide-binding protein G(i) subunit alpha-2 | GNAI2 | 5 | 5 | 23,9 | 23,9 | 14 | + |  | + |  | + |  |
| P05109 | Protein S100-A8 | S100A8 | 21 | 21 | 100 | 100 | 2073 | + | + | + | + | + | + |
| P05114 | Non-histone chromosomal protein HMG-14 | HMGN1 | 4 | 4 | 42 | 42 | 34 | + | + | + |  |  | + |
| P05164-2 | Myeloperoxidase | MPO | 56 | 49 | 70,9 | 63,4 | 3625 | + | + | + | + | + | + |
| P05204 | Non-histone chromosomal protein HMG-17 | HMGN2 | 7 | 7 | 36,7 | 36,7 | 183 | + | + | + |  |  | + |
| P06396-2 | Gelsolin | GSN | 22 | 17 | 28,2 | 21,8 | 124 | + | + | + | + | + | + |
| P06400 | Retinoblastoma-associated protein | RB1 | 1 | 1 | 2,7 | 2,7 | 1 |  |  | + |  | + | + |
| P06702 | Protein S100-A9 | S100A9 | 22 | 22 | 99,1 | 99,1 | 3958 | + | + | + | + | + | + |
| R4GN98 | Protein S100 | S100A6 | 5 | 5 | 48,2 | 48,2 | 43 | + | + | + | + | + | + |
| P06733 | Alpha-enolase | ENO1 | 15 | 4 | 37,8 | 9,9 | 47 | + | + | + | + | + | + |
| P06733-2 | Alpha-enolase | ENO1 | 13 | 2 | 38,4 | 2,9 | 16 | + | + | + | + | + | + |
| P06744 | Glucose-6-phosphate isomerase | GPI | 22 | 22 | 48,4 | 48,4 | 133 | + | + | + | + | + | + |
| P06748-3 | Nucleophosmin | NPM1 | 5 | 5 | 29 | 29 | 34 | + | + | + | + | + | + |
| Q16778 | Histone H2B type 2-E | HIST2H2BE | 6 | 1 | 43,7 | 7,9 | 2 | + | + | + |  | + |  |
| P07108 | Acyl-CoA-binding protein | DBI | 6 | 6 | 86,2 | 86,2 | 27 | + | + | + |  | + | + |
| P07195 | L-lactate dehydrogenase B chain | LDHB | 4 | 3 | 20,1 | 16,5 | 3 | + |  | + |  | + | + |
| P07237 | Protein disulfide-isomerase | P4HB | 5 | 5 | 10,2 | 10,2 | 22 | + | + | + |  | + | + |
| P07305 | Histone H1.0 | H1F0 | 1 | 1 | 6,7 | 6,7 | 14 | + | + | + |  | + |  |
| P07311 | Acylphosphatase-1 | ACYP1 | 2 | 2 | 30,3 | 30,3 | 8 | + | + | + |  | + |  |
| P07355 | Annexin A2 | ANXA2 | 5 | 5 | 23,3 | 23,3 | 21 | + | + | + | + | + | + |
| P07451 | Carbonic anhydrase 3 | CA3 | 1 | 1 | 8,1 | 8,1 | 4 | + | + |  | + | + | + |
| P07737 | Profilin-1 | PFN1 | 17 | 17 | 95 | 95 | 454 | + | + | + |  | + | + |
| P07738 | Bisphosphoglycerate mutase | BPGM | 3 | 3 | 8,1 | 8,1 | 4 | + |  |  |  |  | + |
| P07954-2 | Fumarate hydratase, mitochondrial | FH | 1 | 1 | 4,9 | 4,9 | 1 |  |  | + |  |  |  |
| P07996-2 | Thrombospondin-1 | THBS1 | 6 | 6 | 8,2 | 8,2 | 11 |  |  | + | + | + | + |
| P08133-2 | Annexin A6 | ANXA6 | 28 | 28 | 46,2 | 46,2 | 226 | + | + | + | + | + | + |
| P08238 | Heat shock protein HSP 90-beta | HSP90AB1 | 6 | 4 | 6,1 | 3,7 | 26 | + | + | + | + | + | + |
| P08246 | Neutrophil elastase | ELANE | 7 | 7 | 46,8 | 46,8 | 294 | + | + | + | + | + | + |
| P08311 | Cathepsin G | CTSG | 19 | 19 | 55,7 | 55,7 | 954 | + | + | + | + | + | + |
| P08567 | Pleckstrin | PLEK | 3 | 3 | 10,9 | 10,9 | 15 | + | + | + |  | + | + |
| P08670 | Vimentin | VIM | 13 | 10 | 26,6 | 21,5 | 50 | + | + | + | + | + | + |
| P08758 | Annexin A5 | ANXA5 | 11 | 11 | 33,8 | 33,8 | 44 | + | + | + | + | + | + |
| P09211 | Glutathione S-transferase P | GSTP1 | 2 | 2 | 17,1 | 17,1 | 4 | + |  | + | + | + | + |
| P09467 | Fructose-1,6-bisphosphatase 1 | FBP1 | 3 | 3 | 8,6 | 8,6 | 10 | + | + | + |  | + |  |
| P09525 | Annexin A4 | ANXA4 | 11 | 11 | 43,6 | 43,6 | 65 | + | + | + | + | + | + |
| P09960-2 | Leukotriene A-4 hydrolase | LTA4H | 4 | 4 | 10,2 | 10,2 | 8 | + | + | + |  | + | + |
| P09972 | Fructose-bisphosphate aldolase C | ALDOC | 9 | 4 | 26,6 | 14,8 | 66 | + | + | + |  | + |  |
| P0CF75 | Endogenous Bornavirus-like nucleoprotein 1 | EBLN1 | 1 | 1 | 4,9 | 4,9 | 1 | + |  |  |  |  |  |
| P10153 | Non-secretory ribonuclease | RNASE2 | 7 | 7 | 23 | 23 | 93 | + | + | + | + | + | + |
| P10412 | Histone H1.4 | HIST1H1E | 3 | 3 | 9,6 | 9,6 | 12 | + | + |  |  | + |  |
| P10599 | Thioredoxin | TXN | 10 | 10 | 73,3 | 73,3 | 227 | + | + | + | + | + | + |
| P10768 | S-formylglutathione hydrolase | ESD | 5 | 5 | 27 | 27 | 12 | + | + | + | + | + | + |
| P11021 | 78 kDa glucose-regulated protein | HSPA5 | 21 | 20 | 34,1 | 34,1 | 115 | + | + | + | + | + | + |
| P11137-3 | Microtubule-associated protein 2 | MAP2 | 4 | 4 | 2,3 | 2,3 | 8 | + | + | + | + | + | + |
| P11142 | Heat shock cognate 71 kDa protein | HSPA8 | 32 | 27 | 52,9 | 46,1 | 382 | + | + | + | + | + | + |
| Q4VB86 | Protein 4.1 | EPB41 | 2 | 2 | 5,1 | 5,1 | 4 |  |  | + |  | + | + |
| P11215 | Integrin alpha-M | ITGAM | 12 | 12 | 12 | 12 | 41 | + | + | + | + | + | + |
| P11277-3 | Spectrin beta chain, erythrocytic | SPTB | 5 | 4 | 2,9 | 2,5 | 8 | + | + |  |  |  | + |
| P11279-2 | Lysosome-associated membrane glycoprotein 1 | LAMP1 | 3 | 3 | 11 | 11 | 10 | + | + | + | + | + | + |
| P11532-4 | Dystrophin | DMD | 3 | 3 | 0,5 | 0,5 | 2 | + |  | + |  | + | + |
| P11678 | Eosinophil peroxidase | EPX | 13 | 6 | 17,6 | 9,7 | 12 | + | + | + |  | + | + |
| P12270 | Nucleoprotein TPR | TPR | 2 | 2 | 1,1 | 1,1 | 2 | + |  | + |  | + |  |
| P12429 | Annexin A3 | ANXA3 | 36 | 26 | 74,9 | 51,7 | 502 | + | + | + | + | + | + |
| P12724 | Eosinophil cationic protein | RNASE3 | 8 | 8 | 42,5 | 42,5 | 117 | + | + | + | + | + | + |
| P12814 | Alpha-actinin-1 | ACTN1 | 49 | 18 | 52,6 | 24,1 | 388 | + | + | + | + | + |  |
| P13688-2 | Carcinoembryonic antigen-related cell adhesion molecule 1 | CEACAM1 | 1 | 1 | 4,1 | 4,1 | 16 | + | + | + |  | + | + |
| P13796 | Plastin-2 | LCP1 | 39 | 37 | 59,3 | 57,4 | 583 | + | + | + |  | + | + |
| P14174 | Macrophage migration inhibitory factor | MIF | 2 | 2 | 17,4 | 17,4 | 19 | + | + | + | + | + | + |
| P14543-2 | Nidogen-1 | NID1 | 2 | 2 | 0,9 | 0,9 | 9 | + | + | + | + | + | + |
| P14780 | Matrix metalloproteinase-9 | MMP9 | 10 | 10 | 13,6 | 13,6 | 59 | + | + | + | + | + | + |
| P15144 | Aminopeptidase N | ANPEP | 10 | 10 | 12,7 | 12,7 | 20 | + |  | + | + | + | + |
| P15248 | Interleukin-9 | IL9 | 1 | 1 | 14,6 | 14,6 | 1 |  |  | + | + | + | + |
| P16144-4 | Integrin beta-4 | ITGB4 | 1 | 1 | 0,6 | 0,6 | 6 | + | + |  |  | + | + |
| P16401 | Histone H1.5 | HIST1H1B | 3 | 3 | 13,7 | 13,7 | 5 | + | + | + |  |  |  |
| P16402 | Histone H1.3 | HIST1H1D | 3 | 3 | 13,6 | 13,6 | 4 | + | + | + |  | + |  |
| P16403 | Histone H1.2 | HIST1H1C | 1 | 1 | 9,9 | 9,9 | 1 |  | + |  |  | + |  |
| P16949 | Stathmin | STMN1 | 5 | 5 | 25,5 | 25,5 | 18 | + | + | + |  | + | + |
| P17066 | Heat shock 70 kDa protein 6 | HSPA6 | 9 | 2 | 13,8 | 3,7 | 37 | + | + | + |  | + |  |
| P17174 | Aspartate aminotransferase, cytoplasmic | GOT1 | 3 | 3 | 12,8 | 12,8 | 6 | + |  | + |  | + | + |
| P17931 | Galectin-3 | LGALS3 | 6 | 6 | 23,6 | 23,6 | 21 | + | + | + | + | + | + |
| P18206-2 | Vinculin | VCL | 24 | 24 | 24,7 | 24,7 | 153 | + | + | + |  | + | + |
| P18669 | Phosphoglycerate mutase 1 | PGAM1 | 10 | 10 | 35,8 | 35,8 | 57 | + | + | + |  | + |  |
| P19022-2 | Cadherin-2 | CDH2 | 1 | 1 | 1,3 | 1,3 | 1 | + |  | + |  | + | + |
| P19338 | Nucleolin | NCL | 4 | 4 | 7,7 | 7,7 | 5 | + |  |  | + | + | + |
| P19878-2 | Neutrophil cytosol factor 2 | NCF2 | 2 | 2 | 3,3 | 3,3 | 6 | + | + | + | + | + | + |
| P20073-2 | Annexin A7 | ANXA7 | 1 | 1 | 3,4 | 3,4 | 2 | + |  |  |  | + | + |
| P20160 | Azurocidin | AZU1 | 12 | 9 | 68,9 | 43,8 | 452 | + | + | + |  | + | + |
| P20618 | Proteasome subunit beta type-1 | PSMB1 | 2 | 2 | 8,7 | 8,7 | 3 | + |  | + |  | + |  |
| P20700 | Lamin-B1 | LMNB1 | 7 | 7 | 9,2 | 9,2 | 30 | + | + | + | + | + |  |
| P21333-2 | Filamin-A | FLNA | 95 | 1 | 45,8 | 0,6 | 780 | + | + | + |  | + | + |
| P21709-3 | Ephrin type-A receptor 1 | EPHA1 | 1 | 1 | 4,4 | 4,4 | 2 |  |  | + |  | + | + |
| Q32Q12 | Nucleoside diphosphate kinase | NME1-NME2 | 7 | 7 | 45,5 | 45,5 | 34 | + | + | + |  |  |  |
| P22748 | Carbonic anhydrase 4 | CA4 | 2 | 2 | 9 | 9 | 8 | + | + | + | + | + | + |
| P23284 | Peptidyl-prolyl cis-trans isomerase B | PPIB | 9 | 9 | 46,3 | 46,3 | 61 | + | + | + |  | + |  |
| P25774-2 | Cathepsin S | CTSS | 5 | 5 | 21,4 | 21,4 | 31 | + | + | + | + | + | + |
| P25786 | Proteasome subunit alpha type-1 | PSMA1 | 4 | 4 | 23,6 | 23,6 | 13 | + |  | + |  | + | + |
| P25815 | Protein S100-P | S100P | 6 | 6 | 58,9 | 58,9 | 83 | + | + | + |  | + | + |
| P26022 | Pentraxin-related protein PTX3 | PTX3 | 1 | 1 | 4,2 | 4,2 | 1 | + |  |  |  | + | + |
| P26038 | Moesin | MSN | 9 | 9 | 9,9 | 9,9 | 59 | + | + | + | + | + | + |
| P26447 | Protein S100-A4 | S100A4 | 3 | 3 | 28,7 | 28,7 | 19 | + | + |  |  | + | + |
| P27105 | Erythrocyte band 7 integral membrane protein | STOM | 10 | 10 | 38,5 | 38,5 | 97 | + | + | + |  | + |  |
| P27695 | DNA-(apurinic or apyrimidinic site) lyase | APEX1 | 12 | 12 | 56,3 | 56,3 | 48 | + | + | + | + | + | + |
| P27797 | Calreticulin | CALR | 6 | 6 | 21,1 | 21,1 | 19 | + | + | + | + | + | + |
| P27824-3 | Calnexin | CANX | 2 | 2 | 7,2 | 7,2 | 20 | + | + | + | + | + | + |
| Q5JNW7 | Proteasome subunit beta type | PSMB8 | 2 | 2 | 12,7 | 12,7 | 7 | + | + | + | + | + | + |
| P28066 | Proteasome subunit alpha type-5 | PSMA5 | 3 | 3 | 17 | 17 | 20 | + |  | + |  | + |  |
| P28799 | Granulins | GRN | 7 | 7 | 13 | 13 | 106 | + | + | + | + | + | + |
| P29350-2 | Tyrosine-protein phosphatase non-receptor type 6 | PTPN6 | 3 | 3 | 7,2 | 7,2 | 7 | + | + |  | + | + | + |
| P29401 | Transketolase | TKT | 32 | 32 | 53,9 | 53,9 | 270 | + | + | + |  | + | + |
| P30041 | Peroxiredoxin-6 | PRDX6 | 3 | 3 | 19,2 | 19,2 | 5 | + | + |  |  | + | + |
| P30043 | Flavin reductase (NADPH) | BLVRB | 9 | 9 | 66 | 66 | 17 | + | + |  |  | + | + |
| P30086 | Phosphatidylethanolamine-binding protein 1 | PEBP1 | 12 | 12 | 81,3 | 81,3 | 38 | + | + |  |  | + | + |
| P30101 | Protein disulfide-isomerase A3 | PDIA3 | 10 | 7 | 29,9 | 22,6 | 20 | + | + | + |  | + | + |
| P30490 | HLA class I histocompatibility antigen, B-52 alpha chain | HLA-B | 3 | 2 | 16 | 12,2 | 6 | + |  | + |  | + |  |
| P30740 | Leukocyte elastase inhibitor | SERPINB1 | 27 | 26 | 64,6 | 62 | 652 | + | + | + |  | + | + |
| Q5VSP4 | Putative lipocalin 1-like protein 1 | LCN1P1 | 1 | 1 | 6,8 | 6,8 | 3 | + |  | + |  |  |  |
| P31146 | Coronin-1A | CORO1A | 21 | 21 | 44,5 | 44,5 | 178 | + | + | + | + | + |  |
| P31150 | Rab GDP dissociation inhibitor alpha | GDI1 | 10 | 4 | 33,1 | 17,2 | 11 | + | + | + |  | + | + |
| P31151 | Protein S100-A7 | S100A7 | 2 | 2 | 22,8 | 22,8 | 3 | + |  | + |  |  | + |
| P31944 | Caspase-14 | CASP14 | 4 | 4 | 18,2 | 18,2 | 12 | + |  |  |  |  | + |
| P31949 | Protein S100-A11 | S100A11 | 8 | 8 | 56,2 | 56,2 | 75 | + | + | + |  | + | + |
| P32119 | Peroxiredoxin-2 | PRDX2 | 14 | 13 | 56,6 | 51 | 50 | + | + | + | + | + | + |
| P32320 | Cytidine deaminase | CDA | 5 | 5 | 48,6 | 48,6 | 67 | + | + | + | + | + | + |
| P32942 | Intercellular adhesion molecule 3 | ICAM3 | 3 | 3 | 8 | 8 | 5 | + |  | + |  | + | + |
| P34910 | Protein EVI2B | EVI2B | 1 | 1 | 3,3 | 3,3 | 2 |  |  | + |  | + |  |
| P34947 | G protein-coupled receptor kinase 5 | GRK5 | 2 | 2 | 4,7 | 4,7 | 1 |  |  | + |  | + | + |
| P34969-3 | 5-hydroxytryptamine receptor 7 | HTR7 | 1 | 1 | 3 | 3 | 1 |  |  | + |  | + | + |
| P35237 | Serpin B6 | SERPINB6 | 9 | 9 | 33,8 | 33,8 | 14 | + | + | + | + | + | + |
| P35527 | Keratin, type I cytoskeletal 9 | KRT9 | 43 | 42 | 66,9 | 66,9 | 813 | + | + | + |  |  | + |
| P35555 | Fibrillin-1 | FBN1 | 14 | 13 | 5,5 | 4,7 | 44 | + | + | + | + | + | + |
| P35579 | Myosin-9 | MYH9 | 73 | 71 | 32 | 31,5 | 401 | + | + | + | + | + | + |
| P35754 | Glutaredoxin-1 | GLRX | 2 | 2 | 34,9 | 34,9 | 3 | + |  |  |  | + | + |
| P36222 | Chitinase-3-like protein 1 | CHI3L1 | 4 | 4 | 12,8 | 12,8 | 9 | + | + | + | + | + | + |
| P36507 | Dual specificity mitogen-activated protein kinase kinase 2 | MAP2K2 | 1 | 1 | 3,2 | 3,2 | 3 | + |  | + |  |  | + |
| Q86SW4 | Dihydrolipoyllysine-residue succinyltransferase component of 2-oxoglutarate dehydrogenase complex, mitochondrial | DLST | 3 | 3 | 12,9 | 12,9 | 9 | + | + | + |  | + |  |
| P37802 | Transgelin-2 | TAGLN2 | 7 | 7 | 49,2 | 49,2 | 36 | + | + | + |  | + | + |
| P37837 | Transaldolase | TALDO1 | 26 | 26 | 54,3 | 54,3 | 299 | + | + | + | + | + | + |
| P39687 | Acidic leucine-rich nuclear phosphoprotein 32 family member A | ANP32A | 4 | 4 | 25,7 | 25,7 | 7 | + | + |  |  | + |  |
| P40121 | Macrophage-capping protein | CAPG | 10 | 10 | 31,3 | 31,3 | 47 | + | + |  |  | + | + |
| P40199 | Carcinoembryonic antigen-related cell adhesion molecule 6 | CEACAM6 | 2 | 2 | 14 | 14 | 3 | + |  | + |  | + | + |
| P40818-2 | Ubiquitin carboxyl-terminal hydrolase 8 | USP8 | 1 | 1 | 2,2 | 2,2 | 1 |  |  | + |  |  | + |
| P40925 | Malate dehydrogenase, cytoplasmic | MDH1 | 11 | 11 | 43,1 | 43,1 | 48 | + | + | + |  | + |  |
| P40926-2 | Malate dehydrogenase, mitochondrial | MDH2 | 4 | 4 | 28,4 | 28,4 | 10 | + |  | + |  | + |  |
| P41218 | Myeloid cell nuclear differentiation antigen | MNDA | 22 | 22 | 43,7 | 43,7 | 219 | + | + | + | + | + | + |
| P41222 | Prostaglandin-H2 D-isomerase | PTGDS | 1 | 1 | 8,9 | 8,9 | 1 |  | + |  |  | + | + |
| Q5T4L4 | 40S ribosomal protein S27 | RPS27 | 2 | 2 | 18,2 | 18,2 | 9 | + | + | + |  | + |  |
| P42785 | Lysosomal Pro-X carboxypeptidase | PRCP | 1 | 1 | 2,4 | 2,4 | 2 | + |  | + |  | + | + |
| P46940 | Ras GTPase-activating-like protein IQGAP1 | IQGAP1 | 10 | 10 | 7,2 | 7,2 | 68 | + | + | + |  | + | + |
| P47756-2 | F-actin-capping protein subunit beta | CAPZB | 4 | 4 | 18 | 18 | 19 | + | + | + |  | + |  |
| P48595 | Serpin B10 | SERPINB10 | 11 | 11 | 37,8 | 37,8 | 40 | + | + | + |  |  |  |
| P48960-2 | CD97 antigen | CD97 | 3 | 3 | 6,3 | 6,3 | 3 | + |  | + |  |  |  |
| P49247 | Ribose-5-phosphate isomerase | RPIA | 3 | 3 | 11,9 | 11,9 | 10 | + | + |  | + | + |  |
| P50395 | Rab GDP dissociation inhibitor beta | GDI2 | 17 | 11 | 44,9 | 29 | 59 | + | + | + |  | + | + |
| P50552 | Vasodilator-stimulated phosphoprotein | VASP | 11 | 11 | 20,3 | 20,3 | 46 | + | + | + |  | + | + |
| P50570-3 | Dynamin-2 | DNM2 | 3 | 3 | 3,6 | 3,6 | 3 |  |  | + |  |  |  |
| P50995-2 | Annexin A11 | ANXA11 | 16 | 16 | 38,3 | 38,3 | 48 | + | + | + | + | + | + |
| P51858-2 | Hepatoma-derived growth factor | HDGF | 3 | 3 | 11,6 | 11,6 | 9 | + | + |  |  | + | + |
| P52209-2 | 6-phosphogluconate dehydrogenase, decarboxylating | PGD | 10 | 10 | 31,9 | 31,9 | 30 | + | + | + |  | + | + |
| P52565 | Rho GDP-dissociation inhibitor 1 | ARHGDIA | 5 | 5 | 30,4 | 30,4 | 37 | + | + | + |  |  | + |
| P52566 | Rho GDP-dissociation inhibitor 2 | ARHGDIB | 16 | 16 | 62,7 | 62,7 | 175 | + | + | + |  | + | + |
| P52735-3 | Guanine nucleotide exchange factor VAV2 | VAV2 | 1 | 1 | 3,2 | 3,2 | 0 |  |  | + |  | + |  |
| P52907 | F-actin-capping protein subunit alpha-1 | CAPZA1 | 7 | 7 | 32,2 | 32,2 | 22 | + | + |  | + | + |  |
| P53618 | Coatomer subunit beta | COPB1 | 1 | 1 | 2 | 2 | 1 |  |  | + |  | + | + |
| P53634 | Dipeptidyl peptidase 1 | CTSC | 3 | 3 | 7,6 | 7,6 | 14 | + | + | + |  | + | + |
| P53999 | Activated RNA polymerase II transcriptional coactivator p15 | SUB1 | 1 | 1 | 18,9 | 18,9 | 15 | + | + | + |  | + |  |
| P55072 | Transitional endoplasmic reticulum ATPase | VCP | 3 | 3 | 4,6 | 4,6 | 3 | + | + |  | + | + | + |
| P58546 | Myotrophin | MTPN | 3 | 3 | 44,1 | 44,1 | 8 | + |  |  |  |  |  |
| P59666 | Neutrophil defensin 3 | DEFA3 | 6 | 6 | 34 | 34 | 208 | + | + | + | + | + | + |
| P60174-1 | Triosephosphate isomerase | TPI1 | 27 | 27 | 92 | 92 | 329 | + | + | + | + | + | + |
| P60709 | Actin, cytoplasmic 1 | ACTB | 29 | 0 | 61,6 | 0 | 488 | + | + | + | + | + | + |
| P61224-2 | Ras-related protein Rap-1b | RAP1B | 3 | 3 | 24,8 | 24,8 | 8 | + |  | + |  |  |  |
| P61604 | 10 kDa heat shock protein, mitochondrial | HSPE1 | 7 | 7 | 67,6 | 67,6 | 43 | + | + | + | + | + | + |
| P61626 | Lysozyme C | LYZ | 11 | 11 | 63,5 | 63,5 | 665 | + | + | + |  | + | + |
| P61769 | Beta-2-microglobulin | B2M | 3 | 3 | 35,3 | 35,3 | 28 | + | + | + | + | + | + |
| P61970 | Nuclear transport factor 2 | NUTF2 | 2 | 2 | 17,3 | 17,3 | 7 | + |  |  |  | + |  |
| P62258 | 14-3-3 protein epsilon | YWHAE | 4 | 4 | 25,9 | 25,9 | 7 | + |  |  |  | + |  |
| P62263 | 40S ribosomal protein S14 | RPS14 | 2 | 2 | 9,3 | 9,3 | 52 | + | + | + |  | + |  |
| P62318-2 | Small nuclear ribonucleoprotein Sm D3 | SNRPD3 | 1 | 1 | 8,3 | 8,3 | 4 | + |  | + | + | + |  |
| P62328 | Thymosin beta-4 | TMSB4X | 8 | 8 | 88,6 | 88,6 | 144 | + | + | + |  | + | + |
| Q5T8U3 | 60S ribosomal protein L7a | RPL7A | 3 | 3 | 5,8 | 5,8 | 10 | + | + |  |  |  |  |
| P62805 | Histone H4 | HIST1H4A | 8 | 8 | 55,3 | 55,3 | 112 | + | + | + |  |  |  |
| P62873-2 | Guanine nucleotide-binding protein G(I)/G(S)/G(T) subunit beta-1 | GNB1 | 8 | 5 | 30,4 | 20,8 | 19 | + |  | + |  | + | + |
| P62937 | Peptidyl-prolyl cis-trans isomerase A | PPIA | 14 | 14 | 86,1 | 86,1 | 336 | + | + | + | + | + | + |
| P62942 | Peptidyl-prolyl cis-trans isomerase FKBP1A | FKBP1A | 5 | 5 | 64,8 | 64,8 | 33 | + | + |  | + | + |  |
| P63261 | Actin, cytoplasmic 2 | ACTG1 | 29 | 1 | 61,6 | 4,5 | 31 | + | + | + |  |  | + |
| P68133 | Actin, alpha skeletal muscle | ACTA1 | 18 | 1 | 33,2 | 4,2 | 4 | + | + | + |  | + |  |
| P68871 | Hemoglobin subunit beta | HBB | 29 | 10 | 100 | 55,1 | 4416 | + | + | + |  | + | + |
| P69892 | Hemoglobin subunit gamma-2 | HBG2 | 13 | 11 | 76,2 | 69,4 | 47 | + | + | + |  | + |  |
| P69905 | Hemoglobin subunit alpha | HBA1 | 22 | 8 | 98,6 | 28,9 | 3235 | + | + | + | + | + | + |
| Q5TFQ8 | Signal-regulatory protein beta-1 isoform 3 | SIRPB1 | 4 | 4 | 13,3 | 13,3 | 6 | + | + | + |  | + |  |
| P78417 | Glutathione S-transferase omega-1 | GSTO1 | 5 | 5 | 19,9 | 19,9 | 5 | + | + |  | + | + | + |
| P78536-2 | Disintegrin and metalloproteinase domain-containing protein 17 | ADAM17 | 1 | 1 | 1,7 | 1,7 | 1 |  | + |  | + | + | + |
| X6R8F3 | Neutrophil gelatinase-associated lipocalin | LCN2 | 13 | 13 | 64 | 64 | 298 | + | + | + | + | + | + |
| P80511 | Protein S100-A12 | S100A12 | 15 | 15 | 64,1 | 64,1 | 231 | + | + | + | + | + | + |
| P80723 | Brain acid soluble protein 1 | BASP1 | 11 | 11 | 75,3 | 75,3 | 132 | + | + | + |  | + | + |
| P81605 | Dermcidin | DCD | 5 | 5 | 46,4 | 46,4 | 33 | + | + | + |  | + | + |
| P98160 | Basement membrane-specific heparan sulfate proteoglycan core protein | HSPG2 | 5 | 5 | 1,6 | 1,6 | 64 | + | + | + |  | + | + |
| Q00688 | Peptidyl-prolyl cis-trans isomerase FKBP3 | FKBP3 | 1 | 1 | 6,2 | 6,2 | 2 | + |  |  |  |  |  |
| Q01082-3 | Spectrin beta chain, non-erythrocytic 1 | SPTBN1 | 4 | 3 | 2,8 | 2,4 | 4 | + | + | + |  | + |  |
| Q01518 | Adenylyl cyclase-associated protein 1 | CAP1 | 27 | 1 | 53,3 | 4,8 | 4 | + | + | + |  | + |  |
| Q01518-2 | Adenylyl cyclase-associated protein 1 | CAP1 | 27 | 1 | 53,2 | 4,6 | 140 | + | + | + |  | + |  |
| Q02413 | Desmoglein-1 | DSG1 | 2 | 2 | 3,1 | 3,1 | 2 | + |  |  | + | + | + |
| Q02750-2 | Dual specificity mitogen-activated protein kinase kinase 1 | MAP2K1 | 2 | 2 | 3,5 | 3,5 | 10 | + | + | + | + | + | + |
| Q03518 | Antigen peptide transporter 1 | TAP1 | 1 | 1 | 1,4 | 1,4 | 5 |  | + | + | + | + | + |
| Q04721 | Neurogenic locus notch homolog protein 2 | NOTCH2 | 1 | 1 | 0,6 | 0,6 | 1 |  |  | + |  | + | + |
| Q04760-2 | Lactoylglutathione lyase | GLO1 | 2 | 2 | 15,4 | 15,4 | 2 | + |  |  |  | + | + |
| Q05315 | Galectin-10 | CLC | 4 | 4 | 33,8 | 33,8 | 37 | + | + | + |  | + | + |
| Q5VSF9 | Glutamate receptor ionotropic, NMDA 1 | GRIN1 | 1 | 1 | 1,5 | 1,5 | 2 |  |  | + |  | + | + |
| Q05BV3-2 | Echinoderm microtubule-associated protein-like 5 | EML5 | 1 | 1 | 2,1 | 2,1 | 1 |  |  | + |  |  |  |
| Q08211 | ATP-dependent RNA helicase A | DHX9 | 1 | 1 | 1,3 | 1,3 | 1 |  |  | + | + | + |  |
| Q08289-10 | Voltage-dependent L-type calcium channel subunit beta-2 | CACNB2 | 2 | 2 | 5,5 | 5,5 | 1 |  |  | + |  | + |  |
| Q08380 | Galectin-3-binding protein | LGALS3BP | 1 | 1 | 2,7 | 2,7 | 1 | + |  |  | + | + | + |
| Q0VD83-3 | Apolipoprotein B receptor | APOBR | 1 | 1 | 1,6 | 1,6 | 2 |  | + |  |  |  |  |
| Q12789-3 | General transcription factor 3C polypeptide 1 | GTF3C1 | 1 | 1 | 0,8 | 0,8 | 1 | + |  |  |  |  |  |
| Q12906-5 | Interleukin enhancer-binding factor 3 | ILF3 | 3 | 3 | 3,5 | 3,5 | 11 | + | + |  | + | + | + |
| Q13029-5 | PR domain zinc finger protein 2 | PRDM2 | 1 | 1 | 0,7 | 0,7 | 0 |  |  | + |  | + |  |
| Q13177 | Serine/threonine-protein kinase PAK 2 | PAK2 | 1 | 1 | 3,1 | 3,1 | 3 | + | + |  |  |  | + |
| Q13185 | Chromobox protein homolog 3 | CBX3 | 4 | 4 | 21,3 | 21,3 | 22 | + | + | + |  | + |  |
| Q13231-3 | Chitotriosidase-1 | CHIT1 | 12 | 12 | 43 | 43 | 37 | + | + | + |  | + | + |
| Q13474-2 | Dystrophin-related protein 2 | DRP2 | 1 | 1 | 1,4 | 1,4 | 3 |  |  | + |  |  |  |
| Q14019 | Coactosin-like protein | COTL1 | 11 | 11 | 60,6 | 60,6 | 47 | + | + | + | + | + |  |
| Q14152-2 | Eukaryotic translation initiation factor 3 subunit A | EIF3A | 1 | 1 | 0,6 | 0,6 | 2 | + | + |  |  | + |  |
| Q14161-3 |  |  | 1 | 1 | 2,6 | 2,6 | 1 |  |  | + |  |  |  |
| Q14202-2 |  |  | 1 | 1 | 1,5 | 1,5 | 1 |  |  | + |  |  |  |
| Q14651 | Plastin-1 | PLS1 | 3 | 1 | 4,9 | 1,6 | 1 | + | + | + |  |  |  |
| Q14694 | Ubiquitin carboxyl-terminal hydrolase 10 | USP10 | 1 | 1 | 1,8 | 1,8 | 1 |  |  | + |  |  |  |
| Q14789-4 | Golgin subfamily B member 1 | GOLGB1 | 1 | 1 | 0,3 | 0,3 | 0 | + |  |  |  |  |  |
| Q15084-3 | Protein disulfide-isomerase A6 | PDIA6 | 3 | 3 | 10,5 | 10,5 | 4 | + |  |  |  | + |  |
| Q15365 | Poly(rC)-binding protein 1 | PCBP1 | 4 | 2 | 13,2 | 7,6 | 67 | + | + | + |  | + | + |
| Q15555-4 | Microtubule-associated protein RP/EB family member 2 | MAPRE2 | 1 | 1 | 7,3 | 7,3 | 2 | + |  |  |  |  |  |
| Q15573-2 | TATA box-binding protein-associated factor RNA polymerase I subunit A | TAF1A | 1 | 1 | 3,3 | 3,3 | 1 |  |  | + |  | + |  |
| Q155Q3-5 | Dixin | DIXDC1 | 1 | 1 | 8,7 | 8,7 | 1 |  |  | + |  |  |  |
| Q15746-4 | Myosin light chain kinase, smooth muscle | MYLK | 3 | 3 | 2,5 | 2,5 | 3 | + | + | + |  | + | + |
| Q15928 | Zinc finger protein 141 | ZNF141 | 1 | 1 | 2,3 | 2,3 | 14 | + | + | + |  |  |  |
| Q1X8D7-3 |  |  | 1 | 1 | 3 | 3 | 1 |  |  | + |  |  |  |
| Q2KJ03 | Putative uncharacterized protein ZNRD1-AS1 | ZNRD1-AS1 | 1 | 1 | 7 | 7 | 2 |  |  | + |  |  |  |
| Q3KQU3-2 | MAP7 domain-containing protein 1 | MAP7D1 | 2 | 2 | 4,5 | 4,5 | 2 |  |  | + |  |  |  |
| Q494V2 | Coiled-coil domain-containing protein 37 | CCDC37 | 1 | 1 | 2,6 | 2,6 | 1 |  |  | + |  |  |  |
| Q4G0N8-2 | Sodium/hydrogen exchanger 10 | SLC9C1 | 2 | 2 | 2,5 | 2,5 | 3 |  |  | + |  |  |  |
| Q4VC31 | Coiled-coil domain-containing protein 58 | CCDC58 | 6 | 6 | 35,4 | 35,4 | 23 | + | + | + |  |  |  |
| Q4VX76-2 | Synaptotagmin-like protein 3 | SYTL3 | 1 | 1 | 2,8 | 2,8 | 5 | + |  |  |  |  |  |
| Q4VXW1 |  | COL9A3 | 1 | 1 | 13,6 | 13,6 | 1 |  |  | + |  |  | + |
| Q562R1 | Beta-actin-like protein 2 | ACTBL2 | 9 | 1 | 18,1 | 2,7 | 1 | + | + | + | + | + |  |
| Q58DX5 | Inactive N-acetylated-alpha-linked acidic dipeptidase-like protein 2 | NAALADL2 | 1 | 1 | 2,6 | 2,6 | 2 |  |  | + | + | + |  |
| Q58FF8 | Putative heat shock protein HSP 90-beta 2 | HSP90AB2P | 3 | 1 | 10,8 | 6,3 | 1 | + | + | + |  |  |  |
| Q59FP8 | Neogenin | NEO1 | 4 | 4 | 4,8 | 4,8 | 8 | + | + | + |  |  | + |
| U3KQA9 | Protein phosphatase 1J | PPM1J | 1 | 1 | 5,6 | 5,6 | 1 |  |  | + |  |  |  |
| Q5JVD6 |  | CNTRL | 1 | 1 | 2,9 | 2,9 | 1 | + |  |  |  | + |  |
| Q5SZC9 | Costars family protein ABRACL | ABRACL | 1 | 1 | 23,9 | 23,9 | 4 | + | + |  |  |  |  |
| Q5T655 | Cilia- and flagella-associated protein 58 | CFAP58 | 1 | 1 | 1 | 1 | 1 |  |  | + |  |  |  |
| Q5TB53 |  | TM9SF3 | 1 | 1 | 5,9 | 5,9 | 1 |  |  | + |  |  |  |
| Q5TEC6 | Histone H3 | HIST2H3PS2 | 2 | 1 | 13,2 | 8,1 | 5 | + | + | + |  |  |  |
| Q5VSY0-2 | G kinase-anchoring protein 1 | GKAP1 | 1 | 1 | 4,4 | 4,4 | 2 | + |  |  |  |  |  |
| Q5VU65-2 | Nuclear pore membrane glycoprotein 210-like | NUP210L | 2 | 2 | 2,1 | 2,1 | 1 |  |  | + |  |  |  |
| Q5VUJ6-2 | Leucine-rich repeat and calponin homology domain-containing protein 2 | LRCH2 | 1 | 1 | 3,5 | 3,5 | 1 |  |  | + |  |  |  |
| Q5VXJ0 | Lipase member K | LIPK | 1 | 1 | 3,5 | 3,5 | 1 |  |  | + |  |  |  |
| Q5VZR0 | Golgi-associated plant pathogenesis-related protein 1 | GLIPR2 | 3 | 3 | 37,5 | 37,5 | 17 | + | + | + |  | + |  |
| Q60FE5 |  | FLNA | 95 | 1 | 46 | 0,6 | 1 | + | + | + |  | + | + |
| Q63ZY6-2 | Putative methyltransferase NSUN5C | NSUN5P2 | 1 | 1 | 11,1 | 11,1 | 1 |  |  | + |  |  |  |
| Q6P4A8 | Phospholipase B-like 1 | PLBD1 | 5 | 5 | 15,9 | 15,9 | 20 | + | + | + |  | + |  |
| Q6UUV7-3 | CREB-regulated transcription coactivator 3 | CRTC3 | 1 | 1 | 1,8 | 1,8 | 1 |  |  | + |  | + |  |
| Q6UX06 | Olfactomedin-4 | OLFM4 | 10 | 10 | 25,3 | 25,3 | 45 | + | + | + |  | + | + |
| Q6WKZ4-3 | Rab11 family-interacting protein 1 | RAB11FIP1 | 2 | 2 | 3,1 | 3,1 | 3 | + | + |  |  | + |  |
| Q6ZNB6 | NF-X1-type zinc finger protein NFXL1 | NFXL1 | 1 | 1 | 1,5 | 1,5 | 4 | + | + | + |  |  |  |
| Q6ZT07 | TBC1 domain family member 9 | TBC1D9 | 1 | 1 | 1,9 | 1,9 | 1 |  |  | + |  | + |  |
| Q702N8-2 | Xin actin-binding repeat-containing protein 1 | XIRP1 | 2 | 2 | 2,4 | 2,4 | 9 | + | + | + |  | + |  |
| Q709C8-4 | Vacuolar protein sorting-associated protein 13C | VPS13C | 2 | 2 | 0,5 | 0,5 | 0 | + |  | + |  | + |  |
| Q7Z2W7-4 |  |  | 1 | 1 | 1,5 | 1,5 | 8 | + | + |  |  |  |  |
| Q7Z406 | Myosin-14 | MYH14 | 3 | 1 | 1,6 | 0,5 | 1 | + | + | + |  | + |  |
| Q7Z4L5-2 | Tetratricopeptide repeat protein 21B | TTC21B | 1 | 1 | 2,7 | 2,7 | 1 |  |  | + |  | + |  |
| Q7Z699 | Sprouty-related, EVH1 domain-containing protein 1 | SPRED1 | 2 | 2 | 5,2 | 5,2 | 3 | + | + | + |  | + | + |
| Q86UP2-2 | Kinectin | KTN1 | 2 | 2 | 1,9 | 1,9 | 2 | + |  |  | + | + |  |
| Q86VF2-5 |  |  | 1 | 1 | 2,5 | 2,5 | 1 |  |  | + |  |  |  |
| Q86Y37-4 | CDK2-associated and cullin domain-containing protein 1 | CACUL1 | 1 | 1 | 12,2 | 12,2 | 1 |  |  | + |  |  |  |
| Q8IUC1 | Keratin-associated protein 11-1 | KRTAP11-1 | 1 | 1 | 6,7 | 6,7 | 12 | + | + | + |  |  |  |
| Q8IVF2-3 | Protein AHNAK2 | AHNAK2 | 2 | 2 | 0,5 | 0,5 | 3 | + |  | + |  | + |  |
| Q8IWA0 | WD repeat-containing protein 75 | WDR75 | 1 | 1 | 3 | 3 | 1 |  |  | + |  |  |  |
| Q8IXQ6 | Poly [ADP-ribose] polymerase 9 | PARP9 | 1 | 1 | 1,2 | 1,2 | 1 | + | + |  |  | + | + |
| X6RHX1 |  | SCYL3 | 1 | 1 | 4,4 | 4,4 | 1 |  |  | + |  |  |  |
| Q8N157 | Jouberin | AHI1 | 1 | 1 | 1,1 | 1,1 | 1 |  |  | + |  | + |  |
| Q8N4W6 | DnaJ homolog subfamily C member 22 | DNAJC22 | 1 | 1 | 7,9 | 7,9 | 1 |  |  | + |  | + |  |
| Q8N7X1 | RNA-binding motif protein, X-linked-like-3 | RBMXL3 | 1 | 1 | 1,2 | 1,2 | 1 |  |  | + |  |  |  |
| Q8NC51-4 | Plasminogen activator inhibitor 1 RNA-binding protein | SERBP1 | 2 | 2 | 6,2 | 6,2 | 17 | + | + | + |  | + |  |
| Q8NCM8 | Cytoplasmic dynein 2 heavy chain 1 | DYNC2H1 | 2 | 2 | 0,7 | 0,7 | 5 |  |  | + |  |  |  |
| Q8NEC5 | Cation channel sperm-associated protein 1 | CATSPER1 | 1 | 1 | 2,1 | 2,1 | 1 |  |  | + |  |  |  |
| Q8NHQ8-2 | Ras association domain-containing protein 8 | RASSF8 | 2 | 2 | 8,7 | 8,7 | 2 |  |  | + |  | + |  |
| Q8TBY9 | WD repeat-containing protein 66 | WDR66 | 1 | 1 | 1 | 1 | 0 |  |  | + |  |  |  |
| Q8TD19 | Serine/threonine-protein kinase Nek9 | NEK9 | 1 | 1 | 1,4 | 1,4 | 4 |  |  | + |  |  |  |
| Q8TDN1 | Potassium voltage-gated channel subfamily G member 4 | KCNG4 | 1 | 1 | 1,7 | 1,7 | 2 |  |  | + |  | + |  |
| Q8TE73 | Dynein heavy chain 5, axonemal | DNAH5 | 1 | 1 | 0,2 | 0,2 | 185 | + | + | + |  | + | + |
| Q8WWL2-4 | Protein spire homolog 2 | SPIRE2 | 1 | 1 | 1,9 | 1,9 | 1 |  |  | + |  |  |  |
| Q8WXR4-4 | Myosin-IIIb | MYO3B | 1 | 1 | 1,8 | 1,8 | 2 |  |  | + |  |  |  |
| Q92820 | Gamma-glutamyl hydrolase | GGH | 2 | 2 | 9,1 | 9,1 | 4 | + |  | + |  | + | + |
| Q92904-2 |  |  | 1 | 1 | 7,6 | 7,6 | 1 |  |  | + |  |  |  |
| Q96A65-2 | Exocyst complex component 4 | EXOC4 | 1 | 1 | 5,3 | 5,3 | 0 |  |  | + |  | + |  |
| Q96AE7-2 | Tetratricopeptide repeat protein 17 | TTC17 | 1 | 1 | 1 | 1 | 1 |  |  | + |  |  |  |
| Q96B60 |  | NT5E | 1 | 1 | 10,6 | 10,6 | 1 |  |  | + |  | + |  |
| Q96C19 | EF-hand domain-containing protein D2 | EFHD2 | 7 | 7 | 33,8 | 33,8 | 25 | + | + |  |  |  | + |
| Q96EK6 | Glucosamine 6-phosphate N-acetyltransferase | GNPNAT1 | 12 | 12 | 59,2 | 59,2 | 117 | + | + | + |  |  |  |
| Q96I59-2 | Probable asparagine--tRNA ligase, mitochondrial | NARS2 | 1 | 1 | 8,8 | 8,8 | 0 |  |  | + |  |  |  |
| Q96JB2-2 | Conserved oligomeric Golgi complex subunit 3 | COG3 | 1 | 1 | 3,4 | 3,4 | 1 |  |  | + |  |  |  |
| Q96JM2 | Zinc finger protein 462 | ZNF462 | 1 | 1 | 0,5 | 0,5 | 3 |  | + | + |  | + |  |
| Q96JM4 | Leucine-rich repeat and IQ domain-containing protein 1 | LRRIQ1 | 2 | 1 | 1,7 | 1 | 1 |  |  | + |  |  |  |
| Q96M20-3 | Cyclic nucleotide-binding domain-containing protein 2 | CNBD2 | 1 | 1 | 4 | 4 | 4 |  |  | + |  |  |  |
| Q96M86 | Dynein heavy chain domain-containing protein 1 | DNHD1 | 2 | 2 | 0,5 | 0,5 | 1 |  |  | + |  |  |  |
| Q96NL6 | Sodium channel and clathrin linker 1 | SCLT1 | 1 | 1 | 1,7 | 1,7 | 0 | + |  |  |  | + |  |
| Q96Q40-2 | Cyclin-dependent kinase 15 | CDK15 | 1 | 1 | 4,5 | 4,5 | 1 |  |  | + |  |  |  |
| Q96Q89-4 | Kinesin-like protein KIF20B | KIF20B | 1 | 1 | 0,5 | 0,5 | 55 | + | + | + |  |  |  |
| Q96QZ7-6 | Membrane-associated guanylate kinase, WW and PDZ domain-containing protein 1 | MAGI1 | 3 | 3 | 2,3 | 2,3 | 12 | + | + | + |  | + |  |
| Q96RL7-4 | Vacuolar protein sorting-associated protein 13A | VPS13A | 1 | 1 | 0,5 | 0,5 | 1 |  |  | + |  | + |  |
| Q99497 | Protein deglycase DJ-1 | PARK7 | 7 | 7 | 42,9 | 42,9 | 30 | + | + | + |  | + | + |
| Q99747-2 | Gamma-soluble NSF attachment protein | NAPG | 1 | 1 | 6,5 | 6,5 | 1 |  |  | + |  |  |  |
| Q9BQB4 | Sclerostin | SOST | 1 | 1 | 5,2 | 5,2 | 2 |  |  | + | + | + | + |
| Q9BRA2 | Thioredoxin domain-containing protein 17 | TXNDC17 | 3 | 3 | 30,1 | 30,1 | 25 | + | + | + |  |  |  |
| Q9BRP8-2 | Partner of Y14 and mago | WIBG | 2 | 2 | 15,3 | 15,3 | 2 | + |  |  |  |  |  |
| Q9BS26 | Endoplasmic reticulum resident protein 44 | ERP44 | 2 | 2 | 7,9 | 7,9 | 4 | + |  |  |  | + | + |
| Q9BWG6-3 |  |  | 1 | 1 | 4,9 | 4,9 | 1 |  | + |  |  |  |  |
| Q9BWJ5 | Splicing factor 3B subunit 5 | SF3B5 | 1 | 1 | 17,4 | 17,4 | 1 | + |  |  |  | + |  |
| Q9BXR6 | Complement factor H-related protein 5 | CFHR5 | 1 | 1 | 1,8 | 1,8 | 1 |  | + |  | + | + |  |
| Q9C0D5-2 | Protein TANC1 | TANC1 | 1 | 1 | 1,7 | 1,7 | 1 |  |  | + |  | + |  |
| Q9C0G6 | Dynein heavy chain 6, axonemal | DNAH6 | 2 | 2 | 0,9 | 0,9 | 2 |  |  | + |  |  |  |
| Q9H0Q0 | Protein FAM49A | FAM49A | 2 | 2 | 6,8 | 6,8 | 2 | + |  | + |  | + |  |
| X6R772 | Glutamyl-tRNA(Gln) amidotransferase subunit A, mitochondrial | QRSL1 | 1 | 1 | 3,4 | 3,4 | 5 | + | + | + |  |  |  |
| Q9H156 | SLIT and NTRK-like protein 2 | SLITRK2 | 2 | 2 | 2,2 | 2,2 | 7 | + | + | + |  |  |  |
| Q9H1E3 | Nuclear ubiquitous casein and cyclin-dependent kinase substrate 1 | NUCKS1 | 1 | 1 | 7 | 7 | 2 | + | + |  |  | + |  |
| Q9H347 | Ubiquilin-3 | UBQLN3 | 1 | 1 | 2,1 | 2,1 | 2 |  |  | + |  |  |  |
| Q9H477-2 | Ribokinase | RBKS | 2 | 2 | 9,8 | 9,8 | 3 | + | + | + |  |  |  |
| Q9H4F8 | SPARC-related modular calcium-binding protein 1 | SMOC1 | 1 | 1 | 2,3 | 2,3 | 18 | + | + | + |  |  | + |
| Q9H8K7 | Uncharacterized protein C10orf88 | C10orf88 | 1 | 1 | 6,5 | 6,5 | 1 |  |  | + |  |  |  |
| Q9HB71-3 | Calcyclin-binding protein | CACYBP | 1 | 1 | 7,6 | 7,6 | 2 | + | + |  |  |  |  |
| Q9HBL7 | Plasminogen receptor (KT) | PLGRKT | 2 | 2 | 10,9 | 10,9 | 1 |  |  | + |  |  | + |
| Q9HD89 | Resistin | RETN | 6 | 6 | 59,3 | 59,3 | 137 | + | + | + | + | + | + |
| Q9NRX4-2 | 14 kDa phosphohistidine phosphatase | PHPT1 | 1 | 1 | 9,7 | 9,7 | 2 | + | + |  |  |  |  |
| Q9NVE4 | Coiled-coil domain-containing protein 87 | CCDC87 | 1 | 1 | 1,8 | 1,8 | 1 | + |  |  |  |  |  |
| Q9NZ71-9 |  |  | 1 | 1 | 1 | 1 | 1 |  |  | + |  |  |  |
| Q9NZD4 | Alpha-hemoglobin-stabilizing protein | AHSP | 3 | 3 | 32,4 | 32,4 | 11 | + | + |  |  | + | + |
| Q9NZT1 | Calmodulin-like protein 5 | CALML5 | 5 | 5 | 31,5 | 31,5 | 16 | + | + | + |  |  |  |
| Q9P2F6-4 | Rho GTPase-activating protein 20 | ARHGAP20 | 1 | 1 | 1,3 | 1,3 | 1 |  | + |  |  |  |  |
| U3KQ56 | Glyoxylate reductase/hydroxypyruvate reductase | GRHPR | 2 | 2 | 12,8 | 12,8 | 5 | + |  | + |  | + |  |
| Q9UJ37 | Alpha-N-acetylgalactosaminide alpha-2,6-sialyltransferase 2 | ST6GALNAC2 | 1 | 1 | 3,5 | 3,5 | 2 |  |  | + |  | + |  |
| Q9ULU8 | Calcium-dependent secretion activator 1 | CADPS | 2 | 2 | 1,8 | 1,8 | 2 |  | + | + |  | + |  |
| Q9ULX5 | RING finger protein 112 | RNF112 | 1 | 1 | 3,6 | 3,6 | 1 |  |  | + | + | + | + |
| Q9ULZ3-3 | Apoptosis-associated speck-like protein containing a CARD | PYCARD | 3 | 3 | 23 | 23 | 17 | + | + | + | + | + | + |
| Q9UM21 | Alpha-1,3-mannosyl-glycoprotein 4-beta-N-acetylglucosaminyltransferase A | MGAT4A | 1 | 1 | 2,8 | 2,8 | 1 |  |  | + |  | + |  |
| Q9UPV7 | Protein KIAA1045 | KIAA1045 | 1 | 1 | 3,8 | 3,8 | 1 |  | + |  |  |  |  |
| Q9Y2G4-4 | Ankyrin repeat domain-containing protein 6 | ANKRD6 | 1 | 1 | 2 | 2 | 1 |  |  | + |  |  |  |
| Q9Y2X9-2 | Zinc finger protein 281 | ZNF281 | 1 | 1 | 2,9 | 2,9 | 1 |  |  | + |  | + |  |
| Q9Y3B4 | Splicing factor 3B subunit 6 | SF3B6 | 1 | 1 | 6,4 | 6,4 | 10 | + | + | + |  | + |  |
| Q9Y3T9 | Nucleolar complex protein 2 homolog | NOC2L | 1 | 1 | 2,4 | 2,4 | 1 |  |  | + |  | + | + |
| Q9Y442-2 | Uncharacterized protein C22orf24 | C22orf24 | 1 | 1 | 41,9 | 41,9 | 1 |  |  | + |  |  |  |
| Q9Y490 | Talin-1 | TLN1 | 16 | 16 | 9,1 | 9,1 | 43 | + | + | + |  | + | + |
| X6RDF7 |  | SLC22A7 | 1 | 1 | 6,6 | 6,6 | 1 |  |  | + |  |  | + |
| Q9Y6V0-2 | Protein piccolo | PCLO | 2 | 2 | 0,4 | 0,4 | 5 | + | + | + |  |  |  |
|  | Total Proteins |  |  |  |  |  |  | 530 | 451 | 640 | 183 | 506 | 337 |

**Table S-2.** List of significant proteins identified by mass spectrometry in NETs samples.

| **Protein IDs** | **Protein name** | **Gene name** | **Fold Change SLE/LN vs CTR** | **-Log_10_ P-value SLE/LN vs CTR** | **Fold Change LN vs SLE** | **-Log_10_ P-value LN vs SLE** |
| --- | --- | --- | --- | --- | --- | --- |
| A0A0G2JNW2 | Protein-tyrosine-phosphatase | PTPRC | -1.831 | 1.72 | 0.205 |  |
| A0A087WV23 | SH3 domain-binding glutamic acid-rich-like protein 3 | SH3BGRL3 | 6.529 | 8.737 | -0.007 |  |
| A0A087WV66 | Antigen KI-67 | MKI67 | -5.094 | 5.533 | -1.192 |  |
| A0A087WVE2 | Ficolin-1 | FCN1 | -2.117 | 2.762 | -0.137 |  |
| A0A087WWK2 | Alpha-N-acetylgalactosaminide alpha-2,6-sialyltransferase 3 | ST6GALNAC3 | 3.459 | 1.855 | 2.032 |  |
| A0A087WX70 | Protocadherin-15 | PCDH15 | -4.336 | 5.412 | -0.996 |  |
| A0A087WXP0 | Azurocidin | AZU1 | -6.845 | 16.519 | -0.918 |  |
| H0YFY6 | Nuclear mitotic apparatus protein 1 | NUMA1 | -2.832 | 2.291 | 3.472 |  |
| A0A087WZH7 | Myristoylated alanine-rich C-kinase substrate | MARCKS | 4.218 | 7.651 | -0.092 |  |
| A0A087WZR4 | Low affinity immunoglobulin gamma Fc region receptor III-B | FCGR3B | -2.575 | 3.438 | 0.804 |  |
| A0A087X0L4 | Interferon-inducible protein AIM2 | AIM2 | 7.099 | 6.616 | -1.162 |  |
| A0A087X1S2 | Nuclease-sensitive element-binding protein 1 | YBX1 | 3.662 | 5.834 | -1.185 |  |
| A0A087X2C1 | Sorting nexin-20 | SNX20 | 2.05 | 2.447 | -0.254 |  |
| A0A096LPD5 | Serpin B11 | SERPINB11 | -4.64 | 7.487 | -1.139 |  |
| A0A0A0MR61 | Serine protease 57 | PRSS57 | -5.153 | 10.002 | 0.273 |  |
| H7C4R7 | Deoxyribonuclease | DNASE1L3 | 2.431 | 2.899 | -0.121 |  |
| C9J7N5 | Serpin I2 | SERPINI2 | -2.037 | 2.633 | 2.412 |  |
| Q5VZZ6 | CUGBP Elav-like family member 2 | CELF2 | 1.961 | 2.46 | -0.948 |  |
| A4FU16 | Rhomboid-related protein 3 | RHBDL3 | -6.401 | 14.275 | -0.546 |  |
| H7C571 | Transcription cofactor vestigial-like protein 3 | VGLL3 | 2.199 |  | -4.698 | 6.863 |
| Q5T7C4 | Putative high mobility group protein B1-like 1 | HMGB1 | 6.272 | 10.1 | 0.776 |  |
| E9PRD9 | Vascular non-inflammatory molecule 2 | VNN2 | -2.545 | 3.728 | 0.612 |  |
| J3QS39 | Ubiquitin-60S ribosomal protein L40 | UBB | 1.003 | 2.751 | -0.802 |  |
| H7C3M2 | 60S ribosomal protein L3 | RPL3 | -9.781 | 19.361 | -0.671 |  |
| F8W1R7 | Myosin light polypeptide 6 | MYL6 | 2.916 | 4.432 | 1.294 |  |
| C9J8P9 | Clathrin light chain A | CLTA | -4.947 | 13.368 | -1.333 |  |
| C9JIZ6 | Prosaposin | PSAP | 1.613 | 2.296 | 0.408 |  |
| C9JUY1 | AF4/FMR2 family member 3 | AFF3 | -4.296 | 4.794 | 1.752 |  |
| D6R9A6 | High mobility group protein B2 | HMGB2 | 1.977 | 2.206 | 0.72 |  |
| D6RG15 | Twinfilin-2 | TWF2 | 1.795 | 3.231 | -0.047 |  |
| E9PAQ1 | Properdin | CFP | -1.555 | 1.953 | -1.106 |  |
| E9PH88 | DNA mismatch repair protein PMS1 | PMS1 | -7.507 | 13.827 | -0.701 |  |
| E9PP60 | GDP-L-fucose synthase | TSTA3 | -4.078 | 8.317 | -0.333 |  |
| F5H6P7 | Protein mago nashi homolog | MAGOHB | -2.589 | 2.279 | -4.649 | 5.547 |
| F6TLX2 | Glyoxalase domain-containing protein 4 | GLOD4 | 1.386 |  | 1.496 | 3.599 |
| F8W6I7 | Heterogeneous nuclear ribonucleoprotein A1 | HNRNPA1 | 4.317 | 5.574 | 0.703 |  |
| H7C3D3 | Zyxin | ZYX | 5.833 | 10.174 | -1.768 |  |
| H0Y6Z7 | Receptor-type tyrosine-protein phosphatase F | PTPRF | 1.366 | 3.423 | -0.85 |  |
| H0YJT6 | Protein NRDE2 homolog | NRDE2 | 4.501 | 4.501 | -0.955 |  |
| H0YLY0 | Serine/threonine-protein kinase MRCK beta | CDC42BPB | -3.348 | 10.611 | -0.765 |  |
| H3BR01 | Kunitz-type protease inhibitor 1 | SPINT1 | 2.975 | 4.815 | -0.531 |  |
| H3BTN5 | Pyruvate kinase | PKM | 6.042 | 10.015 | -0.645 |  |
| H3BU51 | Neuroplastin | NPTN | 5.094 | 9.079 | -0.495 |  |
| H7BXD5 | Grancalcin | GCA | -1.281 | 1.969 | 1.205 |  |
| H7BZJ3 | Protein disulfide-isomerase A3 | PDIA3 | 3.811 | 7.488 | 1.07 |  |
| H7C3U4 | E3 ubiquitin-protein ligase MYCBP2 | MYCBP2 | 6.549 |  | 5.216 | 2.736 |
| J3KN67 | Tropomyosin alpha-3 chain | TPM3 | 2.579 | 3.547 | -0.221 |  |
| J3KPA1 | Cysteine-rich secretory protein 3 | CRISP3 | -3.479 | 4.901 | -0.003 |  |
| J3QSA3 | polyubiquitin-B | UBB | 2.504 | 2.285 | -0.549 |  |
| K7EJC1 | 26S proteasome non-ATPase regulatory subunit 8 | PSMD8 | -1.812 | 3.489 | -0.162 |  |
| M0R0S5 | Mast cell-expressed membrane protein 1 | MCEMP1 | -1.154 | 2.271 | 0.253 |  |
| O00299 | Chloride intracellular channel protein 1 | CLIC1 | 3.333 | 7.195 | -0.275 |  |
| O00560-2 | Syntenin-1 | SDCBP | -1.456 | 2.347 | 0.038 |  |
| O15400-2 | Syntaxin-7 | STX7 | 4.005 | 6.091 | 0.808 |  |
| O75083 | WD repeat-containing protein 1 | WDR1 | -1.464 |  | 3.179 | 2.897 |
| O75223 | Gamma-glutamylcyclotransferase | GGCT | 1.372 | 4.343 | -0.37 |  |
| O75368 | SH3 domain-binding glutamic acid-rich-like protein | SH3BGRL | 1.698 | 2.519 | -0.276 |  |
| O75531 | Barrier-to-autointegration factor | BANF1 | -1.149 | 2.098 | 0.757 |  |
| P00441 | Superoxide dismutase [Cu-Zn] | SOD1 | 6.041 | 6.485 | 0.938 |  |
| P01040 | Cystatin-A | CSTA | 4.633 | 7.456 | -0.504 |  |
| P02795 | Metallothionein-2 | MT2A | 1.759 | 2.859 | -0.665 |  |
| P04080 | Cystatin-B | CSTB | 5.489 | 12.435 | 0.261 |  |
| P04083 | Annexin A1 | ANXA1 | 1.402 | 5.546 | 1.2 | 3.938 |
| P04406 | Glyceraldehyde-3-phosphate dehydrogenase | GAPDH | -1.567 | 3.021 | -0.429 |  |
| P04839 | Cytochrome b-245 heavy chain | CYBB | 2.872 | 5.967 | 0.464 |  |
| P04899 | Guanine nucleotide-binding protein G(i) subunit alpha-2 | GNAI2 | -2.995 | 4.945 | 2.254 |  |
| P05109 | Protein S100-A8 | S100A8 | 1.065 | 2.11 | 0.669 |  |
| P05164-2 | Myeloperoxidase | MPO | -4.135 | 5.733 | 0.299 |  |
| P05204 | Non-histone chromosomal protein HMG-17 | HMGN2 | 1.865 | 2.831 | -0.474 |  |
| P06396-2 | Gelsolin | GSN | 3.564 | 7.288 | 0.586 |  |
| P06702 | Protein S100-A9 | S100A9 | 1.062 | 2.137 | 0.653 |  |
| P06733 | Alpha-enolase | ENO1 | 5.149 | 8.477 | 2.476 | 2.873 |
| P06733-2 | Alpha-enolase;MBP-1 | ENO1 | 5.074 | 9.022 | 2.829 | 8.035 |
| P07108 | Acyl-CoA-binding protein | DBI | 5.438 | 9.864 | 0.034 |  |
| P07237 | Protein disulfide-isomerase | P4HB | 2.954 | 3.349 | 0.713 |  |
| P07311 | Acylphosphatase-1 | ACYP1 | 1.359 | 1.865 | -1.337 |  |
| P07954-2 | Fumarate hydratase, mitochondrial | FH | -2.474 | 5.271 | -0.482 |  |
| P07996-2 | Thrombospondin-1 | THBS1 | -3.985 | 11.729 | -0.498 |  |
| P08246 | Neutrophil elastase | ELANE | -3.97 | 6.414 | 0.293 |  |
| P08311 | Cathepsin G | CTSG | -3.155 | 4.746 | 0.971 |  |
| P08670 | Vimentin | VIM | -1.15 | 2.119 | -0.738 |  |
| P10153 | Non-secretory ribonuclease | RNASE2 | 1.224 | 1.93 | 0.143 |  |
| P10412 | Histone H1.4 | HIST1H1E | 5.536 | 6.765 | -1.465 |  |
| P10768 | S-formylglutathione hydrolase | ESD | 2.824 |  | 2.307 | 2.775 |
| P11021 | 78 kDa glucose-regulated protein | HSPA5 | 1.199 | 2.266 | 0.724 |  |
| P11678 | Eosinophil peroxidase | EPX | -3.539 | 8.243 | -0.014 |  |
| P14174 | Macrophage migration inhibitory factor | MIF | 1.682 | 2.502 | 0.558 |  |
| P14543-2 | Nidogen-1 | NID1 | -3.486 | 4.603 | 0.288 |  |
| P15144 | Aminopeptidase N | ANPEP | -2.913 | 6.885 | -1.002 |  |
| P16949 | Stathmin | STMN1 | 3.786 | 5.509 | -0.595 |  |
| P18206-2 | Vinculin | VCL | 1.396 | 6.406 | -0.154 |  |
| P20160 | Azurocidin | AZU1 | -5.94 | 5.744 | -0.215 |  |
| P20700 | Lamin-B1 | LMNB1 | 2.723 | 2.044 | 0.086 |  |
| P23284 | Peptidyl-prolyl cis-trans isomerase B | PPIB | 4.027 | 5.493 | 0.639 |  |
| P26038 | Moesin | MSN | 1.837 | 2.126 | 1.108 |  |
| P26447 | Protein S100-A4 | S100A4 | 6.907 | 12.931 | -1.027 |  |
| P27105 | Erythrocyte band 7 integral membrane protein | STOM | -3.355 | 2.557 | 1.969 |  |
| P27695 | DNA-(apurinic or apyrimidinic site) lyase | APEX1 | 1.394 | 1.747 | 1.46 |  |
| P28066 | Proteasome subunit alpha type-5 | PSMA5 | -2.85 | 4.023 | 0.478 |  |
| P28799 | Granulins | GRN | 1.46 | 3.695 | -0.616 |  |
| P29350-2 | Tyrosine-protein phosphatase non-receptor type 6 | PTPN6 | 3.313 | 8.34 | 0.39 |  |
| P30086 | Phosphatidylethanolamine-binding protein 1 | PEBP1 | 5.316 | 9.426 | 1.384 |  |
| P30101 | Protein disulfide-isomerase A3 | PDIA3 | 4.805 | 7.936 | 0.557 |  |
| P31146 | Coronin-1A | CORO1A | 2.538 | 7.669 | -0.3 |  |
| P31949 | Protein S100-A11 | S100A11 | 2.695 | 10.226 | -0.21 |  |
| P32119 | Peroxiredoxin-2 | PRDX2 | 3.028 | 1.884 | 2.662 |  |
| P35555 | Fibrillin-1 | FBN1 | 6.003 | 7.932 | -0.969 |  |
| P35579 | Myosin-9 | MYH9 | 1.035 | 2.04 | -0.374 |  |
| P36222 | Chitinase-3-like protein 1 | CHI3L1 | -1.311 | 1.847 | -0.106 |  |
| P37802 | Transgelin-2 | TAGLN2 | 1.728 | 4.616 | 0.219 |  |
| P37837 | Transaldolase | TALDO1 | 1.064 | 3.761 | 0.496 |  |
| P41218 | Myeloid cell nuclear differentiation antigen | MNDA | -5.108 | 7.636 | 2.294 |  |
| P49247 | Ribose-5-phosphate isomerase | RPIA | 3.802 | 9.714 | 0.163 |  |
| P50552 | Vasodilator-stimulated phosphoprotein | VASP | 2.887 | 3.713 | -1.76 |  |
| P52565 | Rho GDP-dissociation inhibitor 1 | ARHGDIA | 1.059 | 3.293 | 0.361 |  |
| P52907 | F-actin-capping protein subunit alpha-1 | CAPZA1 | 4.526 | 7.789 | 1.018 |  |
| P60709 | Actin, cytoplasmic 1 | ACTB | 2.775 | 10.957 | 0.195 |  |
| P61970 | Nuclear transport factor 2 | NUTF2 | 2.661 |  | 3.303 | 3.631 |
| P62328 | Thymosin beta-4 | TMSB4X | 2.685 | 11.569 | -0.117 |  |
| P62805 | Histone H4 | HIST1H4A | -2.52 | 1.762 | 0.548 |  |
| P62873-2 | Guanine nucleotide-binding protein G(I)/G(S)/G(T) subunit beta-1 | GNB1 | -2.449 | 3.347 | 0.726 |  |
| P62937 | Peptidyl-prolyl cis-trans isomerase A | PPIA | 2.359 | 5.249 | 0.371 |  |
| P62942 | Peptidyl-prolyl cis-trans isomerase FKBP1A | FKBP1A | 6.158 | 12.377 | 0.067 |  |
| P63261 | Actin, cytoplasmic 2 | ACTG1 | 4.713 | 8.69 | 1.713 |  |
| P78417 | Glutathione S-transferase omega-1 | GSTO1 | 2.586 |  | -4.711 | 7.646 |
| X6R8F3 | Neutrophil gelatinase-associated lipocalin | LCN2 | -1.229 | 2.176 | -0.326 |  |
| P80511 | Protein S100-A12 | S100A12 | -2.165 | 2.164 | 2.379 |  |
| P80723 | Brain acid soluble protein 1 | BASP1 | 1.466 | 3.011 | -0.927 |  |
| Q02413 | Desmoglein-1 | DSG1 | 1.507 |  | 3.239 | 6.108 |
| Q04721 | Neurogenic locus notch homolog protein 2 | NOTCH2 | -5.802 | 19.102 | 0.248 |  |
| Q05315 | Galectin-10 | CLC | -2.294 | 4.611 | 0.505 |  |
| Q13231-3 | Chitotriosidase-1 | CHIT1 | -1.776 | 1.749 | 0.097 |  |
| Q14161-3 | ARF GTPase-activating protein GIT2 | GIT2 | -6.53 | 19.203 | -0.281 |  |
| Q4G0N8-2 | Sodium/hydrogen exchanger 10 | SLC9C1 | -7.543 | 12.432 | -1.883 |  |
| Q4VC31 | Coiled-coil domain-containing protein 58 | CCDC58 | 2.851 | 3.507 | -0.161 |  |
| Q4VX76-2 | Synaptotagmin-like protein 3 | SYTL3 | 2.797 |  | 4.011 | 2.901 |
| Q5TEC6 | Histone H3 | HIST2H3PS2 | -2.395 | 3.139 | 0.389 |  |
| Q6WKZ4-3 | Rab11 family-interacting protein 1 | RAB11FIP1 | 3.928 |  | 5.995 | 8.906 |
| Q6ZNB6 | NF-X1-type zinc finger protein NFXL1 | NFXL1 | 3.81 | 5.591 | -0.172 |  |
| Q8N4W6 | DnaJ homolog subfamily C member 22 | DNAJC22 | -9.138 | 10.812 | -0.413 |  |
| Q8TE73 | Dynein heavy chain 5, axonemal | DNAH5 | -3.448 | 3.615 | 0.004 |  |
| Q96Q89-4 | Kinesin-like protein KIF20B | KIF20B | 3.546 | 3.454 | -0.936 |  |
| Q96QZ7-6 | Membrane-associated guanylate kinase, WW and PDZ domain-containing protein 1 | MAGI1 | -4.781 | 3.989 | 0.417 |  |
| Q9BRA2 | Thioredoxin domain-containing protein 17 | TXNDC17 | 4.874 | 9.167 | -0.138 |  |
| Q9H156 | SLIT and NTRK-like protein 2 | SLITRK2 | -5.247 | 4.915 | 3.191 |  |
| Q9ULU8 | Calcium-dependent secretion activator 1 | CADPS | 2.449 |  | -7.085 | 6.906 |
| Q9Y490 | Talin-1 | TLN1 | 4.307 | 5.877 | 0.336 |  |
| Q9Y6V0-2 | Protein piccolo | PCLO | 3.967 | 6.704 | 1.096 |  |

**Table S-3.** List of significant post-translational modified peptides (PTMs Site) of NETs samples. The Site table, obtained by MaxQuant software, contains significant quantification data for the same protein samples analyzed and the site intensity derived from singly, double, triply or higher PTMs peptides (for all PTMs detail see the data friendly available on PRIDE).

| Proteins | Protein names | Gene name | Type of  PTMs | Fold Change SLE/LN vs CTR | -Log_10_ P-value SLE/LN vs CTR | Fold Change LN vs SLE | -Log_10_ P-value LN vs SLE |
| --- | --- | --- | --- | --- | --- | --- | --- |
| H0YFY6 | Nuclear mitotic apparatus protein 1 | NUMA1 | P | -1.857 |  | 4.435 | 3.125 |
| F5H6P7 | Protein mago nashi homolog;Protein mago nashi homolog 2 | MAGOHB | P | -1.915 |  | -5.264 | 4.866 |
| J3KSB5 | Neurofibromin;Neurofibromin truncated | NF1 | P | 0.161 |  | -5.579 | 6.463 |
| Q9BXR6 | Complement factor H-related protein 5 | CFHR5 | P | 3.101 | 1.64 | -7.603 | 7.585 |
| A0A0C4DGA6 | Helicase-like transcription factor | HLTF | P | 2.6 | 1.665 | -3.444 |  |
| Q4VX76-2 | Synaptotagmin-like protein 3 | SYTL3 | P | 2.209 | 3.104 | 1.91 |  |
| A0A0U1RQV4 | Rho-associated protein kinase 1 | ROCK1 | P | 5.869 | 4.738 | -4.287 | 1.953 |
| B0YIW6 | Coatomer subunit delta | ARCN1 | P | 2.543 | 2.556 | 2.107 |  |
| Q7Z699 | Sprouty-related, EVH1 domain-containing protein 1 | SPRED1 | P | 3.288 | 2.501 | 3.759 |  |
| Q8IXQ6 | Poly [ADP-ribose] polymerase 9 | PARP9 | P | 4.353 | 3.287 | 5.707 | 4.057 |
| A0A087X0P0 | Kinesin-like protein;Centromere-associated protein E | CENPE | MO | 0.967 |  | 3.681 | 5.233 |
| P12814 | Alpha-actinin-4;Alpha-actinin-1 | ACTN4 | MO | 2.147 | 3.368 | -0.031 |  |
| P08670 | Vimentin | VIM | MO | 2.258 | 1.886 | -3.919 | 3.126 |
| P10599 | Thioredoxin | TXN | MO | 1.706 |  | 2.744 | 2.002 |
| P21333-2 | Filamin-A | FLNA | MO | 2.564 | 4.341 | 1.694 | 1.948 |
| P25786 | Proteasome subunit alpha type-1 | PSMA1 | MO | 2.751 | 2.768 | 2.917 |  |
| P35579 | Myosin-9 | MYH9 | MO | 1.239 | 1.719 | 1.66 | 1.982 |
| P52565 | Rho GDP-dissociation inhibitor 1 | ARHGDIA | MO | 3.143 | 3.288 | 4.773 | 8.275 |
| P62937 | Peptidyl-prolyl cis-trans isomerase A | PPIA | MO | 3.002 | 3.219 | -2.025 |  |
| Q96EK6 | Glucosamine 6-phosphate N-acetyltransferase | GNPNAT1 | MO | 1.265 | 1.708 | 1.787 |  |
| P06702 | Protein S100-A9 | S100A9 | MO | 1.482 | 1.84 | 1.259 |  |
| P06733-2 | Alpha-enolase | ENO1 | MO | 5.307 | 10.029 | 2.231 | 4.358 |
| A0A0C4DGA6 | Helicase-like transcription factor | HLTF | D | 2.824 | 1.829 | -3.474 |  |
| E9PJ90 | HBS1-like protein | HBS1L | D | 3.204 | 1.988 | -5.553 | 3.372 |
| P04040 | Catalase | CAT | D | 1.288 | 2.004 | 1.533 |  |
| P04083 | Annexin A1 | ANXA1 | D | 1.729 | 2.005 | 1.201 | 1.605 |
| P04406 | Glyceraldehyde-3-phosphate dehydrogenase | GAPDH | D | -1.563 | 2.421 | 1.318 |  |
| P05164-2 | Myeloperoxidase | MPO | D | -3.747 | 3.838 | 2.926 |  |
| P06702 | Protein S100-A9 | S100A9 | D | 2.667 | 3.326 | 1.798 |  |
| P07737 | Profilin-1 | PFN1 | D | 2.015 | 2.909 | -0.177 |  |
| P08133-2 | Annexin A6;Annexin | ANXA6 | D | 2 | 2.886 | 2.533 | 3.026 |
| P11142 | Heat shock cognate 71 kDa protein | HSPA8 | D | 1.996 | 2.138 | 2.005 |  |
| P13796 | Plastin-2 | LCP1 | D | 1.969 | 3.528 | 1.527 | 2.244 |
| P23284 | Peptidyl-prolyl cis-trans isomerase B | PPIB | D | 1.123 | 2.087 | 0.729 |  |
| P37837 | Transaldolase | TALDO1 | D | 2.165 | 4.151 | 0.845 |  |
| P41218 | Myeloid cell nuclear differentiation antigen | MNDA | D | -3.504 | 5.716 | 2.473 | 2.986 |
| P63261 | Actin, cytoplasmic 2 | ACTG1 | D | 4.668 | 7.708 | 1.762 |  |
| X6R8F3 | Neutrophil gelatinase-associated lipocalin | LCN2 | D | -1.303 | 1.883 | -1.88 | 1.943 |
| Q7Z699 | Sprouty-related, EVH1 domain-containing protein 1 | SPRED1 | D | 3.2 | 2.55 | 3.567 |  |
| H0YFY6 | Nuclear mitotic apparatus protein 1 | NUMA1 | D | -2.479 | 1.616 | 4.508 | 3.297 |
| A0A0U1RQV4 | Rho-associated protein kinase 1 | ROCK1 | D | 5.57 | 4.434 | -4.372 | 1.95 |
| P02795 | Metallothionein-2 | MT2A | D | 2.456 | 1.794 | -5.196 | 4.945 |
| P06702 | Protein S100-A9 | S100A9 | D | -0.184 |  | 3.513 | 2.514 |
| P35579 | Myosin-9 | MYH9 | D | 1.73 |  | -4.434 | 3.392 |
| Q8IXQ6 | Poly [ADP-ribose] polymerase 9 | PARP9 | D | 3.978 | 2.668 | 5.631 | 3.295 |
| A0A087X0P5 | Alkaline phosphatase | ALPPL2 | D | 1.554 | 2.124 | 1.681 |  |
| A0A0A0MRY2 | Zinc finger MYND domain-containing protein 11 | ZMYND11 | D | 2.792 | 2.943 | 3.896 | 5.179 |
| F5H6P7 | Protein mago nashi homolog;Protein mago nashi homolog 2 | MAGOHB | D | -1.885 |  | -5.21 | 5.387 |
| J3KSB5 | Neurofibromin;Neurofibromin truncated | NF1 | D | 0.272 |  | -5.729 | 7.151 |
| P06702 | Protein S100-A9 | S100A9 | D | 1.24 | 1.836 | 1.055 |  |
| Q96NL6 | Sodium channel and clathrin linker 1 | SCLT1 | D | 1.976 | 2.274 | 2.113 |  |
| Q9H156 | SLIT and NTRK-like protein 2 | SLITRK2 | D | -5.499 | 5.421 | 3.076 |  |
| H0YFY6 | Nuclear mitotic apparatus protein 1 | NUMA1 | D | 4.018 | 2.109 | -6.94 | 3.535 |
| A0A087X0P0 | Kinesin-like protein;Centromere-associated protein E | CENPE | D | 1.219 |  | 3.525 | 5.136 |
| P0CF75 | Endogenous Bornavirus-like nucleoprotein 1 | EBLN1 | D | 5.526 | 2.381 | 9.768 | 4.902 |
| Q9H156 | SLIT and NTRK-like protein 2 | SLITRK2 | D | -5.299 | 5.349 | 2.909 |  |
| P06702 | Protein S100-A9 | S100A9 | D | -0.795 |  | 7.509 | 2.259 |
| Q13609-2 | Deoxyribonuclease gamma | DNASE1L3 | Other | 0.425 |  | 8.941 | 3.094 |
| Q8TE73 | Dynein heavy chain 5, axonemal | DNAH5 | D | 4.011 |  | -8.819 | 1.873 |
| C9JUY1 | AF4/FMR2 family member 3 | AFF3 | P | -3.191 | 2.934 | 0.144 |  |
| Q96M20-3 | Cyclic nucleotide-binding domain-containing protein 2 | CNBD2 | P | -4.99 | 5.161 |  |  |
| Q96M20-3 | Cyclic nucleotide-binding domain-containing protein 2 | CNBD2 | P | -5.357 | 5.945 |  |  |
| P07954-2 | Fumarate hydratase, mitochondrial | FH | P | -2.05 | 4.378 |  |  |
| Q14161-3 | ARF GTPase-activating protein GIT2 | GIT2 | P | -6.234 | 15.327 |  |  |
| P51858-2 | Hepatoma-derived growth factor | HDGF | P | 3.344 | 3.407 | -1.454 |  |
| A0A087X1N7 | Nebulin | NEB | P | -1.044 | 2.15 |  |  |
| A0A087WVT9 | Nucleoside diphosphate kinase | NME4 | P | 6.818 | 3.895 | 0.807 |  |
| Q04721 | Neurogenic locus notch homolog protein 2 | NOTCH2 | P | -5.624 | 14.481 |  |  |
| P04150-7 | Glucocorticoid receptor | NR3C1 | P | -6.371 | 8.589 |  |  |
| H0YJT6 | Protein NRDE2 homolog | NRDE2 | P | 5.013 | 5.038 | -1.127 |  |
| K7EJC1 | 26S proteasome non-ATPase regulatory subunit 8 | PSMD8 | P | -1.435 | 2.393 |  |  |
| F6S289 | Pentatricopeptide repeat-containing protein 2, mitochondrial | PTCD2 | P | -2.859 | 3.247 |  |  |
| Q4G0N8-2 | Sodium/hydrogen exchanger 10 | SLC9C1 | P | -7.786 | 13.652 |  |  |
| E9PJ32 | Transgelin | TAGLN | P | -4.544 | 3.505 |  |  |
| E9PP60 | GDP-L-fucose synthase | TSTA3 | P | -3.603 | 7.297 |  |  |
| Q15928 | Zinc finger protein 141 | ZNF141 | P | 7.684 | 13.059 | -0.661 |  |
| Q5SZC9 | Costars family protein ABRACL | ABRACL | MO | 2.471 | 3.277 | 0.402 |  |
| P63261 | Actin, cytoplasmic 2;Actin, cytoplasmic 2, N-terminally processed | ACTG1 | MO | 5.488 | 10.843 | 1.419 |  |
| P52565 | Rho GDP-dissociation inhibitor 1 | ARHGDIA | MO | 4.662 | 7.183 | 1.19 |  |
| Q01518-2 | Adenylyl cyclase-associated protein 1 | CAP1 | MO | -2.91 | 4.26 |  |  |
| P40121 | Macrophage-capping protein | CAPG | MO | 3.921 | 2.434 | -2.322 |  |
| P07108 | Acyl-CoA-binding protein | DBI | MO | 3.94 | 4.328 | 1.322 |  |
| P08246 | Neutrophil elastase | ELANE | MO | -2.606 | 2.276 | -0.093 |  |
| P06733-2 | Alpha-enolase | ENO1 | MO | 4.133 | 5.453 | -1.991 |  |
| P11678 | Eosinophil peroxidase | EPX | MO | -6.24 | 6.366 | 0.268 |  |
| A0A0U1RQL8 | Gelsolin | GSN | MO | 2.462 | 3.213 | -1.717 |  |
| B2R4S9 | Histone H2B | HIST1H2B | MO | -2.039 | 3.15 | 1.508 |  |
| P61604 | 10 kDa heat shock protein, mitochondrial | HSPE1 | MO | 2.208 | 2.168 | 0.806 |  |
| M0R0S5 | Mast cell-expressed membrane protein 1 | MCEMP1 | MO | -1.009 | 1.767 | 0.727 |  |
| A0A087WV66 | Antigen KI-67 | MKI67 | MO | -5.316 | 7.367 |  |  |
| P26038 | Moesin;Radixin | MSN | MO | 3.168 | 6.216 | 0.932 |  |
| P14543-2 | Nidogen-1 | NID1 | MO | -3.11 | 4.89 | 0.707 |  |
| A0A087WVT9 | Nucleoside diphosphate kinase | NME4 | MO | 6.142 | 3.044 | 1.89 |  |
| A0A0A0MR61 | Serine protease 57 | PRSS57 | MO | -2.386 | 3.792 |  |  |
| K7EJC1 | 26S proteasome non-ATPase regulatory subunit 8 | PSMD8 | MO | -1.041 | 1.393 |  |  |
| P10153 | Non-secretory ribonuclease | RNASE2 | MO | 4.412 | 5.061 | -0.185 |  |
| E9PPU1 | 40S ribosomal protein S3 | RPS3 | MO | 4.258 | 6.758 | -0.264 |  |
| C9J7N5 | Serpin I2 | SERPINI2 | MO | -1.132 | 1.376 | 0.626 |  |
| A0A087WV23 | SH3 domain-binding glutamic acid-rich-like protein 3 | SH3BGRL3 | MO | 3.621 | 3.721 | -1.539 |  |
| X6RDF7 | Solute carrier family 22 member 7 | SLC22A7 | MO | -1.514 | 2.413 |  |  |
| E9PJ32 | Transgelin | TAGLN | MO | -4.868 | 4.027 |  |  |
| Q03518 | Antigen peptide transporter 1 | TAP1 | MO | -3.58 | 2.071 | -4.581 |  |
| Q9Y490 | Talin-1 | TLN1 | MO | 3.646 | 5.284 | 1.723 |  |
| J3KN67 | Tropomyosin alpha-1 chain | TPM1 | MO | 2.967 | 3.159 | -2.019 |  |
| P60709 | Actin, cytoplasmic 1 | ACTB | D | 5.6 | 8.776 | 0.321 |  |
| A0A087X0L4 | Interferon-inducible protein AIM2 | AIM2 | D | 7.653 | 6.66 | -1.446 |  |
| P04083 | Annexin A1 | ANXA1 | D | 2.059 | 2.193 | 1.149 |  |
| H3BS90 | Calcium-transporting ATPase | ATP2C2 | D | -1.612 | 2.655 |  |  |
| H0Y7A7 | Calmodulin | CALM2 | D | 2.314 | 2.154 | -1.298 |  |
| H0YLY0 | Serine/threonine-protein kinase MRCK beta | CDC42BPB | D | -3.488 | 8.774 |  |  |
| Q6UUV7-3 | CREB-regulated transcription coactivator 3 | CRTC3 | D | -1.593 | 2.71 |  |  |
| P08311 | Cathepsin G | CTSG | D | -2.031 | 2.397 | 0.25 |  |
| Q8TE73 | Dynein heavy chain 5, axonemal | DNAH5 | D | -3.466 | 4.176 | 1.434 |  |
| K7EQT1 | Fizzy-related protein homolog | FZR1 | D | 2.889 | 3.548 | -1.384 |  |
| Q8TDN1 | Potassium voltage-gated channel subfamily G member 4 | KCNG4 | D | -3.922 | 3.739 |  |  |
| Q04721 | Neurogenic locus notch homolog protein 2 | NOTCH2 | D | -5.225 | 13.641 |  |  |
| P04150-7 | Glucocorticoid receptor | NR3C1 | D | -6.309 | 10.17 |  |  |
| H0YJT6 | Protein NRDE2 homolog | NRDE2 | D | 5.013 | 5.038 | -1.127 |  |
| A0A087WX70 | Protocadherin-15 | PCDH15 | D | -3.641 | 3.933 | -1.579 |  |
| H3BTN5 | Pyruvate kinase;Pyruvate kinase PKM | PKM | D | 2.623 | 2.863 | 0.23 |  |
| P62937 | Peptidyl-prolyl cis-trans isomerase | PPIA | D | 4.534 | 6.251 | 0.515 |  |
| Q13029-5 | PR domain zinc finger protein 2 | PRDM2 | D | -3.852 | 3.79 |  |  |
| K7EJC1 | 26S proteasome non-ATPase regulatory subunit 8 | PSMD8 | D | -1.06 | 2.062 |  |  |
| A4FU16 | Rhomboid-related protein 3 | RHBDL3 | D | -6.259 | 12.101 |  |  |
| P12724 | Eosinophil cationic protein | RNASE3 | D | -2.041 | 3.215 | 0.388 |  |
| P06702 | Protein S100-A9 | S100A9 | D | 1.933 | 4.387 | 0.622 |  |
| A0A096LPD5 | Serpin B11 | SERPINB11 | D | -4.282 | 8.275 |  |  |
| J3QRP6 | Na(+)/H(+) exchange regulatory cofactor NHE-RF1 | SLC9A3R1 | D | 4.273 | 4.832 | -1.776 |  |
| Q9H156 | SLIT and NTRK-like protein 2 | SLITRK2 | D | -7.267 | 7.279 |  |  |
| P37837 | Transaldolase | TALDO1 | D | 1.3 | 2.507 | 0.379 |  |
| Q7Z2W7-4 | Transient receptor potential cation channel subfamily M member 8 | TRPM8 | D | 6.318 | 5.009 | -1.146 |  |
| E9PP60 | GDP-L-fucose synthase | TSTA3 | D | -3.576 | 6.841 |  |  |
| K7EIR6 | Zinc finger protein 233 | ZNF112 | D | -3.011 | 2.759 |  |  |
| Q05315 | Eosinophil lysophospholipase | CLC | Other | -7.279 | 3.374 | -0.99 |  |
| Q13609-2 | Deoxyribonuclease gamma | DNASE1L3 | Other | 11.055 | 6.346 | -0.374 |  |
| P62805 | Histone H4 | HIST1H4A | Other | -9.504 | 5.533 | 1.295 |  |
